# Predicting Biological Functions of Compounds Based on Chemical-Chemical Interactions

**DOI:** 10.1371/journal.pone.0029491

**Published:** 2011-12-29

**Authors:** Le-Le Hu, Chen Chen, Tao Huang, Yu-Dong Cai, Kuo-Chen Chou

**Affiliations:** 1 Institute of Systems Biology, Shanghai University, Shanghai, China; 2 Department of Chemistry, College of Sciences, Shanghai University, Shanghai, China; 3 Key Laboratory of Systems Biology, Shanghai Institutes for Biological Sciences, Chinese Academy of Sciences, Shanghai, China; 4 Shanghai Center for Bioinformation Technology, Shanghai, China; 5 Gordon Life Science Institute, San Diego, California, United States of America; Hospital for Sick Children, Canada

## Abstract

Given a compound, how can we effectively predict its biological function? It is a fundamentally important problem because the information thus obtained may benefit the understanding of many basic biological processes and provide useful clues for drug design. In this study, based on the information of chemical-chemical interactions, a novel method was developed that can be used to identify which of the following eleven metabolic pathway classes a query compound may be involved with: (1) Carbohydrate Metabolism, (2) Energy Metabolism, (3) Lipid Metabolism, (4) Nucleotide Metabolism, (5) Amino Acid Metabolism, (6) Metabolism of Other Amino Acids, (7) Glycan Biosynthesis and Metabolism, (8) Metabolism of Cofactors and Vitamins, (9) Metabolism of Terpenoids and Polyketides, (10) Biosynthesis of Other Secondary Metabolites, (11) Xenobiotics Biodegradation and Metabolism. It was observed that the overall success rate obtained by the method via the 5-fold cross-validation test on a benchmark dataset consisting of 3,137 compounds was 77.97%, which is much higher than 10.45%, the corresponding success rate obtained by the random guesses. Besides, to deal with the situation that some compounds may be involved with more than one metabolic pathway class, the method presented here is featured by the capacity able to provide a series of potential metabolic pathway classes ranked according to the descending order of their likelihood for each of the query compounds concerned. Furthermore, our method was also applied to predict 5,549 compounds whose metabolic pathway classes are unknown. Interestingly, the results thus obtained are quite consistent with the deductions from the reports by other investigators. It is anticipated that, with the continuous increase of the chemical-chemical interaction data, the current method will be further enhanced in its power and accuracy, so as to become a useful complementary vehicle in annotating uncharacterized compounds for their biological functions.

## Introduction

Metabolism refers to a collection of chemical reactions in vivo, which keep an unceasing supply of matter and energy for living organisms to maintain life (e.g., growth and reproduction) [1]. These energy-using and energy-releasing chemical reactions catalyzed by enzymes are organized into many metabolic pathways. Some compounds/small molecules play major roles in these pathways and are vital for many activities essential for life. For example, during the digestion, the energy rich molecules (i.e. carbohydrate) are broken apart to provide energy, which is then used by cells to build up complex molecules from simple molecules, such as utilizing amino acids to synthesize new proteins that the body needs. Identifying the biological functions of compounds is an effective way to study the mechanisms of many basic biological processes [2]. On the other hand, small molecules are the cause, and the cure, for many diseases. For example, diabetes mellitus is a metabolic disease caused by insufficient or inefficient insulin secretary response and elevated blood glucose level [3]. Compounds such as sulfonylureas [4], acarbose [5], biguanides, thiazolidinediones [5], and sitagliptin [3] have been used as effective drugs for diabetic therapy. Therefore, it is essential to annotate the bioactivities of compounds, which will benefit drug design and disease treatment.

Besides the conventional biochemical experiments, computational methods are alternative ways to annotate the biological functions of compounds. In recent years, various bioinformatics and structural bioinformatics [6] tools were developed to address this issue, such as Quantitative Structure Activity Relationship (QSAR) [7], [8], pharmacophore modeling [9], molecular docking [10], and Monte Carlo simulated annealing approach [11], [12]. Different from these methods, Lu et al. [1] and Cai et al. [2] analyzed the biological functions of compounds by mapping them to the corresponding metabolic pathway classes, which are strongly associated with the biological functions of compounds. The functional group composition was used to represent the compounds, and the Nearest Neighbor Algorithm and AdaBoost learner [13] were used to construct the prediction models by Cai et al. [2] and Lu et al. [1], respectively. Both the two prediction methods achieved quite promising results on their own datasets. However, none of their datasets contained the “multi-function” compounds that belong to two or more metabolic pathway classes. Since these authors were only focused on addressing the single-label classification problem, their methods could not be used to deal with the “multi-function” compounds. Actually, according to KEGG [14], among all the compounds with functional annotations, the “multi-function” compounds occupy about 8%. Particularly, these multi-function compounds may play some unique role intriguing to both basic research and drug development and hence are worthy of our special attention.

Recently, the systems biology methods based on protein-protein interactions have been widely applied for predicting protein attributes [15], [16], [17], [18], [19]. These algorithms suggest that interactive proteins are likely to share the common biological functions [16], [17], [18], [19], also more likely tending to have the same biological function than non-interactive ones [20], [21]. Likewise, we can assume that the interactive compounds may tend to share the common biological functions. In this study, the chemical-chemical interactions were retrieved from STITCH [22] (Search tool for interactions of chemicals), where the interaction unit consists of two chemicals and their interaction weight. The interaction weight (confidence score) represents the probability that the interaction occurs between the two chemicals concerned. The interactive compounds can be classified into the following three categories: (I) ones that participate in the same reactions; (II) ones that share the similar structures or activities; (III) ones with the literature associations [22]. In a metabolism system, chemical reactions are organized into many metabolic pathways, thus the compounds involved in the same reactions are in the same metabolic pathways. Similar structures or activity means that they share the similar functions, and hence they are likely to be in the same metabolic pathways. The co-occurrence of two compounds in many literatures suggests some kinds of direct or indirect relationships, indicating they have the potential to be in the same metabolic pathways. Accordingly, it is rational to suppose that the interactive compounds tend to participate in the same metabolic pathways.

In this study, we proposed a multi-target model based on chemical-chemical interactions for predicting the metabolic pathways where compounds participate in. Our method sorts the possible metabolic pathways that are associated with the query chemical, providing a more comprehensive view of the biological effects of the compound.

According to a recent comprehensive review [23], to establish a really useful statistical predictor for a biological system, we need to consider the following procedures: (1) construct or select a valid benchmark dataset to train and test the predictor; (2) formulate the statistical samples with an effective mathematical expression that can truly reflect their intrinsic correlation with the attribute to be predicted; (3) introduce or develop a powerful algorithm (or engine) to operate the prediction; (4) properly perform cross-validation tests to objectively evaluate the anticipated accuracy of the predictor. Below, let us describe how to deal with these steps.

## Materials and Methods

### Benchmark Dataset

The compounds were retrieved from public available database KEGG [14] (Kyoto Encyclopedia of Genes and Genomes) compound [ftp://ftp.genome.jp/pub/kegg/release/archive/kegg/42/ligand.tar.gz] (release 42.0). Subsequently, these compounds were mapped to the following 11 major metabolic pathway classes that are strongly associated with the biological functions of compounds (http://www.genome.jp/kegg/pathway.html#metabolism): (1) Carbohydrate Metabolism, (2) Energy Metabolism, (3) Lipid Metabolism, (4) Nucleotide Metabolism, (5) Amino Acid Metabolism, (6) Metabolism of Other Amino Acids, (7) Glycan Biosynthesis and Metabolism, (8) Metabolism of Cofactors and Vitamins, (9) Metabolism of Terpenoids and Polyketides, (10) Biosynthesis of Other Secondary Metabolites, (11) Xenobiotics Biodegradation and Metabolism. After excluding those compounds without any metabolic pathway information, 4,366 compounds were collected that have clear biological functions annotated (see **Table 1** under the title of Group-I). From the 4,366 compounds of Group-I, 3,137 compounds were retrieved that can interact with any of the others as annotated by STITCH database [22] (see **Table 1** under the title of Group-II).

**Table 1:** Distribution of the 4,366 and 3,137 compounds in the 11 metabolic pathway classes.

Class code	Metabolic Pathway	Number of different compounds	
		Group-I	Group-II
	4,366	3,137	
1	Carbohydrate Metabolism	444	394
2	Energy Metabolism	129	120
3	Lipid Metabolism	610	383
4	Nucleotide Metabolism	145	132
5	Amino Acid Metabolism	563	483
6	Metabolism of Other Amino Acids	212	154
7	Glycan Biosynthesis and Metabolism	68	43
8	Metabolism of Cofactors and Vitamins	396	309
9	Metabolism of Terpenoids and Polyketides	713	499
10	Biosynthesis of Other Secondary Metabolites	722	519
11	Xenobiotics Biodegradation and Metabolism	858	570
Overall		4,860	3,606

The 4,366 compounds in Group-I were screened from KEGG by selecting the compounds with the metabolic pathway information. The 3,137 compounds in Group-II were those retrieved from the 4,366 compounds that can interact with any other as annotated by STITCH database. Note that since a compound may occur in more than one pathway class, the sum of the compounds over the 11 pathway classes is greater than the number of different compounds for the cases of both Group-I and Group-II.

Of the 4,366 compounds of Group-I, 4,027 are involved in only one metabolic pathway class, 246 in two metabolic pathway classes, 54 in three metabolic pathway classes, 24 in four metabolic pathway classes, 9 in five metabolic pathway classes, 4 in six metabolic pathway classes, 2 in seven metabolic pathway classes, and none in eight or more metabolic pathway classes. Of the 3,137 compounds of Group-II, 2,820 are involved in only one metabolic pathway class, 226 in two metabolic pathway classes, 53 in three metabolic pathway classes, 23 in four metabolic pathway classes, 9 in five metabolic pathway classes, 4 in six metabolic pathway classes, 2 in seven metabolic pathway classes, and none in eight or more metabolic pathway classes.

Note that since one compound may occur in more than one pathway class, the sum of the compounds over the 11 pathway classes in Group-I turns out to be 4,860, which is greater than 4,366. Likewise, the sum of the compounds over the 11 pathway classes in Group-II is 3,606, which is greater than 3,137. This is quite similar to the case of proteins with multiple location sites, as elaborated in [24], [25].

The chemicals interactions were retrieved from STITCH [22], a large database of known and predicted interactions of chemicals and proteins derived from experiments, literature, databases, and so on. As mentioned in Introduction, there are three types of associations between two compounds in STITCH: (I) co-occurrence in reactions, (II) similar structures or activities, and (III) literature associations. In the downloaded STITCH chemicals interactions file: chemical_chemical.links.detailed.v2.0.tsv from http://stitch.embl.de/cgi/show_download_page.pl, there are 337,482 pairs of interactive compounds belonging solely to type I, 73,598 pairs solely in type II, 2,152,508 pairs solely in type III, 384 pairs in both type I and II, 120,936 pairs in both type I and III, 10,372 pairs in both type II and III, and 1,990 pairs in the three types, in total of 2,697,270 interactions. Each of the interaction is quantified by the interaction confidence score, which represents the likelihood that the interaction occurs. In this study, the interactions with both interactive compounds occurring in the 4,366 compounds of Group-I were extracted. As a result, 3,137 compounds with 75,949 interactions were collected to constitute the benchmark dataset of the current study (see **Table 1** under the title of Group-II).

Besides the 4,366 compounds (cf. **Table 1** under the title of Group-I) with known metabolic pathway classes, there are 11,661 compounds without known metabolic pathway classes in KEGG. Among these compounds, 5,549 compounds that have annotated interactions with the compounds of the 4,366 compounds in STITCH were collected. Such 5,549 compounds are to form an independent dataset, being used to test our prediction method in hopes to acquire useful information for further investigation.

### Method

As mentioned in Introduction, the interactive compounds tend to participate in the same metabolic pathways. Accordingly, for a query compound, the higher interaction confidence score with its interactive compound, the more likely they are to participate in the same metabolic pathway. The more its interactive compounds involving in a certain metabolic pathway, the more likely it is to participate in such metabolic pathway. Based on these points, we should count not only the number of compounds interacting with the query compound, but also the corresponding interaction scores. Thus, the desired predictor can be formulated via the following procedures.

Suppose the training dataset contains 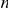 compounds, which are denoted as 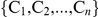. The 11 metabolic pathway classes (cf. **Table 1**) are expressed as 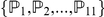, where 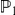 represents the 1^st^ metabolic pathway class (“Carbohydrate Metabolism”), 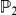 the 2^nd^ metabolic pathway class (“Energy Metabolism”), 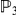 the 3^rd^ metabolic pathway class (“Lipid Metabolism”), and so forth. Thus, the descriptor of metabolic pathway classes to which the compound 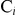 belongs to can be formulated as
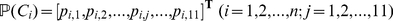
(1)where
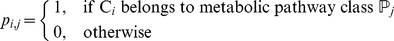
(2)Given a query compound 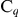, its interaction with the compounds in the training dataset can be defined as
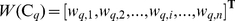
(3)where 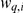 represents the interaction confidence score between 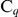 and 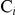. 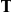 is the transpose operator, and 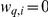 if no interaction exists between them. Here, we did not consider the self-interaction, therefore 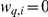 when 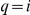. Accordingly, the likelihood that the query compound 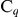 is involved in the *j*-th metabolic pathway class can be formulated by the following score
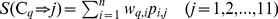
(4)which is the sum of the interaction confidence scores of 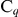 with its interactive compounds in the training dataset by counting both the number of interactive compounds and the interaction confidence scores. Obviously, the higher the score of Eq. 4, the more likely 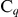 is to be involved in the *j*-th metabolic pathway 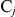. Thus, for a given query compound 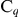, we can use Eq. 4 to calculate its 11 scores, with each associated with one of the 11 metabolic pathway classes. The class to which the compound 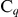 most likely belongs should be the one with the highest score. In other words, the query compound 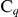 will be predicted to belong to the 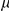
th metabolic pathway class if
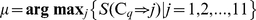
(5)where 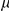 is the argument of *j* that maximize the value of 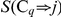. Since the problem in this study is of multi-label classification, we intend to provide flexible information by predicting some candidate metabolic pathway classes for the query compounds, rather than just the most likely metabolic pathway class. Therefore, instead of Eq. 5, let us consider the following equation containing 11 scores in a one-column vector:
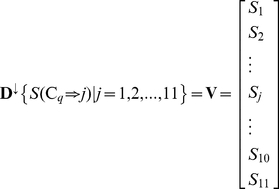
(6)where 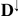 is a descending operator that sorts the 11 scores of Eq. 4 for 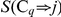 according to the descending order (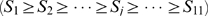). If there is a tie among these scores, a random order will be made among those with a tie. Consequently, the predicted metabolic pathway classes for the query compound can be derived according to the descending order of Eq. 6; i.e., if 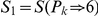, 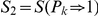, 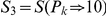, then it follows that the query compound 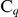 is involved in the 6^th^ metabolic pathway class (“Metabolism of Other Amino Acids”) will be ranked as the highest in the likelihood, that 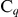 in the 1^st^ metabolic pathway class (“Carbohydrate Metabolism”) as the 2^nd^, and that 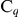 in the 10^th^ metabolic pathway class (“Biosynthesis of Other Secondary Metabolites”) as the 3^rd^. The corresponding results thus obtained are, respectively, called the 1^st^-order, 2^nd^-order, and 3^rd^-order predicted metabolic pathway classes. And so forth.

### Cross-Validation

In statistical prediction, the following three cross-validation methods are often used to examine a predictor for its effectiveness in practical application: independent dataset test, subsampling (such as 5-fold, 7-fold, or 10-fold cross-validation) test, and jackknife test [26]. In this study, the 5-fold cross-validation was employed to examine the performance of our method. The concrete procedures were that the training dataset were divided into five groups by splitting each of its subsets into five approximately equal-sized subgroups. Each of these five groups was in turn used as a testing dataset and the rest used as training dataset, thereby generating five different success rates, with their average representing the success rate by the 5-fold cross-validation.

For the *j*-th order prediction, the accuracy 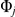 was calculated by
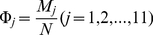
(7)where 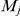 is the number of the compounds whose *j*-th order predicted metabolic pathway class is one of the true pathway classes that the compounds are involved with, and 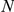 is the total number of compounds in the dataset. Such 11-order accuracies were used to evaluate our prediction method. It is obvious according to the definition of Eq. 7 that, the higher the value of 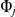 with a smaller value of 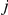, or the lower the value of 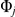 with a larger value of 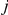, the better the prediction quality will be by our method.

In the dataset, the average number of metabolic pathway class that each compound is involved in is calculated as
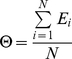
(8)where 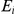 is the number of metabolic pathway classes that the compound 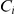 is involved with. Hence, another measurement - the likelihood that the first *k* order predicted metabolic pathway classes cover all the true metabolic pathway classes that the compound is involved in – can be formulated as
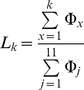
(9)Usually, 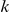 is the smallest integer equal or greater than the average number of metabolic pathway classes (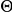). It is obvious from Eq. 9 that the larger the value of 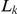, the better the prediction quality will be by our method.

### Prediction process

Given a query compound, according to the information of its interactions with the 4,366 compounds in Group-I (**Table 1**) whose metabolic pathway classes are known, the likelihood of its belonging to each of the 11 metabolic pathway classes can be easily calculated according to Eq. 4. And the scores thus obtained were sorted according to a descending order (Eq. 6) to yield the predicted metabolic pathway classes according to their different ranks or orders.

## Results and Discussion

### Evaluation Results by the 5-fold Cross-validation

**Figure 1:**
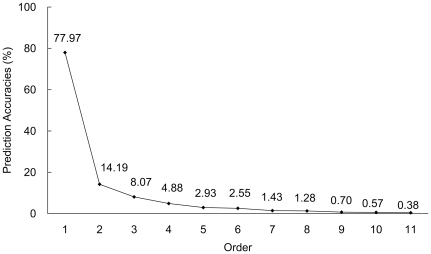
Illustration to show the accuracy by each of the 11 order predictions for the 3,137 compounds by the 5-fold cross-validation. It can be seen from the figure that from the first order to the last one, the 11 accuracies form a download-slope curve.

In this study, our method was evaluated by the 5-fold cross-validation on the benchmark dataset that contains 3,137 compounds in Group-II of **Table 1**. The 11-order prediction accuracies are shown in **Figure 1**. The first order (most likely) prediction accuracy is 77.97%, and the last order (least likely) prediction accuracy is 0.38%, which indicates a quite good performance of our method.

The average number of metabolic pathway classes with which each compound is involved is 1.15 (cf. Eq. 8), meaning that the average success rate by a random guess would be 1.15/11 = 10.45%, which is much lower than that by our method.

Accordingly, the parameter *k* in Eq. 9 was set to (1.15+1) = 2; i.e., we may select the results of the first two orders of the predicted metabolic pathway classes for the query compounds. As we can see from **Figure 1**, the accuracies of both the 1^st^ and 2^nd^ order predictions are higher than that of the random guess. According to Eq. 9 the metabolic pathway classes predicted by the 1^st^ and 2^nd^ orders have actually covered more than 80% of all the true metabolic pathway classes, suggesting that, of the results predicted by the 11 orders, more attention should be paid to those by the first two orders.

Listed in **Table 2** are the accuracies by each of the 11 prediction orders for the 3,137 compounds about their involvement in the 11 metabolic pathway classes using the 5-fold cross-validation test. The highest accuracy achieved by the 1^st^-order prediction was 80.96% for the 1^st^ metabolic pathway class (“Carbohydrate Metabolism”). And the results obtained by the 1^st^ and 2^nd^ prediction orders have covered 89.00% of the true metabolic pathway classes. The second highest accuracy by the 1^st^-order prediction was 78.77% for the 11^th^ metabolic pathway class (Xenobiotics Biodegradation and Metabolism), while the results obtained by the 1^st^ and 2^nd^ prediction orders have covered 87.00% of the true metabolic pathway classes. Both the two 1^st^-order accuracies are higher than the overall 1^st^-order prediction accuracy of 77.97%, and each of their combinations with the 2^nd^-order predictions is also higher than the overall likelihood of 80.00%. As for the metabolic pathway classes with less compounds, such as “Glycan Biosynthesis and Metabolism” class that contains only 68 compounds in Group-I and 43 in Group-II (cf. **Table 1**), the predicted accuracies were relatively not as good as the others. It is anticipated that with more experimental data are available in future for the compounds in these classes, the corresponding prediction success rates will be improved. Overall speaking, the aforementioned results are quite encouraging, indicating that our approach may become a useful tool to deal with this kind of very complicated systems.

**Table 2:** The accuracy predicted by each of the 11 orders for the metabolic pathway classes of the 3,137 compounds by the 5-fold cross-validation test.

Class code	Accuracy (%) predicted by each order										
	1^st^	2^nd^	3^rd^	4^th^	5^th^	6^th^	7^th^	8^th^	9^th^	10^th^	11^th^
1	80.96	8.38	5.08	1.78	1.02	1.27	0.25	1.02	0.00	0.25	0.00
2	31.67	30.00	18.33	7.50	4.17	3.33	0.00	3.33	0.00	0.83	0.83
3	73.89	6.27	6.27	4.44	1.57	2.61	2.35	0.52	0.52	1.31	0.26
4	65.15	11.36	6.82	5.30	3.03	1.52	2.27	0.00	4.55	0.00	0.00
5	61.70	19.88	10.97	5.38	1.04	0.83	0.21	0.00	0.00	0.00	0.00
6	29.87	27.27	11.69	11.69	5.19	5.19	3.90	3.25	1.95	0.00	0.00
7	20.93	25.58	11.63	9.30	6.98	4.65	2.33	4.65	0.00	2.33	11.63
8	61.17	17.15	9.39	4.21	3.24	2.91	0.97	0.32	0.32	0.32	0.00
9	74.35	8.42	4.01	3.41	1.20	1.20	2.20	2.40	1.00	1.20	0.60
10	68.98	8.67	4.62	3.66	5.01	4.24	1.54	1.54	0.77	0.58	0.39
11	78.77	8.42	5.09	2.81	2.63	1.40	0.35	0.35	0.18	0.00	0.00
Overall	77.97	14.19	8.07	4.88	2.93	2.55	1.43	1.28	0.70	0.57	0.38

See **Table 1** for the numbers-distribution of the 3,137 compounds among the 11 metabolic pathway classes.

As stated in the Method section, the interactive compounds derived from STITCH tend to participate in the same metabolic pathways. For example, **Table 3** lists the interactions of dihydrouracil with other compounds. Among the 32 interactive compounds, most of them appear in “metabolism of cofactors and vitamins” or “metabolism of other amino acids” or “nucleotide metabolism” pathway class (cf. **Table 1**) just like dihydrouracil. Dihydrouracil and uracil participate in pyrimidine metabolism pathway (belong to “nucleotide metabolism”), where 5,6-dihydrouracil and NADP+ are catalyzed by dihydropyrimidine dehydrogenase (DPD) to form uracil and NADPH+H+ [14], [27]. They are also co-mentioned in many PubMed Abstracts such as [28], [29], [30], [31], [32], [33], [34], [35], [36], [37]. Another two interactive compounds - dihydrouracil and dihydrothymine share a very similar structure, the only difference is that dihydrothymine has a methyl at the 5th position of the hexatomic ring while dihydrouracil has not [38]. According to the prediction criteria, when dihydrouracil was treated as a query compound, the first three order predicted metabolic pathways that it participates in are “nucleotide metabolism”, “metabolism of cofactors and vitamins” and “metabolism of other amino acids”, respectively, which are consistent with the true metabolic pathways that it is involved in.

**Table 3:** Interactions of dihydrouracil with other compounds in the benchmark dataset of Group-II.

KEGG ligand	Name	Code of Metabolic pathway class	KEGG ligand	Name	Code of Metabolic pathway class	Interaction confidence
C00429	Dihydrouracil	4, 6, 8	C00106	Uracil	4, 6, 8	0.981
C00429	Dihydrouracil	4, 6, 8	C02642	N-carbamoyl-be.	4, 6, 8	0.945
C00429	Dihydrouracil	4, 6, 8	C00006	NADP	2, 6, 8	0.921
C00429	Dihydrouracil	4, 6, 8	C00005	NADP(H)	2, 6	0.902
C00429	Dihydrouracil	4, 6, 8	C00001	Hydroxyl radic.	2, 8	0.899
C00429	Dihydrouracil	4, 6, 8	C00013	Pyrophosphate	2	0.899
C00429	Dihydrouracil	4, 6, 8	C00119	Phosphoribosyl.	1, 4, 5	0.899
C00429	Dihydrouracil	4, 6, 8	C00906	Dihydrothymine	4	0.855
C00429	Dihydrouracil	4, 6, 8	C00178	Thymine	4	0.814
C00429	Dihydrouracil	4, 6, 8	C07649	5-fluorouracil	11	0.744
C00429	Dihydrouracil	4, 6, 8	C00099	Beta-alanine	1, 4, 6, 8	0.650
C00429	Dihydrouracil	4, 6, 8	C00380	Cytosine	4	0.551
C00429	Dihydrouracil	4, 6, 8	C00262	Hypoxanthine	4	0.436
C00429	Dihydrouracil	4, 6, 8	C00299	Uridine	4	0.433
C00429	Dihydrouracil	4, 6, 8	C00295	Orotic acid	4	0.386
C00429	Dihydrouracil	4, 6, 8	C05145	Beta-aminoisob.	4	0.362
C00429	Dihydrouracil	4, 6, 8	C02067	Pseudouridine	4	0.353
C00429	Dihydrouracil	4, 6, 8	C00881	Deoxycytidine	4	0.350
C00429	Dihydrouracil	4, 6, 8	C00147	Adenine	4, 9	0.308
C00429	Dihydrouracil	4, 6, 8	C05100	Beta-ureidoiso.	4	0.286
C00429	Dihydrouracil	4, 6, 8	C03056	2,6-dihydroxyp.	8	0.274
C00429	Dihydrouracil	4, 6, 8	C02565	N-methylhydant.	5	0.272
C00429	Dihydrouracil	4, 6, 8	C00337	Dihydroorotate	4	0.262
C00429	Dihydrouracil	4, 6, 8	C00757	Berberine	10	0.252
C00429	Dihydrouracil	4, 6, 8	C00222	Malonate semia.	1, 11, 6	0.218
C00429	Dihydrouracil	4, 6, 8	C12650	Capecitabine	11	0.214
C00429	Dihydrouracil	4, 6, 8	C12673	Tegafur	11	0.210
C00429	Dihydrouracil	4, 6, 8	C00522	Pantoate	8	0.207
C00429	Dihydrouracil	4, 6, 8	C00864	Pantothenic ac.	6, 8	0.205
C00429	Dihydrouracil	4, 6, 8	C11736	FUdR	11	0.199
C00429	Dihydrouracil	4, 6, 8	C00366	Uric acid	4	0.167
C00429	Dihydrouracil	4, 6, 8	C00219	Arachidonic ac.	3	0.154

See **Table 1** for the code of the metabolic pathway class.

### Predicted results for the compounds with unknown metabolic pathway

Encouraged by the quite promising results obtained by the 5-fold cross-validation test on the benchmark dataset of the 3,137 compounds, we applied the method to the 5,549 compounds whose metabolic pathways are unknown as mentioned in the Materials and Methods section. The predicted results thus obtained are given in **Table S1**. As discussed above, we selected the metabolic pathway classes obtained by the 1^st^ and 2^nd^ order predictions for these compounds, in hoping that the information thus obtained may provide useful clues for further investigations. Actually, it is interesting to see that many of our predicted results have proved to be reasonable according to the reports from other investigators. For example, N-acetylgalactosamine 4-sulfate and its interactive compounds with pathway information are shown in **Table 4**. N-acetylgalactosamine 4-sulfate can bind to sulfate, glucuronic acid, galactose, xylose, fucose, Na(+), glycerol, and phosphate to form complex to perform the biological function [39]. In PubMed Abstracts, N-acetylgalactosamine 4-sulfate is co-mentioned with sulfate [40], glucuronic acid [41], galactose [42], 3′-phospho.pho. [43], sugar-1-phosph. [44], UDP-GlcNAc [45], indole-3-glyce. [46], N-acetyl-D-glucosamine [47], and GDP-mannose [44]. Besides, N-acetylgalactosamine 4-sulfate and N-acetyl-D-glucosamine share a similar structure and the difference is that N-acetylgalactosamine 4-sulfate has a sulfate at the position 4 of the ring while N-acetyl-D-glucosamine has not [38]. From these evidences, N-acetylgalactosamine 4-sulfate is supposed to participate in the same metabolic pathways as its interactive compounds. It can be seen from **Table 4** that most of the interactive compounds of N-acetylgalactosamine 4-sulfate belong to the 1^st^ and 2^nd^ metabolic pathway classes. By considering all the interactions and the interaction confidence scores, it was predicted that Carbohydrate Metabolism (the 1^st^ class) and Energy Metabolism (the 2^nd^ class) would be the possible metabolic pathway classes that N-acetylgalactosamine 4-sulfate belongs to. Actually, as a carbohydrate, N-acetylgalactosamine 4-sulfate reacts with Chondroitin 4-sulfate to form hydrogen oxide and G12336 (i.e. (GalNAc)_2_(GlcA)_1_(S)_2_), one kind of glycan which can participate in Carbohydrate and Energy Metabolism. Therefore, N-acetylgalactosamine 4-sulfate may also participate in Carbohydrate and Energy Metabolism. Another example is that cyclopropylamine in **Table 4** has 23 interactive compounds with known pathway information. Cyclopropylamine, cyanuric acid, ammonia, N-cyclopropylammelide, c0761, hydroxyl radicals are in the same pathway - N-cyclopropylmelamine degradation [48], [49], where N-cyclopropylmelamine first reacts with hydrogen oxide to form N-cyclopropylammeline and ammonia, and then N-cyclopropylammeline also reacts with hydrogen oxide to form N-cyclopropylammelide and ammonia. After that, N-cyclopropylammelide reacts with hydrogen oxide to form cyanuric acid, cyclopropylamine and hydroxyl radicals. Finally, cyanuric acid is transformed into hydrogen oxide and ammonia through cyanurate degradation. Cyanuric acid, N-cyclopropylammelide, and c0761 are all in the 11^th^ pathway class. Therefore, cyclopropylamine may also belong to the 11^th^ pathway class (Xenobiotics Biodegradation and Metabolism). For other interactive compounds, they are co-mentioned with cyclopropylamine in PubMed Abstracts, such as polyethylene [50], 1-aminocyclopropane-1-carboxylic acid [51], cyclopropanecarboxylic acid [52], 3-hydroxyphenylacetic acid [53], and acetophenone [54]. In **Table 4**, most of the interactive compounds of cyclopropylamine belong to the 11^th^ metabolic pathway classes. According to above analysis, cyclopropylamine is suggested to participate in the Xenobiotics Biodegradation Metabolism, which was the 1^st^-order predicted class for cyclopropylamine by our method. Accordingly, it is quite reasonable to expect that our method may provide useful information for further investigating into biological functions of compounds from the viewpoint of system biology.

**Table 4:** Interactions of N-acetylgalactosamine 4-sulfate and cyclopropylamine with other compounds whose metabolic pathway classes are known.

KEGG Ligand	Name	KEGG Ligand	Name	Code of Metabolic pathway class	Interaction Confidence
C16265	N-acetylgalactosamine 4-sulfate	C00059	sulfate	2, 4, 5	0.956
C16265	N-acetylgalactosamine 4-sulfate	C00333	glucuronic acid	1	0.931
C16265	N-acetylgalactosamine 4-sulfate	C15923	galactose	1	0.904
C16265	N-acetylgalactosamine 4-sulfate	C01508	xylose	1	0.9
C16265	N-acetylgalactosamine 4-sulfate	C01721	fucose	1	0.899
C16265	N-acetylgalactosamine 4-sulfate	C01330	Na(+)	2	0.899
C16265	N-acetylgalactosamine 4-sulfate	C00116	glycerol	1, 3	0.899
C16265	N-acetylgalactosamine 4-sulfate	C00009	phosphate	2, 7	0.899
C16265	N-acetylgalactosamine 4-sulfate	C00053	3′-phospho.pho.	2, 4, 7	0.47
C16265	N-acetylgalactosamine 4-sulfate	C02591	sugar-1-phosph.	1	0.312
C16265	N-acetylgalactosamine 4-sulfate	C01170	UDP-GlcNAc	1	0.27
C16265	N-acetylgalactosamine 4-sulfate	C03506	indole-3-glyce.	10, 5	0.256
C16265	N-acetylgalactosamine 4-sulfate	C01132	N-acetyl-D-glucosamine	1	0.235
C16265	N-acetylgalactosamine 4-sulfate	C00096	GDP-mannose	1, 7	0.183
C14150	cyclopropylamine	C06554	cyanuric acid	11	0.918
C14150	cyclopropylamine	C00014	ammonia	2, 4, 5, 6	0.907
C14150	cyclopropylamine	C14149	N-cyclopropylammelide	11	0.899
C14150	cyclopropylamine	C14148	c0761	11	0.899
C14150	cyclopropylamine	C00001	hydroxyl radicals	2, 8	0.899
C14150	cyclopropylamine	C00969	reuterin	3	0.378
C14150	cyclopropylamine	C06547	polyethylene	11, 5	0.347
C14150	cyclopropylamine	C01234	1-aminocyclopropane-1-carboxylic acid	1, 5	0.29
C14150	cyclopropylamine	C00218	methylamine	2	0.29
C14150	cyclopropylamine	C16267	cyclopropanecarboxylic acid	11	0.286
C14150	cyclopropylamine	C16318	methyl jasmonate	3	0.273
C14150	cyclopropylamine	C11512	methyl jasmonate	3	0.273
C14150	cyclopropylamine	C05593	3-hydroxyphenylacetic acid	11, 5	0.267
C14150	cyclopropylamine	C00261	benzaldehyde	11	0.238
C14150	cyclopropylamine	C01054	2,3-oxidosqualene	3	0.236
C14150	cyclopropylamine	C01013	3-hydroxypropionate	1, 6	0.224
C14150	cyclopropylamine	C01471	acrolein	11	0.208
C14150	cyclopropylamine	C01746	calcium channel blocker	10	0.205
C14150	cyclopropylamine	C00571	cyclohexylamine	11	0.205
C14150	cyclopropylamine	C00144	guanosine monophosphate	4	0.205
C14150	cyclopropylamine	C00903	cinnamaldehyde	10	0.171
C14150	cyclopropylamine	C07113	acetophenone	11	0.168
C14150	cyclopropylamine	C01724	lanosterol	3	0.152

See **Table 1** for the code of the metabolic pathway class.

### Application and improvement

As indicated by the above discussion and analysis, the results derived from the 1^st^ and 2^nd^ order predictions should be considered as the candidates for the metabolic pathway classes with which the query compound may be involved. In view of this, biochemical experiments should be conducted by mainly focusing on the targets predicted by the 1^st^ and 2^nd^ order predictions. The results obtained by the last five order predictions can be ignored due to their very low likelihood (<2%). Consequently, the current prediction method can provide useful clues for further validation by experiments and expedite the research progress by prioritizing the targets concerned.

It is instructive to note that for the 4,366 compounds in Group-I of **Table 1**, there are still 1,229 compounds that can not be processed by the current method due to lack of the interaction information with other compounds within the dataset. It is expected that the problem can be solved by collecting as much chemical-chemical interaction information as possible from STITCH, which is a large-scale and well-maintained resource in chemical biology, including the interactions information for over 2.5 million proteins and over 74,000 small molecules in 630 organisms. With the continuous increase of the interactions information, the performance of our method will be further improved.

### Conclusion

Based on the chemical-chemical interactions information, a multi-target model was proposed for identifying the metabolic pathway classes with which a query compound is involved. Since some compounds may be involved with more than one metabolic pathway class, our method is featured by the capacity able to provide a series of potential metabolic pathway classes for each of the query compounds investigated, instead of only one metabolic pathway class. It is anticipated that our method may become a useful tool in helping annotate the compound for their biological functions.

## Supporting Information

Table S1 Each order predicted metabolic pathway class for the collected 5,549 compounds without known metabolic pathway classes. The predicted metabolic pathway class code corresponds to the code in Table 1. Among the 11 predicted pathway classes, the first 2 order predicted metabolic pathway classes should be paid more attention to.
(PDF)
