# Supplementary material for: Predicting Biological Functions of Compounds Based on Chemical-Chemical Interactions
Source: PLoS One. 2011 Dec 29;6(12):e29491. doi: 10.1371/journal.pone.0029491 (PMC3248422; doi:10.1371/journal.pone.0029491)
Supplement: Table S1 — Each order predicted metabolic pathway class for the collected 5,549 compounds without known metabolic pathway classes. The predicted metabolic pathway class code corresponds to the code in Table 1. Among the 11 predicted pathway classes, the first 2 order predicted metabolic pathway classes should be paid more attention to. (PDF) [file pone.0029491.s001.pdf]

Table S1. Each order predicted metabolic pathway class for the collected 5,549 compounds without known metabolic pathway classes. The predicted metabolic pathway class code corresponds to the code in table 1. Among the 11 predicted pathway classes, the first 2 order predicted metabolic pathway classes should be paid more attention to.

KEGG Ligand	Each order predicted metabolic pathway class										
	1st	2nd	3rd	4th	5th	6th	7th	8th	9th	10th	11th
C03926	2	8	10	7	3	4	6	1	5	9	11
C14600	3	11	2	6	4	8	5	10	9	7	1
C07192	11	10	2	5	4	7	1	6	3	8	9
C05314	5	9	1	10	2	11	8	3	6	4	7
C12271	10	6	3	9	8	2	4	1	11	7	5
C11018	11	10	9	2	6	5	3	4	8	7	1
C14188	11	2	4	10	6	5	8	3	9	7	1
C00027	5	1	8	10	11	6	2	3	4	9	7
C03673	2	7	8	6	5	4	11	3	9	1	10
C10980	11	9	2	10	5	8	3	1	6	7	4
C15560	3	6	8	2	11	10	1	5	9	4	7
C08178	3	5	11	10	7	4	2	1	9	8	6
C10213	11	10	5	8	1	3	7	9	4	6	2
C15415	11	6	9	7	1	10	8	2	3	4	5
C03868	1	10	9	4	5	3	2	8	11	6	7
C14673	11	5	10	8	7	2	3	1	6	9	4
C06843	8	1	5	2	9	3	11	7	6	10	4
C01505	3	5	10	4	9	8	6	7	11	2	1
C09013	11	4	9	8	10	3	1	2	6	7	5
C12091	3	1	5	2	9	4	10	7	6	8	11
C08558	3	10	6	1	5	11	2	8	4	7	9
C13830	10	3	4	1	7	8	6	9	11	2	5
C07919	10	5	2	11	8	7	4	1	3	6	9
C08777	9	5	6	11	2	3	1	8	10	7	4
C03846	6	8	1	5	10	2	9	4	3	7	11
C08050	9	10	11	5	8	2	7	6	3	4	1
C00985	1	5	2	8	3	11	6	10	9	4	7
C01705	5	11	6	2	1	3	10	9	8	7	4
C08438	4	6	11	10	9	2	8	3	7	5	1
C12326	10	2	8	5	6	9	3	11	1	4	7
C12649	1	5	2	11	6	8	3	10	9	4	7
C12849	11	5	1	10	8	4	9	2	3	7	6
C04741	3	10	4	2	5	9	6	8	1	11	7
C07546	1	10	5	11	2	6	9	7	4	3	8
C04132	1	2	5	10	4	6	11	3	9	8	7
C08042	10	9	8	11	2	1	5	4	3	6	7

C14466	11	3	5	8	9	6	7	4	1	2	10
C08699	10	11	5	6	8	1	9	3	2	4	7
C10827	10	9	3	11	5	1	8	6	7	2	4
C00619	3	9	2	10	7	4	8	1	11	6	5
C01945	11	4	2	10	3	1	5	9	8	6	7
C11309	3	10	1	2	4	5	9	11	6	7	8
C05421	9	8	2	3	6	5	11	10	4	7	1
C03070	3	2	8	11	1	10	5	7	9	4	6
C14162	5	1	2	8	6	11	4	9	3	10	7
C12066	9	2	10	4	7	11	8	3	6	1	5
C15664	11	7	5	6	10	9	3	8	4	2	1
C07567	10	5	11	2	3	4	9	1	8	6	7
C08831	10	3	9	2	11	8	1	6	7	5	4
C10121	10	11	4	9	5	3	1	2	8	6	7
C06268	2	6	11	8	1	5	4	7	10	9	3
C08728	5	2	6	4	11	10	8	1	3	9	7
C00661	1	2	5	8	6	9	3	4	11	10	7
C10971	5	6	8	2	3	7	10	1	11	4	9
C02117	2	8	11	6	9	7	3	1	10	4	5
C09644	9	11	3	5	2	7	4	1	8	6	10
C11337	11	10	9	5	4	8	2	3	7	1	6
C07864	10	4	5	8	11	2	3	6	9	7	1
C06430	1	8	10	6	11	4	3	9	5	2	7
C11254	9	10	11	4	6	5	2	1	3	7	8
C14678	11	3	8	4	2	10	5	7	9	1	6
C03995	11	2	10	5	7	6	8	3	4	9	1
C10030	10	3	9	11	5	8	6	1	7	4	2
C13400	11	9	10	5	4	7	3	2	1	6	8
C04500	1	4	7	10	9	6	5	3	2	11	8
C12236	2	10	11	8	4	1	3	9	5	7	6
C14754	10	3	9	5	8	7	4	6	11	2	1
C06453	8	5	2	1	6	11	9	4	3	10	7
C06595	11	1	5	10	9	6	2	3	4	8	7
C09525	5	6	7	11	10	3	9	8	2	1	4
C03093	10	9	1	4	3	11	2	6	8	7	5
C14830	1	6	8	2	7	10	5	9	4	11	3
C10536	10	5	8	7	11	3	4	1	2	6	9
C11356	9	8	2	3	6	5	1	11	7	4	10
C07500	5	11	6	8	1	2	10	4	9	7	3
C07451	5	1	3	9	8	11	4	7	6	2	10
C03963	3	4	9	1	5	10	11	6	2	7	8
C07258	10	5	11	2	3	8	4	6	9	7	1
C14494	11	10	2	5	3	8	7	4	6	1	9

C00732	1	3	2	5	10	9	6	7	11	4	8
C15181	10	3	1	4	2	11	8	6	7	5	9
C01136	1	5	2	6	11	8	3	9	4	10	7
C02441	2	11	5	6	8	1	4	3	10	9	7
C03136	1	5	2	8	10	6	3	11	9	4	7
C03430	10	1	11	2	6	4	5	3	8	9	7
C11344	11	2	3	9	1	5	10	8	6	7	4
C05195	11	5	8	3	1	2	9	10	6	4	7
C11591	1	2	5	6	11	8	4	10	9	3	7
C15439	11	7	2	6	5	10	3	4	9	1	8
C13744	4	10	2	5	3	9	8	1	6	7	11
C03850	2	8	1	4	5	6	10	9	7	3	11
C03665	5	6	1	8	10	2	4	3	7	11	9
C04535	11	5	2	1	4	8	6	10	3	7	9
C08089	2	1	7	3	4	6	5	9	8	11	10
C12058	3	9	5	1	6	11	8	2	7	4	10
C15472	6	11	1	3	5	10	7	8	9	4	2
C00713	1	10	8	6	11	5	3	7	4	2	9
C04638	9	2	4	1	6	10	5	7	3	8	11
C15566	5	10	8	11	3	2	1	4	9	6	7
C09438	10	9	4	6	11	5	3	7	1	8	2
C05317	5	9	10	4	8	3	11	1	6	7	2
C00872	6	5	2	8	11	1	7	9	4	10	3
C15985	2	7	6	8	9	1	10	11	3	4	5
C07970	11	10	9	6	8	5	1	2	7	3	4
C10621	6	5	8	10	1	2	7	3	4	9	11
C14563	11	6	7	3	8	10	1	9	2	5	4
C03209	11	5	8	1	9	2	4	3	6	10	7
C13790	3	8	2	7	9	10	1	5	11	6	4
C07336	11	8	5	6	9	10	3	7	2	1	4
C08080	3	11	5	9	6	4	8	7	1	10	2
C15538	3	9	1	11	6	2	5	8	7	4	10
C07516	5	11	10	1	6	2	4	3	9	7	8
C06942	9	11	6	1	4	3	2	7	5	8	10
C07315	10	9	11	8	5	6	2	3	1	4	7
C01557	10	2	8	5	6	9	3	11	4	7	1
C09266	10	1	2	5	11	6	9	7	3	4	8
C10018	10	11	5	3	2	1	9	8	6	7	4
C14522	11	5	2	9	8	1	4	6	10	3	7
C09135	3	1	10	7	9	6	11	2	4	8	5
C10833	10	11	5	8	1	2	3	9	7	6	4
C12946	5	9	8	3	6	10	1	2	4	7	11
C14671	3	4	6	2	5	1	9	7	11	8	10

C14757	3	11	10	1	5	9	8	7	4	2	6
C09577	10	1	5	7	3	2	8	11	4	9	6
C07911	5	10	9	11	8	2	1	7	3	4	6
C10075	1	5	10	7	9	8	3	4	11	2	6
C02009	5	10	2	11	8	9	7	1	4	6	3
C11335	2	5	8	4	3	6	7	10	1	11	9
C02808	1	4	11	7	8	2	3	10	9	6	5
C07601	10	5	2	1	4	7	6	3	11	9	8
C10146	5	10	9	11	4	6	8	3	1	7	2
C10275	10	1	11	5	8	2	9	6	3	7	4
C14480	4	6	8	11	2	5	9	10	7	1	3
C10450	10	5	11	2	7	3	9	6	8	1	4
C10882	3	10	8	5	1	11	9	6	4	7	2
C14675	3	5	1	2	7	8	10	9	11	4	6
C08713	9	1	4	6	10	3	2	7	5	8	11
C15018	3	9	2	4	6	10	11	5	8	1	7
C10479	1	10	8	9	7	4	6	2	3	5	11
C03797	2	4	9	8	6	5	11	1	10	3	7
C14537	11	1	4	2	7	5	3	9	10	6	8
C07366	3	11	2	10	6	1	5	8	9	7	4
C09209	11	5	10	1	4	3	6	2	8	9	7
C13856	3	6	10	1	8	11	5	2	9	7	4
C08873	9	6	7	5	10	4	3	11	2	8	1
C15722	9	10	11	4	8	7	2	3	6	5	1
C08666	9	11	2	10	4	1	3	6	7	8	5
C08835	9	2	6	7	5	8	10	11	3	4	1
C09775	1	10	9	11	5	8	3	7	2	6	4
C14342	11	1	2	4	5	8	9	7	3	10	6
C12343	10	11	5	3	2	7	1	9	6	8	4
C11487	11	5	1	2	9	7	4	3	10	6	8
C13741	1	2	4	5	8	9	10	3	7	11	6
C13440	10	11	3	2	1	5	4	6	9	7	8
C15502	5	2	1	6	8	11	4	7	9	3	10
C01453	1	10	5	3	8	11	9	4	6	2	7
C03651	5	2	6	8	1	10	11	3	4	9	7
C04350	5	10	1	6	11	9	4	8	3	7	2
C10251	11	2	5	9	1	4	3	10	8	7	6
C02917	1	2	8	6	11	3	5	9	4	10	7
C08321	3	1	6	10	4	7	11	5	8	2	9
C12685	6	1	8	11	5	2	10	9	4	3	7
C08400	10	5	6	7	4	2	1	9	3	11	8
C04634	11	5	7	1	6	9	2	8	4	3	10
C03141	5	8	11	10	3	2	4	6	1	9	7

C09113	3	9	8	6	7	11	10	1	5	2	4
C12008	6	10	7	1	5	2	8	4	9	3	11
C12034	9	7	6	8	11	4	1	3	2	5	10
C12272	3	5	10	8	6	1	11	4	7	2	9
C08463	11	4	6	3	5	10	9	8	7	1	2
C11191	10	5	11	6	3	1	8	2	7	4	9
C03147	3	5	6	8	11	9	1	10	7	4	2
C00946	4	2	8	1	9	10	7	6	11	5	3
C04354	2	8	4	10	5	9	3	1	6	11	7
C00216	1	2	8	5	4	6	11	10	9	7	3
C09785	9	3	5	2	6	8	11	4	7	10	1
C10623	11	9	7	6	1	4	3	10	8	2	5
C11585	10	3	1	5	2	4	11	8	7	6	9
C14217	11	7	4	6	3	8	10	5	1	9	2
C09843	9	11	4	5	8	3	1	2	6	10	7
C14182	5	7	9	8	11	4	3	1	2	6	10
C02518	11	4	5	8	2	10	3	9	1	7	6
C02970	1	2	9	8	7	4	5	6	3	11	10
C12512	3	1	8	11	6	2	5	10	7	4	9
C12679	10	4	1	2	3	9	5	8	6	7	11
C11055	3	1	8	10	4	7	11	9	2	6	5
C14484	11	3	1	4	7	5	8	2	10	6	9
C15718	8	10	6	11	5	9	3	7	4	1	2
C09089	5	10	1	11	6	3	8	9	4	2	7
C06018	1	9	10	4	7	11	3	5	8	2	6
C00796	10	8	11	2	6	5	1	9	3	7	4
C01486	2	8	5	6	11	4	1	9	7	10	3
C09595	10	4	9	11	5	2	7	8	6	3	1
C13810	3	4	9	1	5	8	11	6	2	10	7
C10328	10	2	8	6	9	1	4	11	7	3	5
C00306	10	3	9	2	5	8	4	6	11	7	1
C08158	3	11	5	6	1	8	10	7	4	9	2
C02467	1	10	9	7	4	3	8	5	6	11	2
C11189	9	8	10	11	5	3	4	2	6	1	7
C15389	11	6	7	9	10	5	8	3	1	2	4
C06912	9	10	11	4	6	3	2	7	1	5	8
C11215	1	2	6	8	5	4	11	9	10	7	3
C14488	3	9	7	10	4	2	11	1	8	6	5
C11054	9	6	10	7	2	11	5	3	4	8	1
C15636	3	6	2	10	5	8	1	7	11	4	9
C01604	1	10	5	6	2	11	8	3	7	4	9
C01545	9	11	3	2	5	8	1	10	6	7	4
C10164	5	11	8	10	2	9	1	6	3	4	7

C10482	10	5	7	9	6	8	1	3	4	2	11
C07953	11	10	6	5	3	1	4	2	8	7	9
C02294	5	6	2	10	4	11	9	1	8	7	3
C10076	10	11	4	6	3	2	5	9	1	8	7
C04291	2	6	8	5	11	3	1	10	7	9	4
C08825	3	2	6	8	9	10	4	11	5	1	7
C13858	3	6	4	1	9	8	2	7	10	11	5
C07895	10	11	2	6	7	3	5	4	9	8	1
C13254	5	10	1	7	8	2	3	6	11	9	4
C11043	11	3	10	9	2	8	1	4	5	6	7
C10533	10	3	11	1	8	5	4	9	7	6	2
C09061	9	8	11	10	3	6	4	5	2	7	1
C13664	5	10	11	2	6	1	8	9	3	7	4
C14737	11	9	4	2	1	3	7	10	6	8	5
C09240	10	11	7	1	9	4	3	2	6	8	5
C12338	11	2	1	8	9	10	3	7	5	6	4
C10545	10	3	9	11	5	2	7	4	6	1	8
C04561	2	6	8	7	1	11	3	9	4	10	5
C13980	1	10	5	3	7	6	9	4	2	11	8
C14707	11	8	4	5	10	2	6	7	3	9	1
C01742	1	10	5	8	6	2	9	11	3	7	4
C15761	6	2	4	9	3	11	10	7	5	1	8
C08679	9	2	11	8	4	3	7	6	5	1	10
C07633	9	5	8	10	4	2	6	7	11	1	3
C08415	10	9	5	1	11	6	8	7	3	2	4
C01488	1	2	8	9	4	10	7	6	3	5	11
C10059	9	10	11	1	4	3	5	7	2	8	6
C09800	9	1	10	2	8	11	6	3	7	4	5
C10992	5	11	8	3	4	1	6	7	10	9	2
C07713	10	3	5	2	4	6	1	8	9	11	7
C11490	2	11	6	5	10	7	4	8	3	9	1
C14376	3	10	2	8	5	11	4	9	6	7	1
C07146	5	10	3	2	6	7	9	8	4	11	1
C07673	11	1	5	8	10	2	4	9	6	3	7
C13666	10	5	11	8	1	9	6	3	2	4	7
C11468	8	9	5	1	7	6	11	3	2	4	10
C02942	3	5	10	6	2	11	8	1	9	4	7
C09021	10	5	7	2	8	1	3	9	6	4	11
C10744	3	5	9	10	7	8	11	4	6	1	2
C15678	9	10	11	8	3	1	5	2	6	7	4
C07536	2	8	11	1	6	10	5	4	9	3	7
C08796	3	10	6	9	5	1	4	8	11	2	7
C00767	1	8	2	5	9	11	10	3	6	7	4

C01425	6	9	5	8	11	7	4	2	1	10	3
C04485	2	7	9	1	8	6	5	10	11	3	4
C16216	3	7	9	6	1	5	10	2	11	8	4
C07344	2	8	6	10	9	1	7	5	4	3	11
C08972	1	10	9	4	5	3	2	6	11	8	7
C09921	3	5	2	9	6	4	11	8	7	10	1
C13779	2	1	7	5	4	3	8	9	10	6	11
C13414	3	5	6	2	4	7	9	11	10	8	1
C15655	1	3	8	10	2	11	7	4	5	6	9
C11257	9	10	11	8	6	4	3	1	2	5	7
C06932	10	5	6	7	11	3	9	2	4	1	8
C04025	1	5	2	11	6	8	10	3	4	9	7
C02057	5	1	2	6	8	10	11	4	9	3	7
C02007	9	10	4	6	3	2	11	1	5	8	7
C04830	9	1	4	3	8	10	11	2	7	5	6
C09093	9	4	6	2	3	10	8	5	7	1	11
C09870	9	11	5	10	8	7	3	1	2	4	6
C01677	4	9	11	5	10	1	2	8	7	6	3
C01799	5	6	8	10	2	1	4	7	9	3	11
C03855	1	9	3	10	8	2	4	7	5	11	6
C14451	11	5	8	10	2	1	4	9	7	6	3
C15630	3	8	9	7	6	5	4	11	10	2	1
C07473	10	5	1	4	6	8	2	3	11	7	9
C00505	10	9	1	5	6	2	11	4	7	3	8
C10824	10	5	3	7	2	8	4	9	11	6	1
C15641	10	6	5	3	11	2	9	7	4	8	1
C11764	11	10	6	2	9	5	1	4	3	8	7
C13478	3	4	11	5	10	9	8	7	6	1	2
C14385	11	10	5	8	2	9	4	7	3	1	6
C08392	10	3	8	5	1	9	6	7	4	11	2
C04453	2	6	1	8	4	11	5	10	9	7	3
C02996	3	5	10	11	4	6	9	2	1	7	8
C13716	1	6	5	11	10	2	4	9	3	7	8
C02827	1	2	10	4	8	5	3	6	11	9	7
C12033	5	11	8	9	6	10	7	2	1	3	4
C14761	11	1	5	6	2	8	10	4	3	7	9
C14718	5	11	8	1	3	2	7	9	6	10	4
C08369	10	9	5	11	8	2	3	6	7	1	4
C09561	10	3	1	4	2	5	9	6	11	8	7
C02560	1	11	2	5	8	3	6	9	4	10	7
C14465	11	3	5	4	7	9	10	1	6	8	2
C00348	1	9	7	4	10	3	2	6	8	5	11
C06839	10	9	6	5	1	7	4	8	3	11	2

C07115	11	5	6	2	4	10	3	9	1	8	7
C14640	3	9	11	10	2	7	8	1	5	4	6
C04108	3	9	1	10	6	2	5	11	8	7	4
C14485	3	9	5	4	2	8	1	7	11	6	10
C09651	10	6	9	7	5	1	2	8	3	11	4
C14561	11	2	5	8	7	9	3	4	6	10	1
C14353	11	5	4	7	1	8	3	10	9	2	6
C10090	10	4	11	9	5	7	6	3	2	1	8
C06963	5	6	1	4	11	7	9	3	10	8	2
C03154	3	5	6	11	9	8	1	2	7	10	4
C11091	11	5	9	10	6	2	8	7	4	3	1
C10156	10	11	7	4	1	3	5	9	2	6	8
C14319	10	5	11	8	6	2	1	3	4	7	9
C06356	2	8	5	6	3	1	11	9	4	10	7
C05405	1	2	6	8	10	5	11	9	3	4	7
C10677	10	8	9	1	3	2	7	4	11	6	5
C10288	10	11	9	5	7	8	3	1	4	6	2
C14231	10	6	9	11	5	3	4	7	1	2	8
C01814	5	10	8	11	6	1	2	3	9	4	7
C11624	3	1	11	9	2	8	7	6	5	10	4
C13183	11	6	8	5	2	10	1	9	7	3	4
C08031	9	10	3	5	1	2	8	11	6	7	4
C04886	4	1	6	9	7	8	10	3	2	11	5
C08647	10	1	8	5	9	11	4	3	6	2	7
C10207	10	3	1	5	8	9	2	11	6	4	7
C07177	10	9	1	11	6	5	7	3	4	2	8
C15428	10	3	5	9	11	8	4	6	2	1	7
C12328	10	1	8	2	6	5	9	11	3	4	7
C08555	10	9	8	2	6	3	5	7	1	11	4
C09705	9	11	3	10	2	5	6	1	4	7	8
C08141	3	9	11	2	10	8	5	1	6	7	4
C16438	5	6	2	1	10	8	4	9	11	7	3
C11374	4	2	11	5	6	1	7	8	9	3	10
C11721	4	3	5	9	6	10	11	7	8	2	1
C07334	10	9	1	5	6	11	4	3	7	2	8
C08817	3	9	8	11	6	4	1	5	10	7	2
C06808	5	2	6	11	4	1	8	10	3	7	9
C10804	11	1	8	5	4	9	3	2	10	6	7
C04064	1	10	7	2	9	6	4	3	11	8	5
C11702	11	4	5	10	8	3	6	1	2	9	7
C12122	10	6	5	1	8	9	2	7	3	4	11
C02536	3	7	8	9	2	5	11	6	1	10	4
C12995	1	3	11	4	2	5	9	6	8	10	7

C14526	11	8	2	4	9	6	5	10	1	3	7
C13715	5	10	11	3	8	4	7	9	1	6	2
C13752	2	4	3	7	6	1	11	10	9	5	8
C00586	1	2	10	5	6	8	9	4	3	11	7
C01779	2	8	10	6	9	3	11	5	4	7	1
C02567	6	5	8	2	10	3	11	1	9	4	7
C14172	3	9	8	2	10	6	1	11	4	5	7
C04581	5	9	10	3	1	7	6	8	11	2	4
C10512	1	7	10	11	8	4	3	2	9	6	5
C04486	2	6	8	1	9	7	3	5	4	11	10
C14742	11	2	9	5	3	10	6	7	1	8	4
C09139	10	5	4	6	2	3	9	7	11	8	1
C10956	10	5	2	4	9	6	3	8	7	1	11
C10739	10	1	3	2	8	6	4	11	9	5	7
C11373	1	10	2	4	9	5	8	7	6	3	11
C09754	8	1	6	11	2	4	3	9	7	5	10
C09527	3	9	2	6	5	7	11	8	1	10	4
C08363	3	2	8	6	1	9	10	7	5	4	11
C09708	1	11	8	10	7	3	6	5	4	2	9
C08545	2	10	9	8	5	7	11	1	3	6	4
C03214	5	2	8	1	3	6	11	4	9	10	7
C15991	1	2	10	5	4	7	8	6	9	11	3
C10733	10	3	11	9	6	5	1	8	2	7	4
C00566	2	1	9	5	3	6	4	11	7	8	10
C15261	3	1	5	11	10	7	4	8	9	2	6
C14712	11	2	9	5	3	7	4	6	1	10	8
C14177	4	5	9	2	10	6	3	8	11	7	1
C14701	2	8	11	6	5	1	10	9	3	4	7
C14604	11	2	5	10	1	7	6	3	4	9	8
C11030	11	10	9	2	5	8	7	3	4	6	1
C09652	9	10	4	3	7	6	5	8	1	2	11
C07119	3	9	11	1	2	6	7	8	10	5	4
C11608	3	1	5	7	8	11	2	9	6	10	4
C06502	4	2	1	8	10	9	11	6	3	5	7
C11162	10	8	9	5	6	4	3	1	11	7	2
C08803	10	2	11	9	8	4	6	3	5	7	1
C01275	11	2	8	1	5	9	6	3	7	10	4
C06343	2	8	10	5	11	9	1	3	7	6	4
C11180	11	3	1	10	2	9	8	4	5	7	6
C00611	1	4	2	9	3	10	7	5	11	6	8
C02311	10	1	6	11	8	4	2	5	9	7	3
C07343	5	1	8	2	6	11	9	4	3	10	7
C14343	11	10	9	6	3	8	2	1	4	7	5

C08730	10	11	5	8	2	3	1	7	9	6	4
C12230	10	3	9	2	1	4	7	11	6	5	8
C10254	10	5	3	6	1	8	2	11	9	4	7
C01593	8	2	11	7	9	6	3	5	4	10	1
C12056	5	1	2	4	10	11	9	3	6	8	7
C15631	1	3	9	10	8	4	11	6	7	5	2
C03051	3	2	8	6	5	1	4	10	7	9	11
C02912	1	2	8	6	11	3	5	9	4	10	7
C07866	5	10	8	11	2	7	4	9	3	1	6
C14329	11	9	2	6	5	8	7	3	10	1	4
C00896	10	9	5	11	8	2	6	3	1	4	7
C09182	9	5	10	1	2	6	7	4	3	8	11
C13558	1	2	3	11	5	6	10	8	4	9	7
C03853	3	1	9	10	11	8	2	4	6	5	7
C10101	2	8	3	11	1	9	6	4	10	7	5
C11022	6	7	2	9	1	8	11	10	3	5	4
C06881	10	1	9	6	11	5	4	7	3	2	8
C08350	1	2	11	3	8	9	4	5	10	6	7
C06863	10	11	2	5	1	3	4	6	9	8	7
C09466	9	5	10	11	2	8	4	6	3	7	1
C00308	5	2	6	1	10	8	7	3	9	4	11
C09520	10	3	6	1	9	5	11	2	7	8	4
C09767	10	1	9	3	4	6	8	5	11	7	2
C10255	10	8	5	2	9	11	4	3	1	7	6
C10387	10	11	9	5	6	8	2	3	4	7	1
C07383	10	11	4	7	5	3	2	6	1	8	9
C14272	9	11	1	5	3	2	6	4	7	10	8
C03387	1	8	2	5	6	7	4	9	3	11	10
C15702	10	9	1	11	2	3	8	6	5	7	4
C13962	11	2	1	5	4	9	10	8	6	3	7
C07521	5	11	1	10	3	4	2	6	7	9	8
C11092	9	7	5	4	2	1	8	3	11	6	10
C02201	11	10	1	5	8	2	9	3	4	6	7
C11803	10	5	11	4	3	1	6	2	7	8	9
C10290	1	2	10	5	6	8	7	4	3	11	9
C12020	9	10	4	1	7	5	6	3	11	2	8
C06918	5	10	6	11	2	8	7	3	9	1	4
C10250	10	11	3	2	5	6	8	1	4	9	7
C13089	10	5	1	6	8	4	11	3	7	2	9
C11214	1	11	5	2	6	8	3	10	4	7	9
C08212	3	5	11	1	9	4	6	2	8	10	7
C06384	2	5	4	6	8	11	1	7	9	3	10
C07022	10	5	2	3	4	1	6	9	11	7	8

C08258	1	10	3	4	9	6	5	8	2	11	7
C08483	11	5	9	1	7	10	3	8	4	6	2
C01034	3	5	10	7	1	9	11	6	4	2	8
C06346	10	5	9	11	3	8	2	6	4	1	7
C14001	9	10	6	5	11	8	7	1	3	2	4
C03134	1	2	11	8	3	4	6	5	10	9	7
C06631	9	5	3	10	4	7	11	2	6	8	1
C07425	5	10	3	11	6	1	7	9	8	4	2
C10618	8	11	2	6	10	1	3	7	5	9	4
C09023	9	11	8	7	5	2	3	6	1	4	10
C07311	3	11	7	2	8	6	1	5	9	4	10
C06514	10	5	9	2	1	11	3	4	8	7	6
C07254	3	1	8	2	11	9	10	7	4	6	5
C15765	3	5	1	9	10	6	7	2	4	11	8
C14575	11	1	4	10	2	8	9	6	7	3	5
C06665	10	9	5	1	11	6	3	4	8	7	2
C12639	10	5	6	1	2	7	8	11	3	4	9
C11213	9	10	3	11	4	6	7	8	5	1	2
C10237	10	11	3	6	2	7	5	1	9	8	4
C10759	11	5	9	10	3	4	2	7	1	6	8
C00803	1	11	2	5	3	8	6	9	4	10	7
C10948	11	3	6	9	1	5	2	10	8	4	7
C07241	10	5	11	3	4	8	1	9	2	7	6
C07509	11	10	5	2	1	4	7	9	3	6	8
C08059	9	10	11	8	5	2	7	4	3	1	6
C01602	5	1	6	2	10	8	4	11	9	3	7
C11222	6	9	5	10	11	3	8	4	1	2	7
C15711	1	9	10	5	6	7	3	4	11	2	8
C02253	8	2	5	1	3	9	4	10	6	7	11
C01173	3	11	6	2	4	8	5	9	1	10	7
C12603	1	2	5	11	6	10	4	9	8	3	7
C03401	5	11	8	6	2	4	1	9	10	3	7
C10392	10	11	9	6	4	2	3	1	8	7	5
C03975	8	5	2	10	9	4	11	7	6	3	1
C14248	11	9	1	3	5	4	7	8	10	2	6
C15907	9	1	10	3	8	5	11	2	6	4	7
C02116	2	8	11	1	3	10	6	4	9	5	7
C04720	1	10	5	2	11	8	6	3	7	9	4
C10774	10	2	5	1	9	4	8	6	11	3	7
C11123	8	2	5	9	10	11	6	4	3	7	1
C16347	9	8	11	7	10	4	6	1	3	5	2
C09709	3	9	2	8	5	4	11	10	7	6	1
C11188	11	6	9	4	8	2	7	10	3	1	5

C10027	11	10	4	7	2	3	6	1	8	5	9
C09964	5	11	8	10	9	1	6	4	7	2	3
C13707	11	1	3	6	4	9	5	10	7	8	2
C03985	9	11	10	5	1	2	3	8	6	7	4
C01015	5	2	6	8	1	10	11	3	4	9	7
C01917	10	11	5	9	6	4	2	7	3	1	8
C05343	11	5	8	2	9	1	10	6	7	4	3
C05163	1	10	2	5	8	6	9	3	11	4	7
C15624	3	5	7	6	10	11	4	2	8	9	1
C09667	11	5	9	8	10	1	3	7	4	6	2
C01625	1	2	8	11	6	5	4	7	9	3	10
C14323	11	6	3	5	1	10	9	8	2	7	4
C10726	11	10	3	5	7	8	2	1	6	9	4
C04491	10	5	11	1	9	2	3	8	6	7	4
C09985	2	5	6	4	8	7	1	3	9	11	10
C09351	6	5	4	7	9	3	8	10	11	1	2
C11341	5	11	10	6	1	7	2	4	3	8	9
C07427	10	5	11	8	3	7	1	6	4	2	9
C07665	11	2	10	5	9	4	8	1	7	3	6
C08596	9	8	3	2	1	10	7	11	4	5	6
C07584	10	8	11	7	2	3	6	5	9	1	4
C01537	5	11	6	2	10	1	8	4	3	9	7
C07104	5	10	11	8	9	4	3	6	7	1	2
C08467	10	9	11	6	2	8	1	7	5	3	4
C14267	11	10	8	3	4	9	5	7	1	6	2
C15204	2	5	4	7	9	8	11	1	6	3	10
C07740	10	5	2	9	1	11	4	3	8	7	6
C09544	10	8	4	11	7	3	6	9	5	2	1
C08975	10	9	11	6	4	3	8	5	2	1	7
C06501	11	5	8	2	6	1	10	4	3	9	7
C01171	1	2	4	9	10	8	3	7	6	5	11
C07306	3	5	11	9	2	6	4	7	1	10	8
C10184	10	5	11	1	8	2	9	7	3	4	6
C09837	9	11	10	5	8	2	6	1	7	4	3
C06312	1	2	6	8	9	5	4	10	3	7	11
C13696	5	1	6	10	8	4	2	3	9	11	7
C14448	1	11	5	4	2	3	10	6	9	8	7
C07401	10	11	3	5	2	9	1	4	8	6	7
C04698	1	2	8	9	10	6	7	4	5	3	11
C02494	4	9	10	5	11	6	7	8	3	1	2
C09978	11	6	1	9	2	10	5	4	3	8	7
C11599	10	3	9	8	1	7	5	11	2	4	6
C11735	5	2	8	10	1	11	4	6	7	3	9

C07930	4	8	7	6	5	2	3	1	11	9	10
C09742	10	9	4	2	5	6	11	3	7	8	1
C12078	9	11	3	10	6	5	8	2	1	4	7
C02096	1	2	8	5	4	6	11	10	9	7	3
C06999	5	9	3	4	11	6	2	10	1	7	8
C11098	9	10	2	8	6	5	7	11	3	4	1
C06849	5	10	11	7	4	2	3	9	6	1	8
C00915	2	8	5	1	6	11	3	9	10	4	7
C10698	10	3	1	5	2	7	4	6	11	8	9
C14439	11	2	8	5	1	9	6	7	3	10	4
C06078	9	11	2	8	1	3	6	5	10	7	4
C15357	3	9	4	5	10	7	1	6	11	2	8
C10197	10	2	5	3	8	1	6	4	7	11	9
C14135	2	1	9	8	7	6	11	3	4	10	5
C05230	2	8	6	9	10	7	11	1	4	3	5
C07853	10	11	7	2	1	3	5	6	9	4	8
C16440	5	6	1	2	10	8	4	9	11	3	7
C14397	11	6	3	5	1	10	8	2	9	7	4
C07322	10	5	11	2	3	8	9	6	4	1	7
C11515	5	1	9	11	7	2	4	6	3	8	10
C13823	11	8	1	2	5	9	4	10	3	7	6
C01481	10	5	11	8	3	2	9	6	1	4	7
C09090	9	10	5	3	1	7	4	11	6	2	8
C02432	6	5	2	1	8	7	11	9	10	3	4
C15455	1	5	2	8	6	4	10	3	11	9	7
C02980	11	9	1	7	6	2	8	3	10	5	4
C11626	10	9	11	5	8	3	6	2	4	7	1
C09256	5	10	3	4	11	6	2	1	8	9	7
C09219	10	9	11	1	5	8	2	3	4	7	6
C06693	1	9	5	10	2	11	3	6	4	8	7
C11146	2	8	5	4	6	11	1	9	10	7	3
C15867	9	8	4	5	6	3	1	11	2	7	10
C13745	4	10	2	8	9	5	6	1	3	7	11
C08752	5	4	1	11	10	6	2	8	3	9	7
C15037	2	8	11	6	9	10	4	7	3	5	1
C08738	3	7	10	8	4	1	2	9	11	6	5
C01823	6	5	1	9	3	11	2	10	4	7	8
C14153	8	9	5	10	6	11	3	1	4	2	7
C01379	1	2	9	4	10	3	11	7	8	6	5
C04507	1	4	7	3	9	10	2	11	6	8	5
C11168	5	10	11	8	1	6	4	7	2	3	9
C03088	4	1	6	8	10	3	9	11	2	5	7
C14454	11	5	2	3	8	1	4	6	9	7	10

C07253	11	10	5	1	3	8	7	6	2	9	4
C10305	11	10	5	6	4	8	3	9	7	1	2
C06967	5	10	1	4	6	2	11	9	3	7	8
C11389	9	11	10	5	1	2	3	8	6	7	4
C12021	6	7	8	2	5	9	3	11	4	10	1
C03384	1	2	4	9	10	8	3	7	6	5	11
C01911	1	2	3	5	8	9	6	4	10	11	7
C13861	3	9	2	4	8	6	10	5	11	7	1
C13610	3	2	4	7	11	9	5	1	8	10	6
C13799	10	5	2	11	1	9	3	8	4	6	7
C00070	1	2	8	11	5	6	10	4	9	3	7
C15557	3	4	2	5	11	8	6	7	10	9	1
C01292	6	2	3	8	1	4	10	7	11	9	5
C08757	10	11	2	6	4	5	3	8	7	9	1
C11255	8	2	11	1	5	3	10	6	4	7	9
C13546	10	5	6	2	8	4	11	9	7	3	1
C09155	1	3	6	2	11	4	5	10	8	9	7
C15484	3	6	2	5	1	9	8	10	4	11	7
C08335	6	2	8	11	1	5	3	10	7	9	4
C14497	3	11	10	9	8	5	4	1	6	7	2
C09385	10	11	3	2	9	4	5	7	6	1	8
C14755	1	2	8	11	5	4	6	7	3	9	10
C00873	6	5	11	1	7	10	8	9	3	2	4
C07397	10	11	5	2	7	8	6	9	3	1	4
C08390	2	5	1	3	6	8	11	7	9	4	10
C10335	10	11	7	6	1	9	2	4	5	3	8
C07771	9	11	10	6	5	4	8	3	1	7	2
C09372	5	6	10	8	1	11	3	2	4	9	7
C07471	3	10	5	4	1	2	6	9	8	11	7
C13556	1	3	11	2	4	10	5	7	9	8	6
C08317	2	11	3	5	7	10	8	9	6	1	4
C08380	11	9	3	1	5	2	7	6	10	4	8
C02108	9	11	1	8	7	3	4	10	2	6	5
C12297	9	5	8	6	11	2	3	4	1	7	10
C06856	3	5	10	11	6	2	9	8	1	4	7
C09227	10	9	5	2	11	4	1	8	3	6	7
C02604	1	2	8	5	4	6	11	10	9	7	3
C03736	1	2	4	8	5	6	9	10	7	11	3
C09904	9	10	3	11	8	5	6	1	2	7	4
C10041	10	11	5	8	6	4	9	7	3	1	2
C01327	1	11	5	2	8	6	3	4	10	9	7
C07080	11	10	3	5	8	9	6	7	1	4	2
C09445	10	5	9	11	6	8	3	4	1	2	7

C13698	1	5	2	11	6	10	3	8	9	4	7
C07826	2	3	7	4	9	1	11	5	6	10	8
C04629	2	5	8	1	6	10	11	3	7	9	4
C06971	5	11	1	9	8	10	4	2	6	3	7
C09352	10	7	2	5	8	3	9	1	4	11	6
C07053	10	5	2	7	9	1	3	6	4	8	11
C02499	11	2	5	8	10	6	9	4	3	1	7
C11080	2	9	8	10	5	3	1	7	11	4	6
C13199	2	1	5	4	8	6	7	10	3	11	9
C13422	10	3	6	2	8	5	9	1	11	7	4
C07603	10	6	8	9	2	7	3	5	4	11	1
C08239	1	2	10	5	8	9	6	11	3	7	4
C09405	1	3	5	9	8	4	11	2	10	6	7
C06415	11	1	3	5	4	2	10	9	7	6	8
C03140	6	5	10	2	1	4	11	3	7	9	8
C09765	9	10	1	8	11	4	2	3	6	5	7
C04843	3	11	6	9	2	5	4	1	10	7	8
C01531	3	11	10	5	8	9	1	4	2	7	6
C08347	1	4	9	2	8	10	5	6	3	11	7
C09512	3	8	7	6	9	4	11	10	1	5	2
C10476	5	8	10	11	3	2	6	4	9	1	7
C09975	10	1	8	5	2	6	9	11	4	3	7
C09859	9	11	10	1	8	3	6	5	7	2	4
C15221	11	4	10	1	6	7	3	8	9	2	5
C01784	1	5	2	8	10	6	11	4	7	9	3
C07328	1	11	5	9	2	3	10	6	8	7	4
C01413	1	5	2	8	6	11	10	4	9	3	7
C08275	5	2	6	10	8	9	4	3	7	1	11
C14461	11	1	5	2	4	10	8	7	3	6	9
C02104	9	3	2	8	1	5	6	11	4	7	10
C10405	11	10	3	8	5	7	6	9	1	2	4
C08434	9	4	3	5	10	11	6	8	7	1	2
C07321	10	9	4	11	5	2	6	8	3	7	1
C08696	9	2	1	10	4	8	3	11	5	7	6
C09462	10	5	2	8	7	11	3	4	1	9	6
C06515	2	10	8	6	5	11	9	7	3	4	1
C15205	11	9	5	4	2	7	6	3	1	10	8
C01965	8	9	10	5	2	6	4	11	3	1	7
C11226	11	3	9	10	1	2	7	8	5	6	4
C15513	11	9	2	8	5	3	1	6	10	7	4
C02107	1	2	5	8	11	4	3	10	6	9	7
C04346	6	1	10	9	8	5	2	4	11	3	7
C04580	1	2	3	10	4	5	8	7	9	11	6

C10179	10	11	8	5	3	4	9	6	2	7	1
C10419	10	11	8	6	1	2	3	7	4	9	5
C00878	1	8	2	5	6	7	10	3	11	4	9
C06017	2	6	8	9	10	7	4	11	3	5	1
C09718	9	8	3	6	5	10	7	4	1	11	2
C13734	5	6	8	10	2	1	11	4	3	9	7
C09538	9	8	11	5	10	1	2	3	4	6	7
C02323	1	11	9	5	4	2	10	8	3	7	6
C01675	1	9	3	6	10	5	11	2	8	7	4
C11712	3	7	4	1	8	2	11	10	5	6	9
C12557	10	9	6	5	2	1	3	7	4	11	8
C10199	1	10	4	9	2	3	8	7	6	11	5
C13727	1	4	3	8	10	9	6	2	5	7	11
C07368	5	10	3	1	11	2	4	6	8	9	7
C10998	11	2	8	6	3	5	10	1	4	9	7
C12967	8	3	4	1	10	6	9	11	5	2	7
C12336	11	10	1	4	7	2	5	6	3	9	8
C04622	3	11	8	10	9	5	2	6	4	1	7
C09064	1	2	6	4	5	11	9	3	10	8	7
C13932	1	6	10	5	2	8	11	4	9	7	3
C11278	2	6	8	11	5	4	7	3	1	10	9
C03916	11	3	7	8	5	1	9	4	6	2	10
C07529	10	5	11	1	9	2	4	7	6	3	8
C02777	5	8	10	2	6	1	4	11	3	9	7
C10458	5	10	8	6	1	11	2	3	9	4	7
C13761	3	5	2	1	7	9	11	8	6	4	10
C11346	1	10	11	2	4	3	6	5	7	8	9
C08422	10	5	3	2	11	1	7	8	9	4	6
C15614	2	8	3	5	1	10	7	6	11	4	9
C04674	1	2	8	4	9	3	11	6	7	10	5
C14672	11	5	6	10	1	3	2	9	4	8	7
C07225	9	10	8	6	5	1	11	2	4	3	7
C01383	1	2	8	5	3	7	9	10	6	11	4
C07955	3	9	1	8	11	5	4	7	6	2	10
C13763	10	2	3	1	11	6	7	8	5	9	4
C07541	5	10	9	11	6	8	3	1	7	2	4
C08913	3	4	9	1	5	10	11	2	6	8	7
C14560	11	6	8	2	3	5	7	10	1	4	9
C09476	2	3	10	6	1	7	9	4	8	5	11
C13297	10	9	6	3	8	1	7	2	11	5	4
C02130	1	3	5	11	6	8	2	9	10	7	4
C09097	9	3	4	8	1	11	10	5	6	7	2
C14703	3	11	6	4	9	1	5	7	10	8	2

C04527	5	9	10	3	8	4	11	6	2	7	1
C11172	11	2	8	6	5	3	1	10	7	4	9
C02351	11	5	10	8	2	1	6	9	4	3	7
C08618	10	3	9	11	2	5	8	7	1	6	4
C11777	5	10	11	6	7	9	4	1	3	2	8
C11306	3	9	1	4	5	10	8	6	11	7	2
C06710	2	1	5	8	6	4	10	11	9	7	3
C08833	3	6	1	2	10	7	4	11	9	8	5
C07482	3	5	6	8	4	2	7	11	1	10	9
C13534	10	11	2	6	3	9	4	8	7	1	5
C12329	10	9	5	6	8	7	2	3	1	11	4
C11203	9	11	1	10	3	2	8	6	7	5	4
C07561	11	1	6	5	8	10	2	3	4	7	9
C04299	1	2	10	8	7	3	6	5	11	4	9
C10862	10	1	8	9	2	11	3	6	4	5	7
C09179	10	2	8	6	1	5	9	3	4	11	7
C01757	2	11	5	9	8	1	7	6	10	3	4
C09464	10	1	9	2	5	11	3	7	4	6	8
C11231	9	11	3	10	8	4	2	7	5	1	6
C04058	4	2	9	1	7	6	3	5	10	8	11
C11849	5	7	6	1	8	3	11	4	2	9	10
C07520	5	10	2	11	4	3	6	1	8	9	7
C05650	5	8	10	11	9	6	2	7	3	4	1
C07452	11	10	5	4	2	6	9	1	3	7	8
C10486	10	9	2	8	5	3	11	4	1	6	7
C07714	10	2	3	5	4	6	8	7	9	11	1
C09696	3	6	1	10	7	2	11	4	8	5	9
C11288	3	1	8	10	4	9	6	2	5	11	7
C10300	10	11	5	8	9	1	7	4	3	6	2
C11718	1	8	5	11	2	9	10	4	6	3	7
C13802	3	1	7	10	8	2	11	4	6	5	9
C04110	2	8	9	7	5	3	4	10	6	11	1
C11315	3	11	6	4	9	2	1	8	7	10	5
C13085	1	2	4	7	5	10	8	11	9	3	6
C15554	1	10	3	4	5	2	11	7	9	6	8
C15235	8	11	4	7	9	6	10	3	1	5	2
C10181	10	1	11	6	2	5	9	8	4	7	3
C02395	11	9	10	3	2	6	8	4	5	7	1
C15686	2	5	6	4	1	9	3	11	10	8	7
C12445	9	7	4	6	3	10	11	8	2	5	1
C14194	11	1	2	3	6	4	8	9	5	10	7
C08635	9	4	7	10	6	1	3	2	8	5	11
C11715	1	2	9	8	11	10	5	3	4	7	6

C15336	3	11	1	2	8	4	7	6	5	10	9
C09127	8	10	2	3	4	11	5	6	1	7	9
C13650	10	11	5	8	9	3	2	4	7	1	6
C04543	6	11	8	5	2	10	9	4	3	1	7
C03224	5	11	9	8	6	10	4	1	2	3	7
C01624	3	2	6	1	10	11	7	8	5	4	9
C07706	1	3	5	2	4	8	6	7	9	11	10
C03678	5	1	6	4	11	8	2	10	3	7	9
C10984	9	4	11	2	8	10	5	6	7	3	1
C08754	5	10	11	1	3	4	2	9	8	6	7
C15693	5	10	6	7	1	9	2	11	3	4	8
C13968	10	5	9	6	1	3	11	8	4	7	2
C15201	3	1	8	6	4	5	9	10	7	11	2
C10892	3	5	11	8	10	6	1	2	9	4	7
C08018	10	11	8	2	7	6	9	3	4	1	5
C07538	11	5	10	3	8	2	9	1	6	4	7
C14596	3	11	10	5	6	4	7	1	8	2	9
C11378	8	9	2	5	1	6	11	3	4	10	7
C09649	9	11	5	10	1	4	7	2	3	6	8
C13269	8	9	3	4	2	5	6	11	7	10	1
C15668	5	6	8	2	10	1	3	11	9	4	7
C10469	9	10	5	11	2	6	8	1	7	3	4
C14425	11	2	10	6	9	3	7	5	8	4	1
C08961	11	2	1	9	4	6	3	5	8	10	7
C11505	11	2	1	8	5	3	6	4	10	7	9
C10580	10	4	9	7	8	2	6	3	11	1	5
C14388	11	9	10	2	3	1	7	5	8	6	4
C07568	11	10	5	8	7	3	4	1	6	9	2
C07937	5	6	11	1	10	2	8	9	3	7	4
C15194	3	10	5	2	1	7	11	6	8	4	9
C13630	11	10	3	8	9	2	1	6	4	7	5
C07905	4	11	1	8	6	9	7	10	2	5	3
C09379	8	6	1	4	11	2	10	7	3	5	9
C13718	11	10	1	6	9	2	5	3	4	8	7
C16433	5	1	2	6	8	4	10	11	3	9	7
C14374	3	4	6	10	7	8	11	9	5	2	1
C09688	9	10	6	2	5	11	3	8	7	1	4
C07206	3	5	10	2	7	6	1	4	11	8	9
C10344	10	2	8	1	7	11	3	6	4	9	5
C01554	3	8	2	9	4	10	5	1	11	7	6
C15680	9	5	1	10	4	3	2	7	8	6	11
C10445	10	5	8	11	2	4	9	7	6	3	1
C10224	2	8	6	11	10	4	5	9	3	1	7

C10785	11	5	6	2	9	10	1	3	7	8	4
C08082	11	5	9	3	6	1	8	2	7	4	10
C05000	1	2	9	3	5	8	10	4	6	11	7
C07611	10	2	1	3	9	6	5	11	8	4	7
C14745	10	5	11	7	4	6	1	8	3	2	9
C11101	1	5	2	11	10	8	6	3	9	4	7
C08370	11	10	5	8	6	9	3	1	2	4	7
C07662	11	9	10	6	8	3	1	2	4	7	5
C06470	1	5	6	2	7	3	8	11	10	9	4
C12891	9	7	8	6	10	1	3	2	4	11	5
C10643	10	7	5	3	11	9	6	8	2	1	4
C08869	9	1	2	6	7	3	4	10	11	5	8
C07188	10	3	1	9	5	11	2	4	8	6	7
C13652	1	10	9	8	5	6	3	2	4	11	7
C15637	3	11	9	5	4	7	6	10	1	8	2
C02806	10	5	8	11	9	7	1	2	3	4	6
C11506	11	2	8	1	3	5	4	6	10	7	9
C07239	5	10	3	8	7	9	4	2	11	6	1
C08687	5	9	1	4	2	8	7	3	11	6	10
C08068	5	11	3	10	8	2	1	4	9	6	7
C05153	2	8	11	5	9	10	3	1	7	6	4
C07036	10	5	2	4	3	6	8	11	9	1	7
C10296	3	9	8	1	11	6	2	10	7	4	5
C04489	2	5	9	6	7	8	1	4	10	11	3
C07435	5	10	3	4	11	8	2	9	1	7	6
C07625	1	10	11	9	8	5	3	4	2	6	7
C02636	5	2	8	4	7	11	3	10	6	9	1
C06352	1	10	2	5	8	6	9	3	11	4	7
C02360	2	8	5	4	11	6	1	7	3	9	10
C07362	11	1	4	9	2	3	6	8	10	7	5
C11696	10	5	4	1	9	2	3	7	11	8	6
C08093	6	4	1	9	5	11	3	2	8	10	7
C10056	2	8	6	10	5	4	9	3	11	1	7
C14283	11	5	9	8	10	3	6	7	1	2	4
C07369	3	11	10	4	5	9	2	8	1	6	7
C09596	10	11	8	1	7	5	6	3	4	9	2
C01792	11	3	6	2	10	8	5	9	1	7	4
C11066	11	5	6	4	10	7	3	8	1	2	9
C09154	11	10	9	2	5	1	4	7	6	8	3
C03109	1	2	8	5	9	10	7	6	4	3	11
C06913	5	10	9	1	11	2	3	7	8	4	6
C10709	11	8	4	9	5	10	3	7	6	1	2
C08262	5	1	9	11	10	7	6	3	8	2	4

C13682	10	5	6	8	1	2	3	9	4	11	7
C04613	6	8	1	5	10	2	4	9	3	11	7
C08069	5	11	3	10	8	2	1	4	9	6	7
C10306	9	5	11	4	6	3	8	2	7	1	10
C06814	1	4	2	8	5	9	10	6	7	3	11
C09745	9	10	5	1	11	6	3	2	8	7	4
C10837	1	4	2	7	11	5	10	9	8	6	3
C11825	3	6	2	5	1	9	8	10	4	11	7
C08894	3	2	4	8	11	1	9	10	6	5	7
C06550	2	6	9	1	7	8	4	11	3	10	5
C08982	1	2	10	6	9	3	11	5	4	7	8
C08475	5	1	6	8	10	3	7	4	9	2	11
C10646	10	11	9	5	7	4	8	3	2	6	1
C08621	3	2	8	5	10	1	6	9	11	4	7
C04617	1	2	5	8	6	11	3	4	9	10	7
C10143	1	10	2	4	8	5	7	3	11	6	9
C10552	10	3	1	9	7	6	11	8	5	2	4
C07250	10	9	6	4	5	1	7	8	3	2	11
C11587	3	10	5	1	6	2	4	9	8	11	7
C07498	11	1	10	9	2	5	8	3	6	4	7
C11008	11	2	8	10	3	1	4	9	6	7	5
C10190	10	11	3	5	4	8	1	7	6	9	2
C10110	10	1	11	2	5	9	6	3	8	4	7
C07995	11	1	8	5	2	3	9	10	6	4	7
C08921	1	4	10	5	3	2	11	8	6	9	7
C00962	1	2	10	5	6	8	3	4	11	9	7
C02367	1	6	11	5	2	4	7	3	10	8	9
C07468	1	3	2	5	11	10	9	6	7	8	4
C15600	5	8	10	11	9	6	2	1	3	4	7
C04703	1	3	4	9	10	6	11	5	2	7	8
C14185	11	8	9	10	1	5	6	7	2	3	4
C06536	5	10	6	2	8	1	4	3	7	11	9
C09919	10	11	1	5	8	2	3	4	6	9	7
C08716	10	1	11	5	2	9	8	6	3	4	7
C11232	2	8	11	1	9	5	4	10	6	7	3
C11262	11	4	5	9	10	8	1	2	6	7	3
C11394	9	11	8	6	1	5	3	7	2	4	10
C10135	10	5	11	9	2	3	7	6	8	1	4
C07055	10	5	6	2	7	8	3	1	11	9	4
C09622	9	10	3	1	2	6	8	11	7	5	4
C12100	5	6	2	1	3	9	11	4	7	8	10
C15505	11	2	8	6	5	7	4	1	9	10	3
C15701	4	10	6	8	5	2	1	3	11	9	7

C14130	11	10	5	1	2	9	4	3	6	7	8
C13708	10	4	3	5	2	1	11	8	6	9	7
C02022	1	2	8	6	5	9	3	7	11	10	4
C14390	11	9	8	1	2	5	3	6	10	7	4
C04923	1	7	9	10	4	6	3	8	5	2	11
C08107	4	9	2	10	5	1	11	6	8	3	7
C08898	3	9	10	6	1	2	8	7	5	4	11
C12564	11	10	5	8	3	9	4	7	1	2	6
C05010	1	5	2	11	7	9	3	4	10	8	6
C15703	8	5	3	6	11	1	4	10	9	2	7
C04599	5	10	6	11	8	2	1	9	4	7	3
C10816	2	4	1	10	9	3	8	5	7	11	6
C08527	10	3	9	1	5	11	6	8	7	2	4
C09082	10	5	2	1	8	9	3	11	7	4	6
C09740	9	10	4	1	7	8	2	11	3	5	6
C08794	1	9	10	8	11	3	7	6	5	4	2
C11009	11	10	1	4	2	3	6	5	7	9	8
C14695	2	8	1	5	10	11	4	9	6	3	7
C01387	1	3	11	2	8	5	9	6	4	10	7
C06954	5	10	7	8	3	9	2	6	1	4	11
C06255	5	1	8	2	10	6	11	9	3	4	7
C10261	5	10	3	1	6	9	7	11	2	8	4
C12296	9	11	1	5	6	2	10	3	7	8	4
C10537	10	8	9	5	6	11	1	3	4	2	7
C07506	1	10	4	5	7	2	6	8	3	9	11
C04471	1	2	6	8	9	10	11	5	3	7	4
C06475	3	5	4	2	1	6	10	11	8	9	7
C08439	6	9	11	5	8	4	10	2	1	3	7
C06632	9	1	6	5	2	8	4	3	11	7	10
C06958	10	5	2	3	1	4	8	11	9	6	7
C02484	9	11	5	1	6	8	3	10	7	4	2
C11195	11	8	9	10	4	2	3	6	1	7	5
C10641	8	2	6	11	5	10	7	1	3	4	9
C06527	10	1	2	9	8	5	11	6	4	3	7
C03986	5	11	8	9	10	3	1	2	4	7	6
C09119	2	4	11	5	7	10	1	3	9	6	8
C10468	10	5	1	8	3	11	6	4	2	7	9
C02455	11	5	10	1	6	4	2	3	9	8	7
C10023	10	3	8	5	11	6	2	9	4	1	7
C10886	10	9	1	4	11	8	2	6	5	3	7
C11768	5	3	10	11	8	2	6	7	9	1	4
C02209	1	2	10	5	6	8	3	4	11	9	7
C14395	11	10	9	1	5	7	2	3	6	8	4

C03173	5	2	10	8	7	11	4	1	3	9	6
C10993	5	6	2	8	10	9	11	4	3	1	7
C11791	11	10	5	4	2	7	8	3	9	1	6
C08026	10	11	2	7	3	6	9	8	4	5	1
C12269	5	6	10	8	1	2	3	11	4	9	7
C03235	11	5	8	9	2	3	1	7	10	4	6
C10889	10	11	5	1	2	8	4	6	3	9	7
C05132	5	1	7	4	3	11	8	9	6	10	2
C11077	11	2	8	6	4	9	3	1	5	10	7
C02160	1	10	6	4	7	5	3	9	2	11	8
C07910	8	11	10	9	5	3	4	2	7	1	6
C02028	1	5	2	11	6	8	10	3	4	9	7
C12039	8	9	11	10	1	3	2	5	6	7	4
C07370	3	11	5	10	9	8	4	2	1	6	7
C09706	5	3	1	9	10	4	11	2	7	6	8
C15025	5	3	8	1	2	6	11	9	4	10	7
C13072	1	3	11	10	5	9	6	7	8	4	2
C10620	10	11	6	5	8	3	9	4	7	1	2
C09161	8	1	10	5	2	11	9	6	3	7	4
C10292	10	9	8	11	5	3	7	4	2	6	1
C13694	5	6	1	8	2	10	4	11	7	3	9
C13588	3	4	5	1	11	9	2	7	10	8	6
C13820	4	2	5	10	3	6	9	11	8	1	7
C13730	6	8	5	1	10	2	7	4	9	3	11
C06915	10	9	11	5	3	4	6	2	7	1	8
C07564	10	5	6	1	9	7	3	4	8	11	2
C15504	2	6	8	9	5	10	7	3	1	11	4
C09432	4	11	10	6	2	7	3	1	5	8	9
C10650	11	5	10	9	3	7	4	2	1	6	8
C14021	10	9	5	6	4	2	7	3	11	8	1
C05330	5	2	8	6	1	9	4	3	10	11	7
C04367	2	8	4	5	11	10	9	6	3	7	1
C07230	5	10	11	6	8	7	3	9	4	2	1
C02804	1	5	9	6	3	2	11	7	10	4	8
C08576	10	4	2	6	7	3	11	1	9	5	8
C07752	3	9	2	5	4	7	8	11	1	10	6
C15762	10	8	1	7	9	6	3	5	4	11	2
C09083	10	8	2	5	3	9	7	11	6	4	1
C11366	11	8	6	10	9	5	1	3	4	7	2
C07528	10	5	2	11	8	9	4	1	3	6	7
C09794	10	5	11	1	3	8	9	2	6	7	4
C06422	1	2	3	5	8	9	6	4	10	11	7
C07484	11	5	3	10	1	6	7	4	8	9	2

C02517	3	2	8	6	1	5	10	11	9	7	4
C14407	11	5	9	10	7	8	4	3	6	2	1
C13813	3	2	10	1	11	5	6	8	9	7	4
C01838	3	2	11	8	7	9	5	1	6	10	4
C01893	8	2	5	4	6	11	9	10	1	3	7
C14462	11	5	8	2	1	7	4	3	10	6	9
C07361	9	10	6	5	11	2	8	7	3	1	4
C09312	10	8	4	5	7	1	9	3	11	2	6
C09656	10	9	1	3	7	8	5	11	4	2	6
C09966	9	11	5	10	2	1	4	3	8	6	7
C03601	3	1	11	6	5	2	9	8	4	10	7
C04849	3	11	5	10	8	2	7	1	4	6	9
C08611	9	8	10	11	1	2	4	7	3	6	5
C11041	11	10	6	5	2	1	4	9	3	8	7
C08726	10	1	11	5	8	9	4	6	3	2	7
C11628	1	8	11	5	2	3	9	10	4	6	7
C07331	2	5	6	8	4	11	1	10	3	7	9
C14393	11	3	5	8	10	6	1	9	7	2	4
C16446	11	10	7	2	5	3	8	6	9	1	4
C00623	1	2	3	8	5	6	9	10	4	11	7
C15358	3	5	7	6	8	10	11	4	2	9	1
C08563	10	7	1	11	3	9	2	6	5	4	8
C00904	1	2	5	11	6	8	9	4	3	7	10
C08314	3	1	11	6	4	10	8	2	7	9	5
C09612	10	5	9	3	8	6	11	2	7	1	4
C14538	11	9	10	6	2	3	8	1	7	4	5
C07273	5	2	8	6	11	4	1	3	10	7	9
C11281	10	9	1	11	4	3	6	5	8	2	7
C09570	11	6	7	4	3	10	8	9	2	5	1
C14747	11	5	2	9	8	7	10	6	4	3	1
C07184	10	11	1	8	5	6	2	3	4	9	7
C16261	3	1	6	5	2	4	11	7	8	9	10
C01766	9	1	11	8	2	10	7	6	5	4	3
C07527	11	10	5	9	2	8	1	4	6	3	7
C07346	3	9	4	6	8	5	10	11	7	1	2
C10215	11	10	4	1	2	9	6	7	8	5	3
C15214	11	1	5	10	9	6	2	3	4	7	8
C08780	9	5	1	2	3	8	10	11	7	4	6
C12111	9	3	10	5	11	4	2	8	1	6	7
C08427	10	6	5	11	1	8	2	7	3	9	4
C00561	1	6	11	2	5	9	10	8	4	7	3
C10761	11	9	5	10	2	6	1	4	7	8	3
C10627	3	11	10	5	6	7	4	9	1	8	2

C08633	9	3	1	10	4	6	8	11	2	7	5
C02385	5	2	6	8	10	1	4	3	11	9	7
C03900	6	8	5	1	2	10	11	9	3	7	4
C00604	1	2	11	8	5	6	9	10	3	4	7
C09782	1	10	9	2	8	3	11	4	5	6	7
C01927	10	11	5	1	9	3	6	4	2	7	8
C02611	10	8	3	5	4	1	9	6	2	11	7
C14382	11	9	1	8	7	6	3	2	4	5	10
C13289	10	9	6	11	5	4	8	3	7	2	1
C06330	11	5	10	8	3	7	9	1	6	2	4
C14054	10	5	7	11	3	8	6	4	9	2	1
C09735	11	10	6	8	1	2	4	7	3	5	9
C14663	3	9	5	11	1	8	4	2	7	6	10
C14414	6	10	11	9	2	1	7	5	3	8	4
C11699	3	6	8	9	11	1	4	10	5	2	7
C04204	8	9	11	2	5	10	1	4	3	7	6
C04615	4	6	8	2	5	1	3	9	7	11	10
C10928	11	3	6	5	2	10	1	8	4	7	9
C14558	11	2	4	5	7	3	8	1	9	6	10
C06951	10	8	1	11	4	2	3	6	7	9	5
C08882	5	4	9	1	6	7	10	3	2	11	8
C07766	11	9	2	8	3	10	4	6	1	5	7
C03326	6	2	8	5	7	10	4	9	3	11	1
C12750	8	1	5	10	2	6	4	3	7	9	11
C10755	10	9	11	7	3	2	6	1	4	5	8
C09662	9	2	10	8	6	7	4	11	3	5	1
C05701	5	2	8	6	1	11	9	10	4	3	7
C07879	11	10	2	5	3	4	6	1	9	7	8
C01806	4	10	1	2	5	3	9	6	8	11	7
C02721	5	2	8	6	11	10	1	9	7	3	4
C11923	9	2	6	8	4	1	5	3	10	7	11
C00281	1	2	8	9	7	10	5	4	3	6	11
C10553	10	1	9	3	11	6	8	5	4	2	7
C11501	4	2	8	5	9	11	10	1	3	7	6
C12859	3	9	11	1	2	7	6	10	5	8	4
C14271	9	10	11	1	6	2	4	3	7	5	8
C10289	10	5	2	1	9	3	8	6	7	11	4
C10630	10	9	5	3	1	11	2	6	8	4	7
C08111	10	9	6	5	1	4	7	8	3	2	11
C08655	9	2	10	1	11	6	8	3	7	4	5
C14318	11	10	2	9	7	3	5	4	1	8	6
C12064	11	5	1	8	2	3	6	7	9	4	10
C15508	11	2	8	1	3	5	4	6	10	9	7

C08070	3	5	11	9	10	2	8	1	6	4	7
C06861	5	10	11	3	8	1	6	4	7	2	9
C09545	10	9	11	5	6	8	2	3	1	7	4
C07935	10	5	11	9	3	8	4	6	1	7	2
C09276	2	8	6	10	3	1	5	9	11	4	7
C10036	10	11	5	1	4	7	3	6	2	9	8
C10174	10	3	1	2	9	5	6	11	8	4	7
C09059	9	6	7	1	2	8	3	5	4	10	11
C13515	1	6	2	11	4	9	3	10	8	7	5
C07052	10	5	2	3	4	7	9	8	1	11	6
C07255	3	10	5	4	6	9	8	1	11	2	7
C05394	1	2	8	6	10	9	4	11	3	5	7
C09394	10	9	3	11	1	5	7	4	2	8	6
C00936	1	2	10	5	6	8	3	4	11	9	7
C01969	2	5	8	11	6	7	4	9	1	3	10
C07693	10	5	3	1	4	2	8	9	7	6	11
C08079	3	9	11	5	6	4	1	7	8	2	10
C03666	11	9	2	8	5	1	3	4	6	10	7
C15681	9	2	5	6	1	3	7	4	8	11	10
C06748	2	11	6	8	5	4	10	1	9	7	3
C05537	5	8	6	1	2	11	10	3	7	9	4
C07503	5	11	10	6	8	1	2	4	3	9	7
C11305	5	3	2	1	11	4	10	6	9	8	7
C08090	1	3	9	8	10	4	5	2	11	6	7
C15477	5	10	11	2	1	7	3	8	4	6	9
C10119	10	5	3	9	11	6	7	8	1	2	4
C11493	11	5	1	2	10	8	3	7	9	6	4
C07908	11	4	8	5	3	2	1	9	6	10	7
C14691	11	2	5	6	10	1	3	4	7	9	8
C11053	8	6	10	5	1	2	3	11	4	7	9
C08534	10	4	1	6	3	9	7	5	8	2	11
C05461	3	2	9	8	4	6	5	1	10	11	7
C09671	10	11	8	5	3	9	1	6	4	7	2
C07832	11	5	2	3	9	10	1	7	6	4	8
C00113	1	5	8	2	10	3	11	6	9	7	4
C01729	10	3	2	9	11	6	8	1	5	7	4
C11285	3	4	6	5	7	8	11	1	2	9	10
C09396	11	9	7	1	5	3	8	10	4	2	6
C15473	1	2	5	6	4	11	10	8	3	7	9
C08902	10	9	3	4	7	6	2	1	8	11	5
C01592	10	9	5	6	8	11	4	2	3	1	7
C15605	5	8	2	10	11	1	4	6	3	7	9
C08354	1	2	8	4	6	9	10	11	7	3	5

C03113	2	8	4	1	11	5	9	3	6	7	10
C06878	9	10	6	5	8	7	1	11	3	4	2
C07319	5	3	10	11	2	4	6	1	9	7	8
C09016	1	3	10	6	5	2	9	7	11	4	8
C09092	9	3	11	1	2	5	10	8	6	4	7
C12087	5	8	10	3	6	1	2	9	11	4	7
C03027	2	8	3	6	1	9	7	11	5	4	10
C09898	9	2	8	11	10	3	5	7	1	4	6
C10193	10	5	11	1	3	8	2	4	7	9	6
C11313	4	1	7	11	2	6	3	8	5	10	9
C03002	10	8	6	3	5	9	7	4	1	2	11
C06540	10	2	5	3	7	8	9	11	6	4	1
C08160	3	1	11	2	4	5	9	6	7	10	8
C13240	1	11	3	2	9	10	6	7	8	5	4
C13690	4	2	7	5	10	8	1	6	11	3	9
C16459	11	1	2	6	5	3	7	9	8	10	4
C11514	5	1	9	2	10	6	7	11	3	4	8
C12013	6	10	1	7	9	4	11	3	5	2	8
C09085	10	5	9	7	3	6	2	4	8	11	1
C10420	10	6	5	4	1	3	8	7	9	11	2
C08247	10	1	3	11	6	2	8	5	7	9	4
C07630	8	9	5	10	11	2	4	7	3	1	6
C08619	10	9	5	3	11	8	2	6	1	7	4
C06838	9	10	6	4	11	1	5	7	2	3	8
C14404	11	5	1	2	3	9	4	10	8	6	7
C11640	8	9	11	5	7	6	3	4	10	2	1
C10985	11	1	9	10	2	5	3	6	7	8	4
C07132	10	8	5	11	2	7	3	1	9	4	6
C12009	9	11	10	2	4	3	5	8	6	7	1
C10389	2	10	11	4	8	3	6	9	5	7	1
C10496	11	4	6	2	7	3	8	1	9	5	10
C08287	6	5	8	2	9	1	11	7	3	4	10
C15933	1	9	8	5	2	10	3	7	4	6	11
C10712	11	10	9	5	8	1	7	2	3	6	4
C11609	1	2	6	5	8	9	10	4	11	7	3
C06339	2	8	9	11	5	1	3	6	10	4	7
C06385	2	6	4	5	1	11	9	10	3	7	8
C09398	10	1	3	5	9	7	2	11	8	6	4
C10960	9	11	7	1	5	10	8	2	3	6	4
C01718	5	6	2	8	10	1	11	4	3	7	9
C06313	5	8	10	2	6	4	11	9	3	7	1
C13773	6	5	2	4	10	8	11	1	7	3	9
C14443	11	10	8	2	9	4	5	7	3	6	1

C14173	10	11	5	9	8	1	3	4	2	6	7
C07308	1	2	5	6	8	10	3	11	7	4	9
C06875	3	10	11	4	9	8	6	1	5	7	2
C09626	3	10	1	2	4	11	6	9	7	8	5
C14211	11	8	3	4	1	5	10	2	7	9	6
C08574	10	6	5	4	1	3	8	9	7	11	2
C11200	11	8	7	4	1	9	6	3	2	10	5
C08094	4	9	6	10	11	2	3	5	8	1	7
C07653	3	11	5	1	6	10	4	2	8	7	9
C03207	3	2	8	6	11	1	5	9	10	7	4
C14139	11	5	3	2	8	4	7	1	10	9	6
C03865	6	2	8	1	9	3	7	11	10	5	4
C11639	11	1	9	7	2	4	3	8	5	10	6
C14751	10	3	1	9	11	2	8	6	5	4	7
C07511	10	5	6	8	3	1	11	2	4	9	7
C02024	1	2	11	3	8	9	4	5	10	6	7
C11648	10	9	8	5	11	2	1	6	3	7	4
C12046	3	4	1	2	11	6	7	10	9	5	8
C08021	11	10	5	1	2	4	6	7	3	9	8
C12651	5	10	9	11	8	2	4	7	1	3	6
C11260	3	11	6	5	4	10	8	9	1	2	7
C10393	10	8	5	11	3	6	9	1	7	4	2
C10607	8	9	5	4	2	1	11	3	10	6	7
C11752	11	5	10	7	1	2	3	6	4	8	9
C12574	5	9	10	11	8	1	2	6	4	7	3
C10400	10	5	6	2	11	4	7	3	1	8	9
C14336	11	10	9	3	8	6	2	7	1	4	5
C04716	4	1	9	3	10	6	5	7	2	11	8
C03575	5	9	11	10	4	6	3	7	1	2	8
C14212	3	11	10	1	9	7	4	6	8	2	5
C02810	1	2	8	6	5	10	9	7	3	11	4
C01869	5	10	3	11	9	8	2	7	1	4	6
C04881	1	4	9	10	7	3	6	5	11	2	8
C07934	11	10	2	9	6	3	8	5	1	4	7
C09555	10	11	9	5	6	1	8	2	3	7	4
C07398	1	3	5	2	8	9	10	11	7	6	4
C10417	10	2	8	9	4	11	1	6	3	5	7
C10544	5	10	11	8	7	1	2	9	6	4	3
C13674	6	5	8	2	1	10	11	3	9	4	7
C06924	5	10	11	8	6	2	1	3	9	4	7
C08694	10	5	11	2	1	9	4	3	6	7	8
C14535	11	6	5	2	8	3	10	9	4	1	7
C07641	11	9	10	4	2	8	5	1	3	6	7

C01949	11	1	3	2	8	5	6	10	4	9	7
C06884	5	10	8	4	6	7	2	3	1	11	9
C15734	2	10	7	4	9	5	3	1	8	6	11
C12143	1	9	11	5	2	4	7	10	3	6	8
C15586	4	5	9	11	10	1	2	8	7	6	3
C09842	11	9	8	1	10	2	3	4	7	5	6
C13720	11	3	8	9	2	6	4	10	1	7	5
C11282	10	9	5	11	6	8	1	2	3	7	4
C08471	9	10	6	5	11	3	1	2	8	4	7
C13776	10	2	1	4	5	11	9	6	3	7	8
C10369	11	1	3	10	2	6	5	9	8	7	4
C00652	1	2	8	6	7	10	11	9	3	4	5
C01493	9	1	3	8	2	7	6	10	5	4	11
C11489	2	1	11	8	10	3	6	5	4	9	7
C01517	3	11	10	5	8	2	9	1	4	6	7
C01570	5	6	1	2	8	10	4	3	11	9	7
C09909	9	11	5	2	7	8	10	3	1	6	4
C14473	11	8	9	10	2	1	7	5	3	4	6
C15227	1	5	2	6	8	4	10	3	11	9	7
C07751	10	1	2	5	4	9	3	11	7	6	8
C11246	3	4	10	2	8	11	7	9	6	1	5
C12531	8	11	4	3	9	5	1	2	10	7	6
C09517	5	4	11	6	7	1	10	3	8	9	2
C14380	3	7	11	2	8	9	5	4	6	10	1
C00708	1	2	5	8	6	11	4	3	10	9	7
C08887	1	10	11	9	7	8	6	3	4	2	5
C01835	1	2	9	4	10	3	11	7	8	6	5
C07770	9	10	6	5	11	2	4	1	3	8	7
C11293	8	1	5	11	2	10	3	9	6	4	7
C15919	9	1	8	2	5	7	10	3	11	6	4
C08900	1	10	4	11	2	9	5	3	6	7	8
C09490	5	6	7	2	10	4	11	8	1	3	9
C11166	1	3	5	2	11	6	8	10	9	7	4
C11625	1	3	11	9	2	10	8	7	6	5	4
C09367	10	5	6	7	4	2	9	3	11	1	8
C11120	5	10	2	1	6	3	4	8	11	7	9
C02045	1	2	8	6	5	9	3	7	11	10	4
C04446	6	9	2	5	1	7	8	3	10	11	4
C09162	5	10	3	2	4	11	9	7	1	6	8
C11654	9	2	8	1	11	3	4	5	7	6	10
C08501	11	5	9	1	3	6	7	10	2	8	4
C10453	9	11	10	1	5	3	2	6	8	7	4
C02497	1	2	8	3	5	11	10	9	4	7	6

C14245	3	11	5	2	8	1	6	9	10	7	4
C04284	2	8	1	11	6	5	10	3	4	9	7
C05119	8	5	3	1	4	7	11	2	9	6	10
C01324	1	2	11	8	5	6	4	3	10	9	7
C07628	3	6	5	1	2	11	8	10	9	4	7
C13031	3	9	11	10	2	4	8	1	7	5	6
C08582	9	10	8	11	4	6	5	2	1	3	7
C14294	11	5	8	1	9	2	3	4	7	6	10
C06860	5	2	10	8	11	6	9	4	1	3	7
C02217	2	8	11	6	10	9	5	3	7	4	1
C10403	10	6	5	9	2	7	1	4	3	11	8
C13875	3	1	9	2	5	10	11	7	4	8	6
C10210	10	5	8	11	1	6	2	7	4	3	9
C02297	11	5	6	8	4	1	10	9	3	7	2
C08693	10	9	7	1	4	8	11	3	5	2	6
C09980	10	2	5	4	11	1	3	6	8	7	9
C07247	5	10	4	3	9	2	6	11	7	1	8
C11368	9	10	8	2	6	1	5	3	4	7	11
C05155	8	2	5	1	7	3	10	6	9	4	11
C10427	6	1	9	2	7	8	3	4	10	5	11
C03441	5	2	6	8	1	10	11	3	4	9	7
C09324	10	2	1	9	11	3	6	5	4	7	8
C09598	10	8	11	1	7	5	3	2	4	9	6
C00773	1	5	10	11	3	6	8	2	9	4	7
C11016	9	11	2	8	1	4	6	5	10	7	3
C11208	11	9	1	3	5	7	8	6	2	4	10
C00660	1	2	5	10	3	4	9	8	11	7	6
C01008	2	5	6	8	3	7	10	1	11	9	4
C11770	3	5	10	4	1	2	9	8	11	6	7
C10730	8	2	5	9	11	6	7	3	4	10	1
C13915	3	1	10	4	6	2	11	9	8	5	7
C10857	10	11	2	6	8	9	4	3	7	5	1
C06961	10	5	4	11	3	1	6	9	2	7	8
C13807	3	6	10	5	9	11	2	7	4	8	1
C06847	10	2	3	1	4	5	9	8	7	6	11
C04671	3	8	2	1	10	9	5	11	7	4	6
C07194	5	10	11	4	6	9	8	1	2	3	7
C02652	5	2	8	6	10	11	9	4	3	1	7
C14511	11	1	2	3	7	10	5	6	8	9	4
C12724	3	9	7	1	6	2	8	5	11	10	4
C07764	2	5	11	6	1	4	9	8	10	3	7
C08086	4	2	6	1	7	9	11	10	5	3	8
C02451	5	11	3	1	6	8	2	9	4	7	10

C04700	2	4	5	7	11	1	9	3	8	6	10
C14440	1	2	11	4	8	3	6	5	9	7	10
C04277	8	2	11	6	7	9	1	4	3	5	10
C06975	10	11	5	2	4	7	9	6	3	1	8
C08456	11	5	6	10	7	8	1	3	4	9	2
C16460	11	6	10	5	7	3	4	9	2	1	8
C02479	1	2	8	5	4	6	11	10	9	7	3
C00202	11	2	10	1	6	3	8	5	7	9	4
C11089	11	5	8	1	6	2	10	9	3	7	4
C05328	5	8	10	11	2	7	4	3	1	9	6
C07329	1	11	5	2	9	3	6	10	8	4	7
C03727	5	11	10	1	2	8	6	9	7	4	3
C09590	10	4	9	3	2	6	1	11	8	7	5
C11078	11	9	10	5	8	2	6	7	1	4	3
C09360	10	4	6	7	11	1	3	5	2	9	8
C08237	9	1	8	11	5	7	3	6	4	10	2
C02753	1	2	8	6	7	10	11	3	4	5	9
C06696	1	2	5	3	8	6	9	4	10	7	11
C10272	5	1	3	9	6	10	2	7	11	4	8
C10378	10	11	5	6	7	4	8	3	1	2	9
C08996	11	10	5	2	8	9	1	3	6	4	7
C13377	2	6	1	5	11	8	10	3	4	9	7
C01733	5	2	6	1	8	10	4	9	3	11	7
C07359	11	10	8	2	1	3	4	9	7	5	6
C03764	11	6	5	8	4	1	9	10	2	3	7
C08328	6	7	11	9	10	4	3	5	8	2	1
C10186	10	5	11	2	9	4	3	7	6	8	1
C10689	10	8	2	7	3	6	5	4	9	1	11
C02394	10	9	11	2	5	1	6	8	3	4	7
C03425	3	8	9	5	10	11	1	2	6	4	7
C08665	10	8	4	6	3	2	11	5	7	9	1
C13742	4	1	2	9	11	5	10	3	8	7	6
C00779	2	6	8	5	1	11	9	3	10	4	7
C09531	2	1	7	9	6	10	5	11	8	3	4
C02243	6	2	8	10	11	3	5	9	1	7	4
C09929	10	3	2	9	7	8	6	11	1	5	4
C15224	11	6	1	5	9	10	7	3	4	2	8
C11458	5	11	4	8	9	2	1	3	7	10	6
C07964	5	1	11	2	10	3	8	4	7	6	9
C14127	9	5	8	7	6	1	3	11	4	2	10
C07915	10	1	5	3	2	4	8	11	6	9	7
C14400	11	3	1	7	6	9	4	2	8	5	10
C14326	11	10	9	2	1	3	7	6	8	5	4

C13493	3	5	11	1	6	2	7	8	10	9	4
C09669	10	11	2	5	8	4	7	6	3	1	9
C00918	1	9	5	11	10	6	2	3	8	4	7
C08667	10	9	11	5	6	3	1	8	2	4	7
C16450	11	8	4	3	5	1	2	9	10	6	7
C12299	9	11	5	10	3	2	6	8	1	7	4
C06392	3	11	10	4	5	6	8	2	1	9	7
C07274	10	5	11	8	2	4	6	3	7	1	9
C10875	11	10	6	4	5	3	1	2	7	8	9
C15182	6	4	11	8	5	2	9	3	10	1	7
C14367	11	8	4	2	1	5	7	3	6	9	10
C11336	1	2	6	5	8	10	11	4	9	3	7
C06371	1	2	8	10	7	4	5	11	9	3	6
C09409	3	2	11	1	8	4	7	6	10	9	5
C03860	2	4	6	8	5	10	1	3	11	7	9
C08707	9	3	4	6	8	7	1	2	5	11	10
C15480	1	10	7	2	5	11	8	4	3	9	6
C01716	3	11	1	10	9	2	5	8	7	6	4
C07974	4	10	2	9	5	3	8	1	6	7	11
C00494	10	9	5	6	2	11	1	3	8	7	4
C07663	9	10	11	5	8	4	7	3	1	2	6
C14711	10	2	1	5	6	8	4	11	3	7	9
C15674	8	2	1	5	6	11	9	10	3	4	7
C14502	3	8	2	10	11	5	9	4	7	1	6
C09886	11	5	10	8	3	9	6	1	2	7	4
C11592	2	11	1	6	5	8	4	9	3	10	7
C16015	11	10	5	8	2	7	6	9	3	1	4
C07165	10	11	2	5	9	3	6	4	7	8	1
C08914	3	4	9	1	5	10	11	2	6	8	7
C10452	9	5	11	10	8	4	2	6	1	3	7
C03910	3	5	11	1	2	10	6	7	8	4	9
C09216	10	3	11	2	8	6	4	1	9	7	5
C07219	5	3	1	11	6	10	4	7	9	8	2
C10782	10	9	1	7	8	5	11	3	6	2	4
C15229	11	5	2	10	3	4	9	8	1	6	7
C13197	1	2	5	8	11	6	3	9	10	4	7
C04425	8	2	6	5	9	4	11	7	1	3	10
C08589	9	1	4	10	3	5	8	11	2	6	7
C07811	10	8	9	1	3	6	2	11	5	7	4
C10043	10	1	11	5	8	2	3	9	6	7	4
C07432	5	10	1	9	6	3	8	2	11	7	4
C01017	5	10	8	11	1	2	6	9	3	4	7
C11276	11	10	4	5	9	1	7	2	3	6	8

C12287	9	8	10	2	5	11	4	1	3	6	7
C02635	5	2	6	8	3	1	4	11	10	9	7
C03932	1	2	9	4	10	8	6	11	3	5	7
C06264	1	2	5	11	8	6	3	9	4	7	10
C10455	3	9	1	5	11	2	10	7	8	4	6
C11605	3	8	7	1	10	4	11	2	5	6	9
C01162	2	5	6	9	1	8	7	3	10	11	4
C14167	10	6	8	7	2	4	11	3	9	5	1
C07129	5	10	2	4	11	9	8	6	3	1	7
C01244	6	8	2	1	5	10	4	11	3	7	9
C07743	10	1	2	9	4	5	8	3	6	7	11
C07155	5	10	3	11	1	8	6	4	9	2	7
C05060	11	10	5	1	8	2	3	6	9	4	7
C10640	8	2	7	4	3	10	11	5	6	1	9
C07164	5	10	6	11	8	1	2	9	3	7	4
C07460	5	8	2	4	11	6	1	7	3	9	10
C02825	2	4	5	1	8	11	6	10	7	3	9
C10065	2	8	6	9	11	5	4	3	10	1	7
C14122	11	9	5	10	4	1	7	3	8	6	2
C07545	10	5	3	11	4	2	6	1	7	9	8
C09781	1	10	3	9	11	2	6	4	5	8	7
C11025	11	10	9	2	5	3	1	6	4	8	7
C02256	1	11	10	9	6	7	2	3	4	8	5
C10365	11	3	9	7	6	1	2	5	8	4	10
C01507	1	2	5	8	6	10	3	11	9	4	7
C09321	10	8	11	5	4	2	6	1	3	9	7
C09322	10	11	2	4	9	1	5	7	6	3	8
C12016	7	6	9	2	4	8	1	11	10	3	5
C04109	10	5	9	6	4	8	3	11	7	1	2
C04337	5	10	2	6	8	3	4	9	11	1	7
C07156	5	10	11	3	9	8	6	4	1	7	2
C03394	2	1	8	11	3	9	10	4	6	5	7
C11509	2	8	11	4	1	3	7	9	10	5	6
C14338	11	1	3	4	7	6	8	9	2	10	5
C11856	9	4	11	6	7	5	3	10	2	1	8
C13355	5	10	8	6	3	1	9	4	2	11	7
C14350	11	3	9	4	10	1	5	7	2	6	8
C09675	9	11	4	5	10	7	8	3	2	6	1
C11833	10	6	9	2	5	7	1	8	4	3	11
C11259	6	5	10	4	7	9	2	8	3	11	1
C14579	11	10	5	1	8	6	7	3	2	4	9
C10950	11	10	1	8	3	7	2	9	5	4	6
C09701	4	1	6	11	7	3	10	2	8	9	5

C09611	10	11	4	7	3	2	5	1	6	8	9
C09459	11	7	9	5	10	8	3	4	1	2	6
C06831	3	8	11	4	1	9	5	6	2	7	10
C09730	9	11	3	5	2	10	4	1	6	8	7
C10484	10	5	2	11	6	8	3	4	7	1	9
C03836	3	6	9	4	11	5	1	10	8	2	7
C06923	10	9	11	6	5	7	4	3	1	8	2
C13725	6	4	10	5	2	9	11	7	1	3	8
C07441	5	10	3	2	4	1	11	9	6	8	7
C07517	11	2	1	10	4	6	5	9	3	7	8
C08769	9	10	11	8	7	5	3	1	2	6	4
C14758	3	10	11	8	5	4	9	7	1	6	2
C07624	4	11	6	1	8	3	9	5	2	7	10
C05463	3	6	9	10	8	4	7	2	1	11	5
C07534	3	11	10	5	8	2	9	4	1	6	7
C01582	1	2	10	5	6	8	3	4	11	9	7
C11363	3	8	11	6	2	5	1	10	4	9	7
C06265	1	2	10	11	9	7	4	3	5	6	8
C02964	1	2	3	5	8	9	6	4	10	11	7
C01863	2	8	4	6	10	9	1	3	7	5	11
C10429	9	11	10	5	2	1	8	3	6	7	4
C11193	10	5	3	1	4	9	11	8	2	6	7
C11096	3	6	2	11	1	5	7	4	8	9	10
C03999	6	2	7	9	4	10	8	5	11	1	3
C11312	1	6	10	11	4	8	2	5	7	9	3
C11005	11	6	2	10	8	4	1	7	9	5	3
C05935	5	6	11	10	9	1	2	8	4	7	3
C07418	5	6	2	8	3	11	1	10	9	4	7
C13717	1	5	7	4	9	6	3	8	10	11	2
C13701	6	3	11	10	4	2	9	5	1	7	8
C08397	3	11	4	10	7	2	1	5	8	9	6
C03215	2	1	8	7	10	5	6	3	9	11	4
C02143	1	2	8	5	3	6	10	7	4	11	9
C06573	1	10	3	9	5	11	2	7	4	6	8
C12668	10	1	4	9	8	11	3	2	7	5	6
C02558	11	3	5	8	10	1	6	2	9	7	4
C10714	1	8	11	7	9	6	3	4	5	2	10
C07594	9	3	1	5	10	7	8	11	4	2	6
C15687	11	5	1	9	2	8	6	7	3	10	4
C10058	10	9	11	5	1	3	6	2	4	7	8
C12871	2	4	5	1	3	11	6	7	8	10	9
C08435	9	4	1	10	3	6	7	8	5	2	11
C07168	10	2	4	3	9	8	1	11	5	6	7

C09952	10	5	2	7	9	4	3	1	8	11	6
C10475	9	10	5	6	4	7	3	2	8	1	11
C15587	4	2	10	9	5	11	8	1	7	6	3
C06466	1	2	10	5	6	8	3	4	11	9	7
C07871	11	10	5	7	4	2	3	1	6	8	9
C09189	1	10	9	8	5	6	11	3	2	7	4
C08303	5	10	2	3	9	4	8	11	7	6	1
C06393	5	11	8	6	2	4	1	9	10	3	7
C06725	8	2	7	6	3	4	5	10	9	11	1
C09446	10	8	5	11	6	9	1	4	3	7	2
C01913	2	8	1	11	5	6	10	4	9	7	3
C09670	10	2	11	4	9	7	5	6	3	1	8
C06976	2	10	11	8	5	3	6	4	7	9	1
C00273	1	2	6	8	3	10	4	5	9	11	7
C14517	11	10	8	7	6	1	9	4	3	2	5
C09160	5	9	11	10	7	2	3	1	8	4	6
C06086	9	2	7	11	1	10	3	6	4	8	5
C09234	10	2	6	1	3	5	7	8	4	11	9
C11013	11	8	7	5	3	2	10	1	6	4	9
C01607	3	1	8	5	9	2	10	11	6	7	4
C09058	10	11	4	2	7	3	8	5	1	9	6
C11125	10	11	7	1	3	2	9	4	6	5	8
C05001	1	11	2	3	8	10	9	4	5	7	6
C08624	3	9	2	6	8	1	7	5	11	4	10
C05849	8	2	11	10	5	6	9	1	7	3	4
C07532	5	11	10	9	2	8	4	3	1	6	7
C08102	10	11	4	9	1	3	2	6	5	7	8
C12011	9	5	8	7	4	2	11	3	1	10	6
C08746	10	3	4	8	7	5	6	11	9	1	2
C02927	3	8	1	2	5	4	11	9	10	7	6
C10386	10	9	6	5	7	3	1	2	4	11	8
C01870	6	2	8	11	1	9	10	4	3	7	5
C14286	11	1	3	8	5	10	2	7	9	6	4
C02206	10	9	11	4	8	7	1	3	5	6	2
C07707	6	10	5	2	8	4	7	3	9	1	11
C07997	5	6	10	8	9	1	3	11	4	2	7
C16452	11	10	2	8	5	1	3	9	7	6	4
C14533	11	5	1	9	2	8	7	10	3	6	4
C03416	9	2	6	1	7	10	11	8	3	5	4
C10351	8	9	7	6	1	2	11	3	10	4	5
C13569	9	6	10	4	11	8	5	1	2	7	3
C14190	3	11	10	6	8	7	2	9	1	5	4
C03091	3	6	2	9	8	5	1	10	11	7	4

C08951	11	1	7	5	8	9	3	2	10	4	6
C13908	1	7	3	2	10	5	11	6	9	8	4
C09875	9	11	5	10	8	7	1	4	3	6	2
C11015	11	5	1	2	6	10	4	8	9	3	7
C03352	11	10	5	1	3	4	8	6	2	7	9
C09152	5	10	11	7	4	2	3	8	1	9	6
C07278	1	2	5	3	7	6	8	10	9	4	11
C14588	3	9	1	2	11	4	7	6	8	5	10
C14475	3	10	2	8	4	9	1	7	11	6	5
C00771	1	10	5	2	6	11	8	3	7	4	9
C14423	11	4	5	2	1	9	6	3	10	8	7
C12075	10	4	7	5	11	6	3	2	9	8	1
C15626	3	11	8	5	1	10	2	4	6	7	9
C12286	9	10	11	8	3	7	2	6	5	4	1
C09687	3	8	10	1	9	11	2	7	5	4	6
C11644	1	10	11	6	7	5	4	3	9	8	2
C06269	2	6	11	8	5	1	4	3	7	9	10
C06896	10	9	5	11	8	4	6	3	1	7	2
C01576	10	9	11	8	4	2	6	5	1	3	7
C10499	5	10	9	7	1	11	3	2	6	4	8
C08250	1	10	3	6	11	2	4	9	5	8	7
C11800	5	8	6	9	4	10	3	2	11	1	7
C04126	5	2	8	1	4	6	11	9	3	10	7
C07626	5	10	9	1	11	8	4	6	3	2	7
C07761	10	1	9	2	5	7	6	8	11	3	4
C12888	3	5	11	4	10	6	1	9	2	7	8
C07294	11	8	6	2	9	10	7	5	3	1	4
C03246	1	7	8	10	9	4	3	2	6	11	5
C01485	2	1	8	6	4	5	7	11	9	10	3
C08569	10	5	8	11	6	1	2	7	4	3	9
C13703	1	11	2	5	6	9	7	4	10	8	3
C02095	1	2	5	10	3	6	8	9	4	11	7
C09755	10	1	5	11	6	3	2	9	8	4	7
C08404	10	5	2	1	4	9	6	11	3	8	7
C14332	11	6	8	2	10	7	3	9	1	4	5
C00766	10	5	8	11	1	6	3	9	2	4	7
C10415	2	6	10	8	9	7	11	3	1	5	4
C14744	11	1	5	8	2	3	6	4	7	9	10
C12027	7	9	1	6	2	8	11	4	10	3	5
C01619	10	11	3	5	4	6	1	2	7	9	8
C01776	1	3	9	4	5	7	11	2	10	6	8
C11271	11	9	1	3	8	2	10	5	6	4	7
C03037	8	10	2	3	6	4	11	7	9	5	1

C16256	10	5	11	1	3	9	6	8	2	7	4
C15632	3	9	7	5	6	10	2	11	4	1	8
C02965	1	2	5	8	4	6	3	9	10	7	11
C04138	2	6	10	5	9	8	4	1	7	3	11
C02752	5	11	1	10	3	9	8	6	2	7	4
C14402	11	5	1	10	6	2	8	4	7	9	3
C09715	3	10	8	2	9	5	11	4	6	7	1
C14250	11	5	6	10	3	8	4	2	7	1	9
C10871	10	9	8	2	6	11	3	1	5	4	7
C08329	1	6	10	4	5	7	9	11	3	2	8
C07246	5	10	11	4	8	3	2	7	9	1	6
C08549	10	8	5	9	2	11	3	1	6	7	4
C10926	11	6	5	10	8	1	3	4	9	2	7
C04794	2	5	4	8	9	1	6	3	11	7	10
C10868	10	2	9	5	6	7	4	1	11	3	8
C07549	2	5	9	6	1	4	10	3	8	7	11
C06874	10	5	1	3	2	4	6	7	11	8	9
C08950	9	4	1	3	10	8	2	11	7	6	5
C03748	3	8	7	9	5	1	11	6	2	4	10
C10787	11	10	5	2	8	9	4	6	7	3	1
C08374	11	8	9	2	3	7	1	5	10	6	4
C12080	1	9	3	11	2	6	5	7	4	8	10
C07430	5	11	10	6	1	7	8	9	4	2	3
C06812	9	10	1	6	5	8	7	11	4	3	2
C08088	6	11	1	5	4	9	3	8	2	10	7
C08067	6	5	8	9	11	2	1	10	3	4	7
C14572	11	5	9	2	4	1	6	7	3	8	10
C02944	3	7	2	10	8	5	1	11	4	6	9
C09734	10	11	5	8	1	7	6	9	3	2	4
C14455	11	2	8	5	9	7	4	3	6	1	10
C02587	8	6	5	4	7	11	10	1	3	2	9
C07280	9	11	10	5	8	6	4	1	3	7	2
C13732	1	6	5	10	8	2	3	11	9	7	4
C13503	1	8	6	2	11	10	5	3	4	9	7
C01936	1	2	8	9	10	11	7	6	5	3	4
C07709	1	5	3	11	4	6	9	8	2	7	10
C05765	2	6	4	8	10	9	5	1	3	11	7
C14126	5	6	8	10	1	2	11	7	9	4	3
C12083	3	1	8	5	9	2	11	4	10	7	6
C11739	5	10	11	6	4	7	8	3	9	2	1
C14301	11	9	10	7	8	6	4	3	2	5	1
C12153	10	9	5	1	3	11	6	8	7	2	4
C04084	2	8	1	9	6	4	7	10	3	11	5

C16293	10	5	11	4	1	7	9	3	6	8	2
C14215	11	8	1	5	2	9	4	6	10	7	3
C12609	10	1	9	7	6	4	11	5	2	8	3
C00919	2	5	4	7	8	3	6	10	1	9	11
C14741	11	10	8	6	5	2	3	7	1	4	9
C02807	11	2	6	8	4	5	1	3	10	7	9
C08019	10	11	1	5	4	9	8	7	3	6	2
C09803	10	1	3	4	8	5	2	9	11	7	6
C02793	8	10	2	1	3	6	5	7	11	4	9
C08022	10	11	5	1	2	3	4	6	7	8	9
C07266	10	3	5	1	2	6	4	9	11	8	7
C02932	9	2	11	8	6	5	10	4	3	1	7
C13667	5	6	8	10	11	1	2	3	9	7	4
C10373	11	8	9	1	4	10	3	7	5	2	6
C02952	2	8	9	4	10	3	5	11	6	1	7
C09959	10	1	5	11	9	2	3	6	4	7	8
C06476	3	11	4	7	10	1	8	5	2	9	6
C09128	9	8	10	3	6	1	7	11	5	2	4
C10881	1	9	10	11	5	2	7	8	3	6	4
C13797	1	6	5	4	8	10	9	7	2	3	11
C11733	1	10	2	3	5	7	8	4	6	11	9
C12242	10	5	11	1	9	7	3	4	6	8	2
C15007	4	2	7	3	10	9	1	11	6	8	5
C01526	2	5	8	6	4	10	1	3	9	11	7
C13815	2	11	4	6	5	8	10	1	7	3	9
C02886	5	3	1	6	4	2	11	10	9	8	7
C11284	5	9	1	10	2	3	11	4	8	7	6
C02180	11	5	2	4	8	7	10	6	3	9	1
C14649	3	11	10	4	9	1	8	5	6	7	2
C10414	10	3	1	5	9	6	8	11	2	4	7
C00495	8	2	11	5	3	7	10	1	9	6	4
C09072	8	5	4	10	2	11	3	6	1	9	7
C13102	9	2	5	11	4	10	8	3	6	1	7
C09427	10	9	4	2	5	6	1	7	3	8	11
C12316	10	8	2	5	6	1	11	4	3	9	7
C00869	2	8	1	5	11	4	6	9	3	10	7
C01933	5	6	10	1	8	2	4	7	9	3	11
C08440	6	5	2	4	3	7	10	11	1	9	8
C08879	5	11	3	10	6	4	8	1	7	2	9
C01971	1	2	3	5	8	9	6	4	10	11	7
C10411	10	8	4	7	6	1	9	5	3	11	2
C03542	2	8	11	5	6	9	4	7	1	3	10
C07670	1	5	11	10	4	2	9	8	7	3	6

C11546	2	8	7	1	6	3	5	9	11	4	10
C01489	1	2	5	8	6	10	3	11	9	4	7
C02181	11	5	10	6	1	7	9	2	8	3	4
C04547	2	8	11	5	1	9	6	3	10	4	7
C14559	11	4	9	6	1	5	7	3	2	10	8
C13731	6	1	8	5	11	10	2	9	3	4	7
C07159	2	8	9	6	4	10	3	1	5	11	7
C10425	10	11	1	9	8	5	4	2	7	3	6
C07149	1	9	10	5	11	3	8	7	2	6	4
C06372	1	2	5	3	7	8	6	10	9	4	11
C14412	11	5	2	1	6	10	4	7	3	8	9
C12525	3	2	8	6	5	1	4	10	7	11	9
C01458	5	9	8	10	6	11	3	2	7	1	4
C06865	3	1	11	4	7	2	9	5	6	8	10
C12300	9	11	5	3	4	2	1	6	10	8	7
C14247	11	5	9	8	3	10	7	2	4	1	6
C04540	1	2	5	6	8	11	10	3	9	7	4
C08195	3	1	5	10	11	2	4	9	8	7	6
C12675	3	2	8	10	6	4	11	9	7	5	1
C09702	9	10	3	11	7	5	2	1	4	8	6
C06537	11	4	1	5	2	8	10	7	9	6	3
C04081	11	5	8	2	4	6	3	1	9	7	10
C11178	6	11	10	8	2	5	1	9	7	3	4
C04274	11	1	5	6	8	10	9	2	7	3	4
C06784	1	6	5	2	8	7	10	3	9	4	11
C04834	2	8	10	11	9	5	3	7	6	4	1
C03964	5	8	10	2	9	4	1	11	3	6	7
C02168	3	11	10	5	9	1	7	2	4	8	6
C01014	11	2	7	9	5	4	3	8	1	10	6
C09215	10	5	9	8	4	3	11	6	2	1	7
C07760	5	1	6	11	9	3	7	2	8	10	4
C10559	10	3	9	1	5	2	11	7	4	8	6
C03776	1	8	3	10	2	5	11	4	6	9	7
C01631	10	9	11	2	1	4	6	3	7	8	5
C05416	3	9	5	4	2	8	11	10	1	6	7
C07288	9	2	11	8	5	3	6	10	4	1	7
C00324	2	8	1	11	5	6	10	4	9	7	3
C08587	9	8	3	6	7	4	1	11	10	5	2
C07296	3	5	1	6	8	9	10	7	4	11	2
C08490	9	3	11	10	5	8	2	1	6	7	4
C11707	5	10	8	1	2	9	4	3	6	11	7
C11652	1	9	3	10	6	4	11	8	2	7	5
C03703	4	8	5	2	11	7	3	9	10	6	1

C11600	2	6	1	7	3	5	9	10	4	8	11
C13963	9	3	1	8	7	2	5	6	10	11	4
C10664	11	10	5	1	6	8	9	3	7	2	4
C12284	1	5	10	11	8	6	3	2	9	7	4
C04404	2	8	11	4	7	5	10	3	9	6	1
C11775	5	10	9	6	8	3	2	1	11	4	7
C01536	5	6	8	10	2	1	11	4	9	3	7
C06153	1	8	3	2	5	4	10	9	6	11	7
C08387	10	3	1	2	9	4	5	8	6	11	7
C14715	11	5	1	3	2	10	4	7	6	8	9
C07469	1	3	5	4	10	2	6	7	9	11	8
C15019	10	8	6	5	4	1	3	11	7	2	9
C15403	3	8	4	5	1	7	11	9	6	2	10
C11369	1	10	2	6	8	9	5	4	3	11	7
C03716	2	1	8	5	11	10	9	3	7	6	4
C09619	10	9	7	5	1	4	3	8	6	2	11
C07634	9	11	5	10	2	1	6	7	8	3	4
C08291	6	5	2	8	1	10	9	4	3	11	7
C02060	5	2	8	1	9	4	6	3	11	10	7
C11792	11	10	5	6	9	2	8	7	3	1	4
C04399	2	7	8	9	6	11	10	5	3	4	1
C03738	6	5	2	4	8	7	1	9	10	3	11
C01700	3	1	10	5	6	8	7	11	4	9	2
C09098	10	5	3	9	1	11	4	8	6	2	7
C14155	9	8	3	5	2	10	1	4	11	6	7
C03264	5	10	3	8	1	9	2	4	7	6	11
C07070	11	10	9	5	4	8	2	3	1	6	7
C15417	3	5	6	1	2	4	7	11	10	8	9
C06950	10	9	5	11	2	8	3	1	6	4	7
C08344	6	11	2	5	4	8	10	7	3	1	9
C13403	5	11	7	3	9	1	10	6	2	8	4
C10934	11	2	1	5	9	8	7	6	4	10	3
C15291	3	1	11	8	4	9	5	2	6	7	10
C11332	5	10	6	2	8	1	4	11	3	7	9
C12307	9	11	1	5	3	8	2	6	10	7	4
C01719	1	2	8	9	4	10	6	5	7	3	11
C12554	6	1	5	2	8	11	10	9	7	4	3
C10176	10	11	1	5	2	9	6	3	4	8	7
C14386	11	9	5	8	7	1	4	2	3	10	6
C11511	5	2	6	8	4	1	11	10	3	9	7
C08760	10	9	6	1	4	5	3	7	11	8	2
C02724	5	9	8	6	2	4	10	11	7	3	1
C16455	5	11	7	4	9	3	1	10	6	8	2

C10118	10	11	5	9	8	7	2	4	1	3	6
C10160	10	8	3	1	2	7	6	9	11	5	4
C13692	5	10	6	11	8	1	3	2	4	7	9
C07456	10	2	3	5	6	4	9	8	1	7	11
C10844	10	8	11	1	2	3	5	4	6	9	7
C11326	5	3	11	1	9	2	7	6	4	8	10
C06886	10	9	8	6	5	11	3	7	1	2	4
C08384	10	3	9	11	5	1	8	2	4	6	7
C12036	7	6	2	11	10	9	4	8	3	1	5
C01712	3	1	2	8	5	9	6	11	10	4	7
C12278	1	3	11	10	5	6	9	8	4	7	2
C10991	11	6	10	9	1	2	5	3	4	7	8
C07141	10	3	5	1	8	6	9	2	4	11	7
C12691	10	9	8	1	2	6	3	7	11	5	4
C01755	1	2	5	6	11	8	10	3	4	9	7
C15553	3	5	10	2	1	4	7	11	8	9	6
C13426	3	11	5	8	6	4	7	2	1	9	10
C04441	5	9	10	1	7	3	8	2	11	4	6
C07654	9	1	10	11	6	8	4	7	5	3	2
C07763	11	8	7	4	3	1	2	10	5	9	6
C08241	1	10	7	3	9	2	5	8	6	4	11
C13750	10	2	5	9	11	1	4	6	3	7	8
C01464	1	11	9	2	10	8	6	7	3	5	4
C08964	1	4	9	3	5	6	7	10	2	8	11
C14445	11	10	6	9	7	8	3	2	5	4	1
C14348	11	3	5	2	4	6	8	10	1	9	7
C10333	10	1	11	7	4	3	9	2	5	8	6
C15412	3	4	5	10	8	9	6	1	11	7	2
C12490	6	5	8	9	11	1	2	10	3	4	7
C10898	8	3	2	5	7	6	11	4	10	1	9
C14433	11	10	2	9	4	6	1	7	5	3	8
C12308	9	8	5	1	6	11	2	4	3	7	10
C06617	9	10	6	1	11	4	3	7	2	5	8
C08348	1	2	11	3	8	9	4	5	10	6	7
C08897	3	10	2	1	9	7	6	5	11	4	8
C00282	5	1	10	11	8	9	3	2	4	6	7
C14358	11	4	5	2	8	7	3	9	6	10	1
C11063	11	8	10	5	3	6	9	2	4	7	1
C08115	10	9	11	8	2	4	3	6	1	7	5
C07933	10	1	5	6	2	4	8	11	9	3	7
C08260	1	2	8	7	5	6	4	11	9	3	10
C00703	1	2	5	9	8	6	11	10	4	3	7
C13737	5	6	10	1	8	2	11	3	4	9	7

C09719	9	5	11	10	6	2	7	3	8	4	1
C08267	5	6	10	1	8	2	9	4	11	3	7
C10166	10	3	2	7	6	9	8	11	5	4	1
C07622	11	3	1	9	5	10	4	8	6	7	2
C14656	4	6	5	8	10	9	3	2	1	11	7
C10448	1	9	4	10	3	11	5	2	8	6	7
C07486	11	6	8	10	2	5	1	3	7	4	9
C01826	5	6	8	10	2	1	4	7	9	3	11
C01876	3	11	9	6	10	7	5	1	2	4	8
C02289	9	2	8	6	11	10	7	3	4	5	1
C03368	10	9	5	7	3	8	1	2	4	6	11
C15274	1	5	10	7	8	4	3	2	6	11	9
C09137	9	4	5	8	6	3	2	11	10	7	1
C09165	10	6	8	4	11	7	1	3	9	2	5
C14253	1	11	10	3	9	7	6	4	5	2	8
C07174	10	5	1	6	2	8	3	9	11	4	7
C10111	10	9	7	11	5	8	6	1	3	4	2
C14507	11	5	2	6	9	7	3	4	8	10	1
C07887	10	5	11	1	2	9	4	7	6	3	8
C09664	9	11	10	3	6	2	7	8	1	4	5
C12915	10	5	11	4	6	2	3	9	1	7	8
C00941	4	2	9	10	7	8	3	6	1	11	5
C09147	10	11	9	5	6	3	2	1	8	7	4
C05148	11	5	8	10	3	6	9	4	1	2	7
C00770	1	2	8	5	6	11	9	4	3	10	7
C02299	11	2	7	6	5	3	4	10	9	1	8
C11371	3	11	6	5	8	7	10	4	1	9	2
C15928	9	1	8	7	2	6	3	11	10	5	4
C14417	11	10	5	3	1	8	4	9	2	7	6
C00291	1	2	5	8	6	11	4	3	10	9	7
C05031	4	1	11	2	3	7	6	8	9	5	10
C06949	1	10	5	2	6	4	8	3	9	11	7
C02615	1	6	11	2	5	9	10	8	4	3	7
C08859	10	5	2	1	8	6	9	4	11	7	3
C07034	5	10	2	1	3	11	7	6	9	4	8
C07218	5	10	4	11	6	2	3	1	8	9	7
C01588	1	3	2	10	5	8	11	9	4	7	6
C10038	10	11	5	2	6	9	7	8	3	1	4
C07408	5	10	11	9	4	6	2	8	3	1	7
C00961	9	11	4	2	10	7	1	5	8	3	6
C07270	10	5	3	8	7	1	4	6	11	2	9
C08692	9	11	7	5	10	4	3	8	2	1	6
C10330	10	1	9	8	2	11	4	6	3	7	5

C14146	9	8	4	5	1	11	3	10	7	2	6
C09682	9	10	8	4	11	1	6	3	2	5	7
C08448	11	5	10	8	2	9	6	3	1	7	4
C10214	11	10	1	9	4	6	7	3	2	8	5
C10558	11	10	5	3	2	4	6	8	7	1	9
C12270	5	10	6	1	2	8	3	11	9	4	7
C01432	1	5	2	6	8	3	11	4	10	9	7
C12243	10	2	11	9	6	7	1	3	8	5	4
C06978	10	11	1	8	4	2	5	3	6	7	9
C06996	5	10	3	11	2	8	7	1	6	4	9
C08917	1	10	11	3	2	6	8	9	5	4	7
C10426	11	2	5	10	6	7	1	9	3	8	4
C04865	2	8	5	4	1	10	7	6	3	11	9
C09698	9	10	11	3	7	4	2	1	5	6	8
C14498	3	2	5	4	8	1	6	11	10	7	9
C08533	10	11	9	2	4	1	8	7	3	6	5
C14320	11	10	2	9	8	1	3	5	7	6	4
C06369	1	10	2	8	5	6	9	11	7	4	3
C08378	8	5	10	3	11	4	6	1	7	9	2
C09141	10	2	3	9	5	6	11	8	1	7	4
C03626	5	2	6	8	3	11	4	9	10	1	7
C08373	11	5	8	7	4	1	2	9	3	6	10
C12154	10	1	9	3	11	8	7	5	2	4	6
C12222	10	11	4	1	2	8	3	7	6	9	5
C02961	4	1	2	9	10	8	11	5	6	7	3
C06468	1	2	5	6	8	3	10	11	9	4	7
C02205	1	2	8	5	4	6	11	10	9	7	3
C02466	2	1	4	7	5	11	8	3	10	6	9
C11294	1	10	11	9	4	8	6	2	3	5	7
C11031	4	11	1	10	2	3	5	8	9	6	7
C08668	9	6	1	3	2	10	11	7	5	4	8
C01048	1	9	7	4	10	2	8	3	5	11	6
C04798	2	8	5	7	4	9	10	3	11	1	6
C14435	11	4	10	2	6	5	7	8	3	9	1
C10240	11	4	10	5	1	3	6	7	2	8	9
C02135	3	5	1	2	6	10	4	8	9	11	7
C11302	3	1	6	8	5	2	4	11	10	7	9
C07512	10	5	2	4	6	1	3	8	9	7	11
C03499	5	8	1	11	3	4	9	7	6	10	2
C06956	10	5	3	6	2	8	9	11	4	7	1
C06820	10	9	5	1	11	4	6	7	2	3	8
C01982	1	10	6	9	2	11	8	5	4	7	3
C13816	3	9	5	11	10	8	2	1	4	7	6

C08351	2	4	9	1	10	8	11	7	6	3	5
C01756	4	11	2	8	9	5	10	6	1	7	3
C06941	6	4	11	1	3	10	8	9	7	5	2
C10853	10	11	2	4	9	6	3	5	7	8	1
C07755	1	2	4	5	6	8	10	3	11	9	7
C14347	4	5	8	10	6	3	9	11	1	7	2
C01993	8	2	4	6	1	11	10	3	7	5	9
C02926	5	11	1	3	2	9	10	4	6	8	7
C10651	10	3	5	11	2	4	1	9	8	6	7
C08480	10	5	2	11	3	7	8	6	1	4	9
C08389	11	4	10	9	2	3	7	8	6	5	1
C10644	11	5	9	1	7	10	3	6	2	4	8
C15533	1	5	2	9	8	11	10	7	3	6	4
C01449	6	2	4	8	1	5	3	11	7	9	10
C11684	5	10	11	2	8	1	6	4	3	7	9
C08631	10	3	9	11	2	5	8	7	1	4	6
C15677	5	6	9	3	1	7	8	11	4	2	10
C13834	8	11	9	2	3	7	4	1	5	10	6
C11316	11	9	10	5	1	2	3	4	8	7	6
C11767	10	5	3	6	2	11	9	8	1	4	7
C07921	5	10	11	8	6	1	3	9	4	7	2
C10746	11	10	5	9	4	3	7	8	1	2	6
C08355	1	2	5	10	7	8	3	11	6	9	4
C10851	10	2	9	7	1	6	5	8	4	3	11
C14246	11	2	4	3	6	1	8	7	10	9	5
C01381	1	2	10	5	6	8	3	4	11	9	7
C11205	5	1	10	2	4	11	6	8	3	9	7
C09406	9	7	10	8	3	4	6	2	5	11	1
C08773	1	10	3	2	8	5	11	6	7	9	4
C13173	9	10	6	11	7	5	4	3	8	1	2
C05339	1	2	11	5	6	4	8	7	9	3	10
C01358	2	1	11	5	8	3	6	10	7	4	9
C11390	9	2	8	11	6	1	5	3	7	4	10
C10968	5	10	11	6	8	3	4	2	1	9	7
C09411	10	11	5	8	7	9	4	3	6	1	2
C13061	10	8	5	7	3	9	2	1	11	4	6
C07257	11	4	2	10	9	3	5	8	1	6	7
C08472	5	10	8	11	6	1	9	4	3	2	7
C14195	11	1	2	5	7	9	10	3	6	4	8
C08173	3	5	11	8	7	9	10	1	6	2	4
C11028	11	10	9	2	4	8	6	3	1	7	5
C05071	11	8	6	1	5	7	2	3	10	9	4
C14679	2	8	1	9	6	4	10	11	3	7	5

C12305	9	11	5	10	3	2	6	1	8	4	7
C00753	2	6	8	5	1	9	7	11	4	10	3
C08686	9	2	7	6	3	11	10	1	8	4	5
C06538	10	5	8	3	1	2	4	7	11	9	6
C08414	11	4	5	2	6	10	3	9	7	1	8
C14743	11	9	5	6	10	1	2	4	7	3	8
C00404	4	8	2	1	5	9	6	10	7	3	11
C10132	1	10	11	9	2	7	8	5	3	4	6
C03894	2	8	1	6	10	7	9	11	5	3	4
C07371	11	10	5	4	8	2	9	7	3	6	1
C02037	5	6	2	8	10	7	4	3	11	1	9
C13655	10	1	9	3	4	8	6	2	5	11	7
C10375	10	6	11	1	3	9	4	8	2	7	5
C12847	10	9	5	11	6	1	4	3	7	8	2
C07890	5	3	10	6	1	4	2	8	11	7	9
C02500	1	2	8	5	11	4	6	9	10	3	7
C09792	9	6	11	2	4	10	7	3	5	1	8
C07207	4	11	6	9	1	2	5	10	8	3	7
C07526	1	10	5	2	11	4	6	3	9	8	7
C08636	3	9	10	5	4	7	2	6	11	8	1
C07809	10	7	2	1	5	3	6	4	9	8	11
C14702	11	6	5	2	1	9	8	4	10	7	3
C02341	1	5	2	11	8	6	9	3	4	10	7
C01404	3	5	10	4	11	8	9	2	6	1	7
C08118	10	9	5	6	11	4	7	3	8	1	2
C08246	10	1	3	11	5	6	7	9	8	4	2
C08235	1	8	10	4	5	9	3	7	2	11	6
C14753	3	10	11	9	2	8	4	7	1	6	5
C07391	8	11	9	2	5	10	6	4	3	7	1
C14341	11	5	8	6	4	10	3	2	9	7	1
C10888	10	2	5	4	6	1	9	8	3	7	11
C08810	9	10	3	7	5	8	11	1	6	2	4
C08698	10	9	11	2	6	1	8	4	7	3	5
C04415	4	1	9	8	3	10	5	7	2	11	6
C08447	10	11	9	1	7	2	6	8	3	5	4
C08076	5	11	9	3	2	4	1	7	10	8	6
C14493	3	10	7	5	6	11	4	2	9	8	1
C15436	2	4	5	11	8	3	6	9	7	1	10
C14398	11	5	10	2	6	7	1	3	9	8	4
C13189	11	8	5	6	3	2	4	7	1	10	9
C06478	1	5	2	4	10	8	6	7	11	3	9
C08273	5	9	10	8	6	2	1	4	11	7	3
C05349	10	9	11	4	5	6	8	7	1	3	2

C13841	11	10	8	9	3	7	2	1	4	6	5
C12081	3	1	4	7	10	8	11	9	2	6	5
C09648	9	11	8	3	10	5	7	6	4	2	1
C11701	8	1	11	2	5	3	7	10	9	4	6
C14363	11	7	8	4	9	5	3	10	6	2	1
C10906	1	2	6	8	5	9	10	11	4	3	7
C01192	2	3	6	8	1	7	11	5	10	9	4
C10471	10	11	3	5	8	4	6	1	2	7	9
C14714	11	10	2	7	9	8	5	3	6	1	4
C10138	2	6	8	10	9	1	7	4	3	11	5
C09336	10	6	1	5	8	2	3	4	11	9	7
C14570	11	5	8	3	7	6	10	1	4	9	2
C02240	11	2	8	6	1	9	5	10	3	4	7
C00855	5	2	6	1	8	10	4	9	3	11	7
C14369	11	5	4	8	6	9	7	10	3	1	2
C11093	11	10	9	2	1	8	4	7	5	3	6
C14730	11	5	4	7	3	2	8	6	10	9	1
C00579	5	8	2	1	6	9	11	3	4	10	7
C05902	10	11	8	5	6	1	3	9	7	4	2
C01510	1	2	8	5	4	6	11	10	9	7	3
C00830	2	1	6	8	5	11	3	9	7	4	10
C13140	2	11	7	5	8	4	1	6	9	3	10
C07264	10	3	5	2	1	4	6	9	8	11	7
C08978	1	9	7	11	10	3	4	2	8	5	6
C10178	10	9	11	7	6	3	1	2	4	8	5
C03790	2	8	6	5	11	1	9	3	4	10	7
C07301	11	2	4	6	5	8	10	7	9	3	1
C09067	10	8	6	4	1	3	2	5	11	9	7
C06239	1	4	9	2	10	3	7	8	6	11	5
C10675	10	3	5	11	4	1	6	8	7	2	9
C15230	10	5	11	9	8	4	2	3	6	7	1
C13684	9	10	8	6	1	5	11	4	7	3	2
C15768	1	9	5	4	10	2	8	3	11	6	7
C06229	2	4	9	8	10	7	1	6	3	11	5
C11071	11	10	9	2	5	1	3	6	8	4	7
C08481	5	10	1	9	4	11	3	8	6	7	2
C06345	8	2	4	6	10	3	5	11	1	7	9
C10946	5	2	6	8	1	11	3	10	4	9	7
C01087	1	5	2	8	6	11	3	9	10	4	7
C10807	1	11	5	2	8	9	4	6	3	10	7
C11292	5	3	9	11	10	1	2	8	6	7	4
C09700	10	3	2	1	5	9	11	4	8	6	7
C13948	3	9	10	8	6	4	11	5	7	2	1

C13710	5	6	1	10	8	4	7	2	11	3	9
C11793	10	11	4	5	1	2	6	9	3	8	7
C05021	1	5	2	8	10	6	3	11	9	4	7
C07659	10	8	9	7	3	6	1	2	5	4	11
C03708	1	11	5	2	3	6	9	10	8	7	4
C03851	4	2	5	6	9	10	11	7	1	8	3
C10459	3	10	5	9	6	8	7	11	4	2	1
C11685	5	10	8	11	2	6	3	4	9	1	7
C10943	5	8	1	11	10	6	2	4	9	3	7
C04661	3	8	2	1	9	6	5	4	11	10	7
C09836	9	11	6	5	8	1	7	2	3	4	10
C14371	11	8	5	6	7	3	2	1	9	4	10
C14424	11	4	8	10	5	2	7	3	6	9	1
C00716	5	2	1	6	8	10	3	9	4	11	7
C08186	3	7	10	5	8	2	6	11	1	4	9
C03604	1	8	2	9	5	10	4	7	11	3	6
C07825	1	10	3	5	2	11	8	6	9	7	4
C00303	5	2	1	6	8	4	10	9	11	3	7
C14254	3	8	11	1	10	6	4	5	9	7	2
C15657	3	6	1	9	7	8	5	11	10	2	4
C12661	8	6	3	5	9	2	11	1	10	7	4
C11303	3	6	10	5	8	7	4	9	11	1	2
C10510	10	5	1	4	3	11	8	7	9	2	6
C14469	11	4	5	8	2	6	7	1	3	9	10
C16138	2	1	6	7	11	5	4	10	3	9	8
C03339	1	2	5	8	4	10	3	11	9	6	7
C10282	10	6	4	7	5	11	9	2	3	1	8
C16457	5	11	10	7	4	3	9	2	1	8	6
C13705	1	2	6	11	8	4	3	5	10	9	7
C10660	10	2	9	6	11	8	1	4	3	5	7
C15277	3	5	6	10	2	11	9	7	8	1	4
C00115	1	11	5	2	8	6	3	4	10	9	7
C08187	3	5	10	4	1	6	2	11	7	9	8
C01951	9	11	10	2	1	5	8	3	6	4	7
C06969	3	5	2	4	11	1	6	10	8	7	9
C15500	8	5	1	6	9	7	10	3	2	11	4
C01760	10	2	9	8	11	5	3	7	1	4	6
C15759	8	6	11	2	4	1	3	10	5	9	7
C06931	11	8	2	6	4	5	3	10	9	1	7
C12667	10	1	11	5	8	4	3	9	6	2	7
C12088	5	6	2	8	10	1	3	9	11	4	7
C00968	4	10	1	7	2	6	3	9	8	11	5
C08907	1	10	11	3	2	5	8	9	6	4	7

C16501	9	2	6	8	3	1	10	7	5	11	4
C08126	10	9	5	11	3	1	6	7	8	2	4
C01843	6	5	8	2	4	10	7	3	9	11	1
C09213	5	11	4	9	1	6	3	10	8	7	2
C15026	10	5	6	11	3	1	2	8	4	9	7
C15754	5	9	4	8	6	2	11	3	10	7	1
C11372	11	4	8	9	5	2	10	6	1	3	7
C10670	11	5	10	3	6	2	7	1	9	8	4
C13540	10	5	11	8	3	4	9	1	2	6	7
C01811	1	2	5	4	6	3	9	8	7	10	11
C03954	11	2	4	8	5	7	3	10	1	6	9
C12079	1	10	5	11	6	8	2	9	4	3	7
C03097	1	10	11	5	6	7	2	8	4	3	9
C03153	1	2	3	5	4	10	11	6	8	7	9
C11017	5	11	9	4	1	2	8	3	7	6	10
C01411	9	1	11	8	2	10	7	6	5	4	3
C14587	3	7	6	5	4	11	9	2	8	1	10
C13740	4	2	10	1	8	5	9	6	7	3	11
C06973	9	10	5	1	6	8	2	3	11	4	7
C05602	5	10	11	8	1	9	2	3	4	6	7
C13591	10	1	5	3	2	4	7	9	11	8	6
C07672	11	1	10	9	6	2	5	8	4	7	3
C08763	10	3	8	2	6	5	11	1	4	7	9
C10180	3	10	6	2	8	4	5	11	7	9	1
C04147	1	10	2	9	7	8	6	11	3	4	5
C06574	1	10	9	8	5	6	4	3	2	11	7
C13736	5	10	6	8	2	11	1	3	9	4	7
C14447	11	5	8	2	6	4	9	1	10	7	3
C11021	11	1	8	2	6	7	9	3	5	10	4
C03683	5	9	11	4	6	2	8	10	3	1	7
C06057	3	8	1	6	4	2	10	5	9	11	7
C13766	3	10	1	9	11	5	6	2	8	7	4
C05410	1	2	8	10	9	6	7	11	4	3	5
C03757	11	6	5	1	4	3	7	2	8	9	10
C14392	3	2	8	11	6	4	9	5	10	1	7
C03281	10	2	4	9	5	7	8	6	11	3	1
C10112	10	8	5	11	9	6	3	4	1	2	7
C09235	10	5	1	4	7	2	3	11	9	8	6
C14204	3	2	6	11	4	5	8	10	1	9	7
C10379	1	11	4	8	6	7	10	3	9	2	5
C00920	6	1	2	5	8	3	11	9	4	10	7
C02008	9	2	4	3	6	11	10	8	7	5	1
C07316	3	10	11	5	9	8	2	6	4	7	1

C09689	3	9	10	8	7	11	5	4	1	6	2
C00950	1	5	2	8	6	4	11	3	9	10	7
C02516	5	6	1	8	2	11	10	4	9	3	7
C15727	5	2	1	7	3	9	8	11	10	4	6
C10681	11	2	7	8	1	4	3	10	5	6	9
C11283	11	1	5	3	2	6	10	9	8	4	7
C15542	1	7	4	9	2	8	10	3	6	11	5
C10673	11	8	4	10	2	9	3	1	7	5	6
C08674	3	6	5	1	2	11	8	7	10	9	4
C07395	3	9	2	1	5	10	7	11	8	6	4
C06306	9	11	3	8	2	5	1	6	10	7	4
C09252	5	2	4	9	10	7	3	1	11	6	8
C02476	1	2	5	10	3	6	8	9	4	11	7
C12148	2	8	1	5	11	6	3	4	7	10	9
C06824	10	5	11	2	3	9	6	4	8	7	1
C07870	11	5	9	8	2	7	6	1	3	10	4
C15583	11	5	2	9	4	8	10	3	6	1	7
C09400	10	8	1	2	5	7	6	4	9	3	11
C14034	9	10	6	5	11	8	1	7	3	2	4
C08644	10	6	1	2	9	4	3	7	5	8	11
C14300	11	4	1	3	5	9	8	10	7	2	6
C11115	1	11	5	9	10	3	6	2	7	8	4
C14518	11	2	1	10	3	7	8	5	4	9	6
C13940	1	11	3	10	6	2	4	9	8	7	5
C14436	1	10	7	11	5	9	2	4	3	6	8
C12981	8	3	9	10	11	1	2	5	4	7	6
C15638	3	8	9	10	2	6	5	1	11	4	7
C05058	11	5	2	8	6	9	4	3	7	10	1
C05229	8	2	11	9	5	10	6	3	4	1	7
C10299	10	9	11	5	7	3	6	8	2	4	1
C11365	11	8	2	10	5	1	7	3	6	4	9
C04644	3	4	2	9	10	8	1	5	6	11	7
C08252	1	10	5	8	11	9	6	3	2	7	4
C14746	11	10	5	9	4	2	7	6	3	8	1
C07548	10	11	2	5	4	1	6	8	3	9	7
C11725	10	4	1	3	9	2	5	11	7	6	8
C14399	11	1	3	10	5	4	8	6	9	2	7
C10616	9	11	10	7	1	3	8	5	4	6	2
C10172	5	8	10	3	2	11	6	4	1	9	7
C15279	11	1	7	6	8	5	10	2	3	4	9
C06799	9	4	10	8	3	11	7	1	2	5	6
C09347	10	1	4	8	2	6	3	9	5	11	7
C13489	3	6	9	4	1	2	8	11	5	7	10

C06922	3	5	11	10	9	1	2	8	6	4	7
C10095	3	6	9	1	2	7	10	5	8	4	11
C08304	5	10	9	2	6	4	11	3	7	1	8
C15719	6	8	5	2	9	3	10	11	4	1	7
C10080	11	9	10	2	6	5	3	4	7	8	1
C10357	10	4	5	7	11	3	2	8	9	6	1
C09103	2	9	4	1	11	10	3	6	7	8	5
C03619	1	2	8	5	6	10	11	7	4	3	9
C03731	2	9	10	4	11	1	5	3	6	7	8
C04166	10	2	9	8	11	5	3	6	1	4	7
C00897	1	2	3	5	8	9	6	4	10	11	7
C04883	6	2	8	11	7	1	4	3	9	5	10
C06681	11	9	10	4	8	1	2	5	6	7	3
C14479	3	11	8	9	2	6	5	1	10	7	4
C15721	4	9	5	10	1	8	7	3	2	11	6
C05348	1	2	3	8	10	9	5	4	11	6	7
C04423	2	8	4	7	9	6	11	10	3	5	1
C11697	10	3	5	4	2	9	6	1	8	11	7
C10897	11	3	4	10	2	5	1	8	9	7	6
C10542	10	2	9	3	5	7	1	4	11	6	8
C08034	10	9	6	8	4	7	3	11	1	2	5
C13743	4	9	1	2	10	5	7	3	6	8	11
C16265	1	2	7	4	5	3	10	9	11	6	8
C10380	10	1	6	5	4	2	9	3	8	7	11
C08243	1	8	2	10	5	6	4	3	7	11	9
C13843	3	6	10	9	8	11	2	1	7	4	5
C02136	11	5	2	8	10	1	4	6	3	9	7
C10161	10	11	8	5	7	6	9	4	3	1	2
C01690	1	3	10	11	8	2	5	9	6	4	7
C00612	5	6	2	10	8	3	11	9	1	4	7
C11143	1	2	8	5	11	10	6	3	4	9	7
C09169	9	5	8	1	2	11	4	6	3	10	7
C12865	8	10	11	1	9	3	6	2	4	5	7
C10114	10	11	5	2	3	9	7	6	1	8	4
C11217	5	10	3	6	8	11	9	2	1	7	4
C10346	10	11	1	4	5	3	2	6	7	8	9
C09731	10	8	11	5	2	6	9	1	3	4	7
C10089	10	5	9	8	6	2	3	7	11	4	1
C01938	11	9	8	10	5	1	7	3	6	4	2
C07485	5	10	8	4	1	2	6	11	3	9	7
C14287	8	1	6	7	4	11	3	5	10	2	9
C06334	11	1	4	9	8	3	6	10	2	5	7
C11661	9	2	8	10	5	6	1	3	11	7	4

C15743	5	10	6	1	2	3	8	9	11	7	4
C12170	10	8	11	4	7	3	1	2	9	6	5
C03687	10	1	6	11	8	5	2	4	9	7	3
C09060	11	10	5	2	4	8	1	7	6	3	9
C06763	1	6	10	5	2	9	11	3	7	8	4
C11782	10	11	8	2	5	9	6	4	3	7	1
C03079	8	5	6	2	1	10	4	9	7	11	3
C09357	10	5	3	9	1	4	2	11	6	8	7
C12933	9	5	11	6	8	3	4	2	7	10	1
C10923	11	5	2	10	9	8	1	3	4	6	7
C08705	10	1	2	4	5	6	8	9	11	3	7
C01093	1	8	10	6	9	7	3	4	11	5	2
C05990	5	9	10	1	2	4	7	11	8	3	6
C04685	3	10	4	8	2	6	11	9	7	1	5
C07973	10	5	11	2	6	1	4	8	3	9	7
C09304	3	1	10	8	6	7	11	9	2	5	4
C00715	8	2	5	6	4	10	1	11	9	7	3
C14015	5	6	7	11	8	1	3	9	2	4	10
C14344	10	11	6	9	1	7	3	8	5	4	2
C06717	2	1	11	5	8	3	6	9	10	7	4
C00528	1	4	8	11	10	3	6	9	7	5	2
C13831	3	8	11	1	10	4	6	5	2	9	7
C10828	11	5	10	8	4	2	7	3	1	9	6
C13415	11	9	6	1	4	3	8	10	2	7	5
C10507	11	7	4	8	9	3	5	1	10	2	6
C10278	10	1	5	4	6	3	2	7	9	11	8
C10866	3	7	9	4	6	2	11	1	8	10	5
C04931	10	3	4	5	11	2	9	6	8	1	7
C05117	5	2	1	3	11	9	6	8	4	7	10
C11758	1	10	9	2	4	8	6	11	5	7	3
C02846	11	2	8	6	10	4	5	9	1	7	3
C09027	10	9	2	8	11	3	5	4	1	7	6
C07287	9	1	11	2	5	3	8	6	10	7	4
C07409	10	9	11	8	5	6	1	7	4	3	2
C14219	11	8	5	2	1	6	9	7	4	3	10
C14674	3	1	2	5	7	9	11	10	8	4	6
C04078	5	2	8	6	3	1	9	4	11	10	7
C01666	11	3	8	2	6	10	1	5	9	4	7
C09882	9	11	3	2	8	5	1	6	10	7	4
C00711	1	5	2	6	8	3	11	9	10	4	7
C09977	11	6	1	5	3	7	8	10	2	4	9
C07840	11	5	3	7	2	10	1	6	9	4	8
C08476	11	5	6	1	2	4	7	3	10	8	9

C08607	9	8	1	3	5	10	2	11	4	6	7
C02389	11	8	1	2	5	10	6	9	4	3	7
C03735	1	2	5	8	4	6	3	9	10	7	11
C10910	11	3	10	8	5	6	9	1	7	2	4
C11060	9	2	1	3	8	5	6	11	4	10	7
C14645	3	5	7	9	8	4	6	11	10	2	1
C14704	11	2	1	3	6	5	4	10	8	9	7
C03801	8	2	10	11	1	6	4	3	9	7	5
C10177	10	11	9	5	4	1	7	8	3	6	2
C03864	2	6	8	1	9	3	7	4	5	11	10
C05887	1	6	5	2	9	8	3	7	4	11	10
C12616	1	10	9	5	11	8	2	3	6	7	4
C06751	2	8	6	5	10	9	11	3	7	1	4
C06535	10	5	11	3	1	6	8	2	4	9	7
C14457	11	7	10	9	8	1	3	4	5	2	6
C08379	9	3	4	1	6	7	10	11	5	2	8
C13778	4	2	5	10	3	6	7	9	11	1	8
C03359	11	5	6	3	2	1	10	9	8	7	4
C10925	11	5	1	3	6	8	10	2	4	9	7
C11145	2	8	6	5	11	1	10	4	3	9	7
C14516	1	10	5	8	11	6	9	3	2	4	7
C11743	10	5	4	9	11	2	3	1	6	7	8
C03796	9	4	2	10	8	1	6	3	7	5	11
C08150	3	9	11	5	10	2	8	1	7	6	4
C03062	1	5	2	8	6	9	7	3	4	10	11
C10122	10	11	5	3	4	6	9	8	1	7	2
C14668	3	11	5	9	4	1	6	2	8	7	10
C15040	5	1	11	6	8	2	10	9	7	3	4
C03570	1	2	5	4	6	3	9	8	7	10	11
C08024	10	11	2	5	8	1	7	9	3	6	4
C11357	10	11	3	5	8	6	9	4	2	1	7
C09010	10	5	8	3	4	1	6	2	9	11	7
C07363	5	10	11	4	9	8	3	6	2	7	1
C07785	5	10	6	8	1	3	9	4	11	2	7
C01942	3	2	5	6	1	9	7	8	11	4	10
C07712	3	8	6	1	4	2	11	7	9	5	10
C01567	1	4	2	7	3	11	10	8	6	5	9
C02787	5	1	2	10	8	6	11	4	3	9	7
C10061	3	5	11	9	8	10	4	7	1	6	2
C09549	10	4	9	8	3	2	11	6	7	1	5
C03536	4	1	5	6	7	8	9	10	3	2	11
C14487	3	9	10	4	6	11	5	1	2	7	8
C11124	11	8	1	3	4	2	6	10	7	9	5

C00913	2	11	9	5	4	3	7	8	6	10	1
C07072	1	5	10	2	3	6	4	11	8	9	7
C07178	8	10	5	11	9	3	6	4	2	1	7
C01255	8	11	2	6	9	4	3	5	10	1	7
C03817	5	9	11	2	7	1	4	3	6	10	8
C08179	3	8	9	11	2	5	7	6	1	4	10
C03431	5	10	8	1	3	4	9	11	6	2	7
C09780	3	1	10	6	11	9	2	4	8	5	7
C13595	10	5	4	3	2	8	11	1	6	9	7
C04643	3	2	8	6	7	9	5	10	1	11	4
C10571	1	5	8	2	6	10	9	7	3	11	4
C04721	3	6	8	11	2	10	9	1	5	7	4
C08675	9	2	7	11	5	1	3	8	10	4	6
C14444	11	4	9	5	6	2	7	3	10	1	8
C09597	10	8	2	1	5	9	3	6	4	11	7
C04787	10	2	7	8	3	11	6	9	5	1	4
C07965	1	10	4	3	9	11	5	2	6	7	8
C13872	1	3	10	6	9	5	4	11	7	2	8
C15486	6	8	1	5	10	2	7	11	3	4	9
C15604	8	9	2	5	4	6	10	11	1	3	7
C15622	3	9	5	7	1	6	11	8	2	4	10
C11692	5	10	6	8	2	11	1	4	3	9	7
C10073	10	1	5	11	9	4	3	2	8	6	7
C00265	1	5	2	8	6	4	11	3	9	10	7
C14750	10	3	1	9	11	2	8	5	6	7	4
C10326	10	8	2	4	6	1	3	11	5	9	7
C12864	8	11	5	3	2	4	10	6	1	7	9
C11151	6	1	2	11	8	9	7	3	5	10	4
C04562	2	6	8	11	9	1	3	7	5	10	4
C02837	5	8	10	11	2	6	1	3	9	7	4
C04793	8	2	11	3	4	5	7	1	6	9	10
C07037	5	10	11	2	6	7	8	3	9	4	1
C15038	2	8	11	1	4	6	5	10	3	9	7
C09863	9	11	5	6	2	1	7	10	4	8	3
C03066	5	6	11	4	1	2	9	8	3	10	7
C11297	10	8	5	11	2	6	9	3	1	4	7
C01418	11	10	9	1	6	2	7	5	3	8	4
C14359	11	1	8	5	2	9	6	4	3	10	7
C11794	10	11	4	2	1	5	9	6	8	3	7
C11311	1	2	6	5	3	8	4	11	10	9	7
C13714	1	5	6	10	7	11	3	8	4	9	2
C06267	2	11	8	6	5	1	4	7	9	10	3
C09989	1	11	5	8	9	4	6	10	7	3	2

C08513	3	10	5	2	1	11	8	7	9	4	6
C08154	3	4	9	10	11	6	8	2	7	1	5
C10625	3	1	11	5	2	6	10	7	8	9	4
C10097	10	3	5	11	8	1	6	4	9	2	7
C15617	3	11	1	5	8	9	6	7	2	4	10
C08393	3	10	1	8	6	11	2	7	5	4	9
C13814	3	5	8	7	11	1	2	4	6	9	10
C06338	11	1	3	5	4	2	10	9	7	8	6
C07577	5	10	11	2	8	1	3	9	6	4	7
C10362	10	8	5	2	11	9	3	1	7	6	4
C13424	5	1	8	10	11	2	6	3	7	4	9
C15265	3	6	2	8	5	11	7	10	4	9	1
C07590	3	10	1	11	9	2	7	6	8	4	5
C07171	10	5	11	4	6	9	8	3	1	2	7
C10125	10	8	1	4	9	6	11	2	3	5	7
C11379	2	6	10	5	8	9	4	11	1	3	7
C09236	10	5	9	2	6	7	3	4	11	1	8
C11730	8	9	5	11	2	10	3	7	4	6	1
C08494	5	10	7	2	1	9	3	4	8	6	11
C09280	10	9	2	3	5	6	7	1	11	4	8
C09186	9	1	8	11	7	5	3	6	10	4	2
C04024	10	9	4	11	5	7	1	6	3	2	8
C01062	1	2	6	8	10	9	5	7	3	11	4
C13353	10	6	9	1	5	2	3	11	8	7	4
C13029	5	3	10	7	2	6	8	11	1	9	4
C09830	10	6	11	9	5	3	8	4	7	1	2
C13612	3	1	10	8	2	6	11	9	5	4	7
C08886	4	1	9	3	8	7	11	10	6	5	2
C09129	10	7	8	6	2	11	3	1	4	5	9
C13144	3	10	5	1	9	8	6	11	7	4	2
C11253	6	10	1	5	8	2	9	11	3	7	4
C03662	10	4	5	2	9	1	3	7	8	6	11
C09163	1	7	10	9	8	6	3	11	4	5	2
C13883	3	6	8	2	9	11	5	4	1	7	10
C15643	3	5	11	10	9	1	8	4	2	6	7
C14002	9	10	1	6	5	8	11	3	2	7	4
C03686	9	11	6	8	2	3	7	5	4	10	1
C10402	11	10	3	5	6	8	9	2	1	7	4
C10658	3	5	10	7	2	8	11	1	4	9	6
C07816	3	6	4	7	1	10	5	9	11	2	8
C01328	5	1	11	10	8	9	3	4	6	2	7
C04010	1	2	8	4	11	6	3	5	10	9	7
C08289	6	2	9	5	4	8	7	1	10	3	11

C01807	1	8	2	5	9	11	10	3	6	7	4
C13906	10	2	4	5	8	9	3	11	1	7	6
C12068	5	3	1	11	6	2	4	10	9	8	7
C02634	5	2	6	8	3	1	4	11	10	9	7
C11690	10	5	8	11	2	3	9	4	1	7	6
C02817	6	2	8	3	1	10	4	7	11	9	5
C01633	11	3	1	10	9	2	5	7	4	8	6
C08941	11	5	8	10	2	3	4	6	1	7	9
C03196	1	5	2	8	6	11	3	9	10	4	7
C02343	5	10	9	11	8	2	1	7	3	6	4
C09403	10	5	1	11	3	9	8	7	6	4	2
C12181	10	11	1	8	2	3	6	5	9	7	4
C11045	1	5	10	8	2	11	3	6	7	9	4
C06883	10	9	6	5	11	1	3	8	2	4	7
C10942	11	5	4	1	2	3	9	7	10	6	8
C08046	10	9	1	2	6	5	3	4	8	7	11
C07522	3	1	11	10	5	2	6	4	9	8	7
C07605	10	6	5	8	2	1	9	3	7	11	4
C09295	10	1	3	7	2	4	11	5	6	8	9
C02287	1	2	8	5	11	4	6	9	10	3	7
C08092	6	4	1	9	5	3	11	2	7	8	10
C08406	10	5	1	8	6	4	2	3	11	9	7
C02740	4	2	9	6	8	5	1	10	11	3	7
C02242	4	2	11	8	10	9	5	3	6	7	1
C08671	9	5	6	7	8	1	3	2	10	11	4
C12766	6	5	1	8	10	2	3	7	11	9	4
C09592	10	11	5	2	9	7	4	3	6	1	8
C10492	5	10	11	7	1	4	2	9	8	3	6
C09194	5	3	4	10	1	11	9	8	2	6	7
C00984	1	2	10	5	6	8	3	4	11	9	7
C07914	10	5	11	6	9	7	1	3	8	2	4
C02728	5	8	2	10	11	6	1	9	7	3	4
C11789	10	11	5	4	1	8	6	9	3	7	2
C14368	11	2	10	4	1	7	6	3	8	9	5
C10909	11	5	1	10	6	2	9	3	4	7	8
C16001	8	2	11	10	5	1	3	6	7	9	4
C09069	10	9	5	11	8	1	2	4	3	7	6
C11596	3	1	4	10	5	2	8	9	7	6	11
C02419	6	11	2	7	4	10	3	8	1	5	9
C07613	3	1	9	10	4	7	11	2	5	8	6
C16184	3	9	11	1	6	4	7	8	5	10	2
C11471	9	11	2	10	4	7	6	8	5	3	1
C07558	4	5	9	7	1	2	3	10	6	11	8

C09007	1	10	5	4	2	6	11	3	7	9	8
C03693	1	2	5	10	3	4	9	8	11	7	6
C02381	5	9	1	3	6	11	2	7	8	10	4
C07393	3	9	11	4	10	7	2	5	8	6	1
C06806	5	4	8	9	3	10	2	6	11	1	7
C08180	3	11	9	7	1	2	6	10	8	5	4
C10124	1	10	11	9	4	2	7	6	5	3	8
C04654	6	2	3	8	5	7	9	11	4	10	1
C10589	11	8	6	1	4	2	3	7	5	9	10
C16157	1	2	11	8	7	4	3	10	9	6	5
C14574	11	2	1	8	7	10	4	3	6	9	5
C08290	5	6	8	4	10	2	1	11	3	7	9
C08673	10	5	4	8	9	11	3	6	7	1	2
C03115	9	2	4	3	10	8	7	11	6	5	1
C09668	9	10	5	8	4	6	1	2	3	7	11
C06417	5	6	2	1	8	10	4	9	11	3	7
C09988	9	8	5	1	10	4	11	2	3	6	7
C09906	9	11	5	1	2	6	3	8	7	10	4
C03526	8	3	1	7	5	4	9	10	11	2	6
C11729	1	10	9	8	11	7	3	6	5	2	4
C14429	11	10	9	2	3	1	4	8	5	7	6
C12152	9	1	3	4	5	8	11	6	7	2	10
C07147	10	5	3	11	8	4	1	7	9	6	2
C10338	9	8	1	5	11	10	6	3	7	2	4
C10900	1	11	5	6	10	2	3	9	8	7	4
C08152	3	9	2	6	1	7	10	4	11	8	5
C07275	4	10	11	8	6	5	1	2	3	9	7
C11198	10	2	7	1	8	5	4	6	3	11	9
C08071	3	11	5	9	2	8	10	1	4	6	7
C10187	10	7	2	8	6	11	3	5	9	4	1
C08901	10	9	11	2	1	7	3	4	8	5	6
C07753	1	10	2	4	5	11	9	3	7	6	8
C02976	1	2	9	3	5	8	10	4	6	11	7
C11245	3	1	8	11	6	7	10	5	2	9	4
C07968	3	1	5	6	7	2	4	9	10	11	8
C13522	11	1	10	9	4	5	8	2	6	3	7
C16287	1	5	7	4	11	6	9	3	10	8	2
C10053	10	3	2	8	1	9	4	11	6	5	7
C08185	3	9	4	8	5	2	7	11	10	1	6
C13794	5	1	6	4	8	10	11	7	3	2	9
C06545	3	10	2	5	11	6	9	4	1	7	8
C08449	10	11	3	1	2	5	7	4	8	6	9
C04211	5	9	6	4	7	1	2	3	11	8	10

C04207	5	9	1	2	10	11	3	7	4	8	6
C06682	6	5	8	1	2	9	7	11	4	10	3
C11049	9	3	5	11	1	10	4	8	2	6	7
C03767	2	8	11	5	6	9	10	1	3	7	4
C08272	9	5	3	1	11	4	10	8	2	7	6
C10550	11	10	9	8	2	5	7	1	4	3	6
C14284	3	11	4	9	2	6	5	10	8	1	7
C07523	5	1	11	2	10	6	3	4	8	7	9
C09947	11	10	5	3	2	6	4	1	7	9	8
C01818	2	8	5	6	4	10	11	7	1	3	9
C14405	11	3	9	5	8	10	4	7	1	6	2
C14156	8	9	3	5	11	10	6	4	1	7	2
C14548	3	11	10	8	2	4	1	6	9	7	5
C12552	10	4	11	7	9	3	6	5	2	8	1
C08121	1	11	5	6	9	3	10	7	8	2	4
C07652	3	11	8	1	5	6	4	9	2	10	7
C08014	10	5	11	6	1	8	2	9	3	7	4
C09475	4	9	8	2	1	5	3	11	10	7	6
C14413	11	8	9	10	3	5	2	7	1	6	4
C06738	10	5	11	8	2	1	9	6	4	3	7
C02783	1	4	9	8	10	2	3	11	6	5	7
C07283	1	10	5	2	6	11	8	3	7	4	9
C05700	5	2	6	1	8	9	11	3	10	7	4
C10144	10	1	2	3	8	5	9	11	7	4	6
C10310	11	8	10	9	5	1	6	4	3	2	7
C10339	10	1	8	9	11	6	4	3	2	5	7
C06081	9	1	3	8	2	7	6	10	5	4	11
C04030	9	2	4	11	10	6	8	3	1	7	5
C08164	3	1	2	11	9	7	6	10	8	4	5
C06819	5	6	11	8	9	2	10	4	7	1	3
C09228	11	3	9	5	7	1	10	4	2	6	8
C13055	9	10	1	6	11	5	3	8	7	4	2
C11392	9	8	2	11	3	1	6	4	10	5	7
C02086	5	2	1	8	11	10	6	4	3	9	7
C07565	11	3	5	8	10	6	1	2	9	7	4
C03755	10	7	9	8	4	1	3	2	6	11	5
C12193	10	5	2	11	6	8	3	4	9	1	7
C11001	11	4	2	7	5	6	3	10	1	9	8
C11347	6	5	9	8	3	7	1	11	4	2	10
C09716	9	1	8	11	2	5	6	10	3	4	7
C02040	5	9	10	6	2	1	7	8	11	3	4
C15712	5	10	9	1	8	6	3	2	7	4	11
C12070	5	4	10	8	3	2	11	6	7	9	1

C10740	10	3	9	7	5	11	6	2	1	4	8
C08999	10	3	9	2	1	4	6	7	11	5	8
C02421	8	2	5	11	1	10	4	6	7	3	9
C14058	9	11	2	5	10	1	6	4	3	8	7
C15690	9	1	11	2	5	10	6	8	7	3	4
C10318	10	11	9	8	4	5	6	3	7	2	1
C14214	11	3	4	10	1	5	2	8	7	6	9
C03034	2	6	8	10	9	11	7	3	5	1	4
C05052	1	2	8	9	5	6	4	10	11	3	7
C10721	3	10	4	9	11	2	5	8	6	1	7
C06543	9	3	1	4	10	2	7	8	11	6	5
C01502	11	5	10	9	8	1	6	3	2	4	7
C03761	3	1	11	5	8	6	9	2	10	4	7
C11877	9	2	1	5	6	7	3	10	4	8	11
C06541	8	10	6	9	4	11	1	3	2	5	7
C07758	3	11	4	5	2	1	9	8	7	10	6
C11737	4	2	8	11	5	6	1	7	10	9	3
C10266	10	9	3	11	2	8	6	1	5	4	7
C09640	10	3	5	1	8	4	7	9	6	11	2
C10324	10	2	6	4	1	9	8	5	3	7	11
C03642	3	4	2	7	8	6	5	1	10	11	9
C09036	10	5	6	9	2	1	11	3	4	7	8
C08954	1	10	2	11	8	4	5	6	9	3	7
C11095	9	11	8	5	10	7	4	1	3	2	6
C14504	11	4	5	10	8	9	3	7	6	2	1
C10108	10	11	5	8	3	1	2	4	7	9	6
C04572	1	5	2	7	8	6	9	3	4	10	11
C08001	10	6	11	3	4	9	5	7	8	1	2
C00768	5	6	2	1	8	10	4	9	3	7	11
C06761	11	4	7	2	3	8	10	5	6	9	1
C06926	3	5	4	1	10	9	7	11	2	6	8
C08156	3	1	4	5	2	6	9	7	8	11	10
C08259	1	10	3	7	2	6	11	4	8	5	9
C07931	11	10	8	3	1	2	5	4	9	6	7
C08812	3	9	8	11	2	7	5	10	1	4	6
C14352	1	11	10	9	2	5	3	7	4	6	8
C14592	11	4	10	6	9	2	3	1	5	8	7
C14310	11	5	9	6	1	3	7	2	10	8	4
C09253	10	5	7	4	11	9	2	6	3	1	8
C03444	1	5	11	2	6	8	4	10	3	9	7
C09392	11	7	10	9	1	3	5	2	4	6	8
C11300	5	9	2	1	11	10	6	3	8	4	7
C00481	2	11	5	6	3	1	10	8	9	7	4

C03074	5	9	1	11	4	8	3	6	2	10	7
C02493	1	10	11	9	3	6	2	4	7	5	8
C02838	3	5	1	8	9	11	2	7	6	4	10
C13803	3	1	5	6	2	4	8	9	11	7	10
C03392	5	2	6	10	3	11	4	1	9	8	7
C06305	9	11	2	8	5	1	7	6	10	4	3
C10485	10	5	2	8	11	7	1	6	4	9	3
C10342	8	10	6	11	5	2	7	1	9	3	4
C11238	3	1	10	2	4	5	6	8	11	9	7
C01948	2	5	3	1	11	8	6	4	10	7	9
C07939	5	10	6	2	11	8	4	1	9	3	7
C07982	5	10	11	3	2	9	4	8	6	1	7
C08346	1	10	7	3	4	9	6	2	11	5	8
C13142	10	6	7	11	5	1	3	8	4	2	9
C10349	10	11	3	1	9	4	6	8	2	7	5
C14431	11	10	9	1	3	2	8	6	7	5	4
C06775	6	8	7	5	2	10	9	11	3	1	4
C08106	10	9	1	2	6	5	8	11	3	4	7
C14534	11	5	8	10	7	3	6	2	9	1	4
C09315	10	11	2	5	8	1	3	9	4	6	7
C11000	1	11	3	9	10	5	7	8	6	4	2
C08720	11	10	2	8	4	9	3	1	6	5	7
C15737	10	8	7	6	11	2	3	9	5	4	1
C14993	11	3	10	5	2	8	1	4	6	7	9
C02953	8	2	5	6	11	10	1	4	9	7	3
C10449	9	10	11	5	8	4	2	3	7	6	1
C14482	3	1	2	8	6	11	10	7	9	4	5
C04426	1	7	4	2	8	10	5	3	9	11	6
C15988	3	1	11	6	7	10	5	9	4	8	2
C11343	1	3	2	5	10	8	11	9	6	7	4
C07297	3	6	1	5	9	8	7	2	4	10	11
C08095	1	4	3	5	10	2	6	9	11	8	7
C09518	11	10	6	1	2	4	9	8	7	3	5
C07877	5	10	2	11	6	8	9	3	7	1	4
C08423	10	9	5	3	7	4	8	1	11	2	6
C08293	6	10	8	9	11	3	5	7	1	4	2
C14409	11	3	1	2	9	8	5	6	7	10	4
C02962	1	2	5	8	4	6	3	9	10	7	11
C06905	5	10	11	3	9	1	8	2	7	6	4
C10129	10	5	8	3	11	1	4	9	7	2	6
C11002	11	10	9	5	7	3	1	6	8	4	2
C10719	3	10	5	11	1	8	9	4	6	2	7
C08680	9	10	1	11	4	8	5	3	2	7	6

C14608	3	10	2	7	11	1	8	5	4	9	6
C09724	10	3	5	8	7	9	1	6	11	4	2
C14562	11	2	4	5	6	10	3	8	9	1	7
C08660	9	11	2	1	3	10	4	8	7	6	5
C04102	3	11	5	6	1	4	10	7	8	9	2
C09594	10	7	6	3	5	4	2	9	1	11	8
C02227	11	5	2	3	4	8	9	1	6	7	10
C14554	3	5	2	6	9	1	4	10	7	8	11
C14642	3	9	10	1	8	2	6	5	11	4	7
C01882	2	8	1	4	5	9	6	10	11	3	7
C16250	4	11	2	1	8	5	3	6	10	9	7
C14583	11	9	8	7	2	3	10	1	4	6	5
C14510	11	2	5	6	8	1	9	10	7	3	4
C14501	8	5	11	6	10	1	9	3	2	4	7
C02674	1	4	2	5	8	6	11	10	3	7	9
C14285	11	5	10	8	9	3	1	2	4	7	6
C06997	3	11	10	8	1	5	9	7	6	2	4
C11144	11	10	1	2	9	3	5	6	8	7	4
C06842	3	5	10	8	11	1	2	7	4	9	6
C03342	1	2	8	6	9	5	4	10	11	7	3
C04534	1	2	10	9	8	6	5	4	11	7	3
C06262	1	3	2	5	8	6	10	11	9	4	7
C08909	1	3	4	10	6	5	11	8	7	9	2
C10345	10	3	2	6	9	4	11	1	7	5	8
C14602	11	1	5	2	10	7	6	9	3	8	4
C08861	11	10	3	2	6	7	8	5	4	1	9
C01012	8	6	1	2	4	11	5	7	3	10	9
C09795	10	1	9	5	3	2	4	11	7	6	8
C10701	10	5	11	3	7	4	8	2	1	6	9
C12053	5	6	9	10	11	8	1	3	2	7	4
C09341	6	5	2	4	9	1	11	3	8	10	7
C03592	4	5	9	8	6	11	10	1	7	3	2
C09133	10	5	9	7	11	4	8	6	3	2	1
C07514	5	10	11	6	8	1	3	2	4	9	7
C10350	5	10	6	3	2	11	4	7	8	1	9
C15481	1	10	7	2	11	5	4	9	3	8	6
C14662	6	2	10	11	8	9	4	3	5	1	7
C14241	10	4	5	2	7	3	6	9	11	8	1
C01205	4	6	5	8	1	2	11	10	9	7	3
C04318	5	9	11	8	2	1	6	3	10	4	7
C12327	10	8	11	1	4	7	3	6	9	2	5
C03969	5	10	6	2	8	1	9	4	7	3	11
C13683	10	8	6	4	5	2	11	7	9	3	1

C06979	10	9	11	4	5	6	8	7	3	1	2
C14291	11	10	9	2	5	1	3	6	7	8	4
C08437	1	6	10	5	2	9	3	11	7	8	4
C10377	8	9	2	11	10	5	7	3	6	1	4
C08333	6	1	3	9	5	10	11	4	8	7	2
C14528	11	3	5	4	2	8	9	10	7	6	1
C14527	11	1	3	2	5	7	10	8	9	6	4
C14577	11	4	9	8	10	3	1	2	6	5	7
C10493	9	4	8	2	10	7	3	1	11	6	5
C15608	10	1	11	5	8	2	3	9	7	6	4
C03187	1	10	9	2	4	8	6	3	5	7	11
C08536	10	11	9	5	2	8	1	6	3	4	7
C09285	10	11	9	1	6	3	8	4	2	5	7
C14223	3	6	10	11	8	2	5	7	1	4	9
C01289	1	9	7	4	10	3	5	6	8	11	2
C08366	3	8	11	6	4	1	2	5	9	10	7
C01725	1	6	5	8	3	7	11	4	9	10	2
C03629	2	4	1	8	3	6	10	7	5	9	11
C10477	10	5	11	3	8	2	7	1	9	4	6
C10078	10	3	11	5	2	8	9	4	1	7	6
C14302	11	6	10	3	9	5	1	2	4	8	7
C02845	11	1	6	5	3	9	2	10	8	4	7
C00248	2	8	5	6	1	11	3	4	9	10	7
C06474	1	2	5	4	10	8	3	11	7	6	9
C10834	10	11	5	1	2	7	8	3	6	4	9
C01842	2	6	8	4	5	3	11	10	7	1	9
C08234	9	8	1	11	7	4	2	5	3	10	6
C13877	3	9	5	1	10	7	6	4	11	8	2
C09080	10	1	7	9	2	3	5	8	4	11	6
C03775	3	2	8	6	9	11	5	1	10	4	7
C14366	11	2	7	6	5	8	3	10	9	1	4
C15527	8	5	9	2	1	6	10	3	11	7	4
C10438	10	5	11	2	8	9	1	6	3	4	7
C14154	8	9	10	5	11	1	2	4	3	6	7
C14677	11	3	8	5	7	1	4	9	2	6	10
C11228	3	11	9	10	2	6	4	7	1	8	5
C03998	5	6	2	1	9	11	10	3	4	7	8
C06355	2	6	8	5	3	1	11	9	4	7	10
C02234	11	8	6	5	2	10	9	3	1	4	7
C10382	11	4	6	8	7	2	9	3	10	5	1
C01394	1	2	8	5	4	6	11	10	9	7	3
C03927	9	2	11	8	6	5	10	1	7	3	4
C07599	5	4	10	6	2	11	8	9	1	7	3

C05308	9	2	8	5	4	6	3	1	11	7	10
C14351	8	2	10	5	1	9	3	6	11	7	4
C13235	5	10	4	3	2	1	8	11	6	7	9
C14692	11	4	5	6	2	7	1	9	3	8	10
C03485	11	1	5	9	2	10	8	3	6	4	7
C04875	6	2	8	5	9	10	3	11	4	7	1
C07941	10	5	11	7	4	8	1	3	2	6	9
C10829	5	11	7	8	9	3	6	1	10	4	2
C10543	10	11	9	5	2	8	7	4	3	6	1
C09786	10	11	5	2	6	7	9	4	3	8	1
C08504	6	3	10	4	1	8	2	9	11	5	7
C01581	1	2	7	4	9	10	3	8	6	5	11
C01474	9	4	1	11	8	3	10	6	2	5	7
C08284	5	8	10	6	1	7	3	9	2	11	4
C14459	11	1	6	2	9	10	3	7	4	5	8
C02884	10	4	9	2	1	6	11	8	3	5	7
C08430	1	10	6	5	4	9	8	11	3	2	7
C16489	1	10	7	11	8	9	4	3	5	6	2
C10021	11	10	3	5	1	9	6	2	8	7	4
C07510	10	5	3	6	2	8	4	1	11	9	7
C13782	10	5	3	11	2	8	9	1	7	6	4
C08005	10	11	5	1	2	6	4	3	8	9	7
C13143	10	9	5	11	8	6	1	3	2	4	7
C06853	10	5	2	9	1	6	8	7	3	11	4
C08371	5	2	1	9	11	3	6	10	4	7	8
C00397	10	9	1	5	8	3	4	6	11	2	7
C15571	11	10	5	1	8	2	3	6	9	4	7
C15627	3	8	1	9	10	5	2	4	6	11	7
C07927	11	10	3	9	6	7	1	4	8	2	5
C10896	11	10	9	3	2	6	5	8	1	4	7
C08528	10	5	8	6	4	2	3	7	1	11	9
C04050	11	8	2	4	5	1	10	7	6	9	3
C04255	1	2	10	5	4	8	7	3	9	11	6
C07966	9	3	8	5	2	1	11	10	6	4	7
C09471	9	4	7	5	6	11	2	3	8	10	1
C06803	10	5	4	1	3	2	9	11	7	8	6
C08108	10	9	6	5	8	4	7	2	3	1	11
C01988	11	2	8	5	6	9	1	10	3	4	7
C07999	9	10	5	11	1	6	2	8	3	7	4
C01342	5	1	2	8	11	4	6	10	3	9	7
C10672	10	11	1	6	2	9	3	8	7	5	4
C03965	3	10	9	2	6	5	1	8	7	11	4
C06464	1	2	10	5	6	8	3	4	11	9	7

C07290	2	8	6	10	7	3	11	5	1	4	9
C09238	10	3	5	4	8	7	9	1	11	6	2
C01864	8	2	5	11	6	10	9	4	3	1	7
C14452	2	11	3	5	10	6	1	9	4	8	7
C12298	9	11	5	1	3	2	10	8	4	6	7
C14029	5	11	10	9	8	7	6	3	4	1	2
C07455	5	3	2	6	10	8	4	11	1	9	7
C11227	5	4	9	2	11	10	6	3	1	8	7
C06917	3	11	1	4	10	6	2	5	7	9	8
C09979	2	10	11	5	8	1	6	4	3	7	9
C11058	11	9	6	10	8	3	5	1	4	7	2
C11917	9	2	6	1	4	8	11	5	3	7	10
C07410	10	5	11	2	4	3	6	9	1	7	8
C06463	1	2	8	5	3	10	6	7	9	11	4
C08521	10	11	2	4	9	3	8	6	5	1	7
C10729	10	1	9	8	7	11	5	6	3	2	4
C01970	1	2	3	5	8	9	6	4	10	11	7
C14515	11	1	5	3	8	4	2	6	9	10	7
C11804	5	10	8	6	9	7	4	3	2	1	11
C02001	11	2	1	6	9	8	3	10	7	5	4
C11116	5	11	8	9	7	4	10	3	2	1	6
C10046	10	5	1	9	3	2	11	6	7	8	4
C11073	11	9	8	2	1	4	3	5	10	7	6
C12112	1	9	4	3	2	7	5	6	11	8	10
C14078	6	5	1	8	2	4	11	9	10	7	3
C11299	9	3	8	5	6	1	2	11	4	7	10
C02267	4	9	11	5	10	2	1	8	3	6	7
C09712	9	10	5	3	11	2	7	8	1	4	6
C09042	5	10	4	11	9	3	2	7	8	6	1
C12041	11	5	1	7	8	6	3	2	4	9	10
C02328	1	9	10	5	11	3	8	7	2	6	4
C07544	5	10	3	11	2	9	7	4	1	8	6
C01854	10	1	9	11	4	7	5	3	6	2	8
C07474	10	5	1	6	8	11	3	2	4	9	7
C14299	11	6	9	2	8	10	4	3	7	1	5
C07616	3	11	2	9	5	4	8	10	1	7	6
C02774	2	11	3	5	8	10	1	9	4	6	7
C07195	4	1	2	5	8	10	6	9	3	11	7
C10236	10	11	1	5	3	2	8	6	4	9	7
C01748	6	4	5	8	2	9	11	7	1	3	10
C06332	2	11	5	8	10	9	3	4	1	6	7
C02213	2	8	9	5	11	4	3	1	10	6	7
C08228	10	5	11	6	8	9	1	3	4	7	2

C01551	4	5	2	8	1	6	11	10	9	3	7
C15226	8	5	7	4	2	3	9	10	1	11	6
C03349	5	6	10	11	1	2	8	4	3	9	7
C10358	3	11	6	5	10	7	9	8	1	4	2
C00994	5	10	6	2	1	9	8	4	11	3	7
C07166	5	6	1	8	10	2	9	4	3	7	11
C12256	5	1	11	7	10	4	9	3	6	8	2
C15710	10	11	6	5	4	7	2	9	3	1	8
C05403	1	5	8	2	6	9	10	7	3	11	4
C09146	5	10	1	11	9	2	3	6	7	4	8
C14163	11	9	8	5	4	1	3	7	6	2	10
C04300	3	1	9	4	7	10	11	8	2	5	6
C08288	5	6	10	8	4	9	7	2	11	3	1
C09467	10	11	1	3	2	6	7	5	8	9	4
C09364	5	6	10	8	1	11	3	2	4	9	7
C10911	11	6	8	4	2	5	1	9	3	7	10
C08599	9	8	3	10	2	7	6	11	1	4	5
C10105	10	8	1	5	11	6	9	2	4	7	3
C15512	2	8	11	5	9	3	1	7	4	10	6
C01337	4	2	1	10	9	7	8	5	6	3	11
C02780	1	2	6	8	3	10	9	7	11	4	5
C07610	10	8	5	1	9	6	11	3	2	4	7
C12627	10	9	5	6	1	3	2	8	11	4	7
C04221	11	3	8	2	6	10	1	5	9	7	4
C02214	1	11	5	2	6	3	9	7	8	4	10
C02241	4	9	7	1	2	5	8	3	6	10	11
C06953	4	11	2	5	6	1	9	10	8	7	3
C07618	3	11	9	2	10	7	6	1	4	8	5
C11649	9	2	8	5	1	6	4	3	10	11	7
C10927	11	1	8	10	9	2	3	4	7	6	5
C10861	10	2	8	11	4	3	1	9	6	5	7
C14523	1	3	11	10	9	7	5	6	2	8	4
C14658	3	1	4	5	7	6	2	11	8	10	9
C08386	8	5	11	3	9	7	4	1	2	10	6
C09896	9	1	7	4	11	3	6	10	2	5	8
C01540	9	10	4	2	3	11	8	5	7	1	6
C13880	11	8	3	4	6	10	2	5	1	7	9
C10399	10	7	6	5	1	3	4	9	8	11	2
C14288	11	5	4	9	8	3	6	7	10	2	1
C06911	9	4	5	11	8	1	2	10	3	6	7
C10964	10	5	11	4	3	1	8	2	6	7	9
C01443	1	5	10	6	2	3	4	9	8	11	7
C02661	11	2	8	10	5	1	6	3	7	9	4

C03172	5	2	8	6	3	1	9	4	11	10	7
C06846	10	5	11	7	8	3	9	2	6	4	1
C11367	8	6	2	9	4	3	7	10	11	1	5
C07631	4	11	9	8	2	10	5	1	7	3	6
C14598	3	6	1	5	7	9	10	11	4	2	8
C07889	5	3	10	1	4	2	8	7	11	9	6
C14496	3	2	10	11	5	1	6	4	7	9	8
C11615	9	10	7	2	11	3	4	6	8	5	1
C07674	1	11	5	2	4	9	8	6	10	7	3
C02197	3	11	5	7	9	6	1	2	8	4	10
C11740	5	8	1	10	3	2	11	7	9	4	6
C06765	9	1	7	10	11	8	5	2	6	3	4
C04033	6	2	5	8	3	1	11	9	4	10	7
C07892	9	3	1	4	2	8	11	7	6	5	10
C09201	10	2	5	1	7	9	3	8	6	11	4
C09839	9	11	8	2	10	3	5	4	6	1	7
C01714	10	1	9	4	3	11	5	2	8	7	6
C10068	10	6	4	2	1	9	8	3	5	11	7
C14233	11	5	10	7	8	2	6	3	9	1	4
C08277	5	1	3	2	8	11	4	6	9	7	10
C13846	3	11	8	9	5	10	7	2	4	1	6
C15362	3	2	5	8	6	7	4	10	1	11	9
C08029	9	1	10	11	6	7	2	5	3	8	4
C02233	1	6	5	3	9	2	4	11	10	8	7
C09618	10	3	9	11	1	4	6	5	8	2	7
C11258	2	11	3	1	10	4	7	9	8	5	6
C01018	1	2	5	10	3	6	8	9	4	11	7
C14297	11	5	8	6	3	1	10	4	2	9	7
C01616	2	8	6	5	1	9	11	3	7	4	10
C06869	10	1	9	5	6	2	8	11	4	7	3
C05151	3	10	1	11	4	5	2	6	9	8	7
C10929	3	1	4	7	8	11	6	5	2	10	9
C07886	5	10	4	3	2	1	9	7	6	11	8
C11842	2	5	1	3	7	6	10	8	9	11	4
C10613	5	2	6	8	9	7	10	3	11	1	4
C14624	3	1	8	11	7	6	5	9	2	4	10
C07639	2	5	6	4	1	7	8	3	11	9	10
C08182	4	10	2	11	1	7	6	9	3	8	5
C03953	1	2	7	10	4	5	3	6	8	11	9
C06189	2	1	7	9	5	11	6	3	8	10	4
C12317	5	1	6	2	8	10	9	4	3	11	7
C01558	3	8	2	1	9	6	5	4	11	10	7
C09510	4	6	11	2	10	8	7	5	1	3	9

C04164	1	10	9	4	5	3	11	8	6	2	7
C13804	3	5	6	1	7	2	8	4	11	10	9
C07172	9	11	10	5	3	2	6	1	8	7	4
C14581	11	2	8	4	10	6	3	9	1	5	7
C09316	3	1	9	7	10	11	5	2	8	4	6
C15453	11	4	9	7	8	6	1	3	2	5	10
C09663	9	3	10	1	4	7	2	11	8	6	5
C15339	3	1	2	7	10	6	8	9	11	4	5
C03509	1	2	4	8	5	9	3	7	6	10	11
C04861	1	7	9	4	10	2	11	3	6	5	8
C09311	10	3	11	8	1	5	6	4	2	9	7
C09553	10	2	9	1	11	7	6	3	5	4	8
C14456	11	2	8	6	5	1	4	10	7	3	9
C01239	2	8	5	6	1	11	9	7	3	10	4
C05116	3	1	2	5	11	9	6	8	7	10	4
C06827	10	9	3	6	4	5	8	7	1	11	2
C14499	11	3	1	10	5	4	9	6	7	8	2
C08263	5	1	9	10	4	8	6	11	3	2	7
C08368	3	1	8	11	4	6	10	7	9	2	5
C08749	10	9	5	11	3	4	1	7	8	6	2
C06324	8	2	11	5	4	7	3	1	6	9	10
C03183	5	10	8	6	4	2	3	9	11	7	1
C07651	5	2	6	8	11	9	10	4	7	1	3
C10113	10	8	11	5	2	3	6	9	7	4	1
C09691	9	3	10	11	5	8	2	6	7	1	4
C06943	5	10	11	2	3	8	6	4	1	9	7
C13931	6	8	5	1	2	10	11	4	9	3	7
C09053	10	9	2	7	1	5	8	3	6	4	11
C12979	11	8	5	3	10	9	2	4	1	7	6
C13416	3	11	4	5	2	9	8	10	7	6	1
C10535	10	2	5	4	1	6	3	9	8	7	11
C15296	6	11	5	8	2	1	4	3	10	7	9
C09197	10	5	6	4	2	9	3	11	7	1	8
C11755	10	7	4	9	8	3	6	5	1	2	11
C01608	3	1	10	9	8	5	11	4	6	2	7
C02231	6	2	8	5	9	3	4	11	7	10	1
C13373	1	2	8	6	11	5	4	9	7	3	10
C00305	1	5	8	2	4	6	10	9	11	3	7
C13881	1	5	2	8	6	10	4	9	3	7	11
C10488	10	5	11	2	1	8	6	9	3	7	4
C10901	10	2	11	5	8	6	9	1	7	3	4
C10202	10	7	9	2	8	5	1	3	11	6	4
C10092	3	1	6	2	10	11	5	8	9	4	7

C12177	9	4	5	10	8	2	7	11	3	6	1
C07459	5	2	6	8	7	1	4	10	3	9	11
C11535	1	2	8	11	6	4	9	5	3	10	7
C08322	3	10	1	5	2	8	9	6	4	7	11
C01410	11	1	2	9	10	7	3	6	4	8	5
C08851	1	10	9	5	11	6	4	3	8	2	7
C02648	2	8	6	4	5	3	1	7	10	11	9
C11382	9	11	8	2	10	3	4	7	5	6	1
C03622	2	8	1	11	4	6	7	5	9	3	10
C10500	10	5	1	3	6	2	8	11	4	9	7
C09258	10	11	5	7	4	6	9	8	3	1	2
C09749	9	3	2	1	11	4	10	8	5	7	6
C07139	10	9	5	6	1	4	8	7	2	3	11
C00815	1	5	2	11	6	8	9	3	4	10	7
C10022	10	1	11	7	8	5	3	9	6	2	4
C08318	3	8	6	9	11	7	2	5	1	4	10
C09887	9	10	2	4	3	1	8	11	5	7	6
C01897	10	9	11	4	1	8	3	5	6	7	2
C06770	10	9	11	4	1	2	8	5	3	6	7
C06957	8	3	6	1	9	11	7	2	10	4	5
C07182	1	5	2	6	8	10	4	11	3	9	7
C03521	1	2	8	5	6	4	10	11	3	7	9
C00797	2	5	11	10	6	8	4	1	9	3	7
C04707	3	2	8	6	4	5	7	11	1	10	9
C05344	1	6	8	2	7	10	5	9	4	11	3
C02893	11	10	2	4	5	6	7	9	3	1	8
C02983	5	2	10	8	9	11	3	6	1	4	7
C11252	11	2	7	4	9	8	3	10	5	6	1
C08482	3	9	5	1	10	11	4	6	7	2	8
C08312	5	10	2	8	1	11	9	3	6	7	4
C10913	4	5	11	10	1	8	3	6	7	9	2
C08994	10	5	11	1	8	9	7	2	4	6	3
C08157	3	9	11	1	10	4	2	7	8	6	5
C13838	3	6	5	10	2	9	8	1	11	4	7
C05771	8	2	5	4	11	3	6	9	1	7	10
C07112	11	2	8	5	9	1	3	10	7	6	4
C03404	4	5	2	3	7	8	10	11	6	9	1
C07872	10	11	3	4	6	8	5	9	1	2	7
C07004	3	5	10	2	7	6	1	11	8	4	9
C02532	2	5	6	1	8	3	10	7	11	9	4
C03174	5	2	1	8	11	6	9	7	10	3	4
C06354	11	8	5	9	2	3	4	1	10	6	7
C12226	10	11	4	9	7	8	3	2	1	6	5

C14603	3	4	6	9	11	8	7	2	10	1	5
C01952	5	6	10	4	2	8	1	7	11	9	3
C07364	9	10	11	4	3	8	7	6	2	1	5
C02797	3	11	8	4	2	7	6	9	1	5	10
C11304	10	9	4	6	2	5	7	1	3	8	11
C09791	9	11	6	5	7	2	3	8	10	1	4
C07627	8	1	6	2	5	11	10	9	3	7	4
C11196	11	10	9	2	5	8	7	1	6	3	4
C13392	11	10	9	3	5	6	7	1	8	2	4
C02426	1	2	5	8	6	3	9	11	10	4	7
C10307	11	4	2	3	5	10	9	1	6	7	8
C03861	2	8	11	4	3	1	6	10	5	9	7
C08507	11	6	2	10	8	1	4	7	3	5	9
C06984	11	9	10	1	8	5	4	3	7	6	2
C07612	3	1	11	10	7	2	9	6	5	8	4
C07375	10	5	11	2	3	1	8	6	9	4	7
C14618	3	4	10	6	5	2	9	1	11	8	7
C08942	1	3	8	5	11	9	7	2	4	10	6
C11170	10	9	6	5	8	4	2	3	7	1	11
C09647	9	10	3	2	5	6	7	11	1	4	8
C08356	1	2	5	6	8	3	10	11	9	4	7
C11156	11	3	5	10	8	4	2	6	9	1	7
C01047	2	10	6	8	9	4	5	1	7	11	3
C10965	11	6	10	5	1	8	3	7	9	2	4
C07144	5	10	2	11	7	6	8	3	9	1	4
C03408	1	9	7	4	11	10	2	5	6	3	8
C10390	11	10	8	5	2	6	1	9	3	7	4
C14232	10	5	9	1	7	8	2	3	6	11	4
C12165	10	9	8	1	2	3	7	4	5	11	6
C09391	10	9	8	6	11	5	2	3	4	1	7
C09309	10	1	9	2	5	4	8	6	3	11	7
C10304	10	5	7	1	8	2	6	4	3	9	11
C03600	1	11	8	5	3	2	9	4	7	6	10
C03623	8	6	2	1	5	7	10	4	3	11	9
C11778	10	11	1	7	5	3	8	6	9	4	2
C07967	5	11	1	10	4	6	7	2	3	9	8
C14569	2	5	4	11	8	3	1	7	6	9	10
C07153	10	11	5	2	1	9	3	7	6	8	4
C06068	10	5	11	9	4	1	3	2	6	7	8
C11141	10	1	7	5	11	3	9	2	6	4	8
C10047	10	2	7	1	4	9	11	8	6	3	5
C08719	10	9	6	1	2	4	5	11	3	8	7
C08816	9	3	6	10	2	7	8	5	11	4	1

C14224	11	3	1	10	8	5	4	9	2	6	7
C13706	1	2	5	6	11	8	4	7	10	9	3
C02660	9	6	11	5	8	10	4	3	7	2	1
C12443	9	10	4	1	11	5	7	6	3	2	8
C10873	3	5	9	2	6	10	7	4	11	1	8
C09728	10	5	11	8	2	6	1	3	9	4	7
C00841	2	8	5	6	10	9	3	11	7	1	4
C12599	4	11	9	2	1	8	6	5	3	7	10
C09982	10	2	1	11	4	3	6	9	7	8	5
C06802	1	5	6	2	10	8	3	7	4	9	11
C05425	3	8	9	5	10	6	11	2	4	7	1
C14471	11	6	2	4	10	1	7	3	8	5	9
C10374	11	5	1	9	8	4	2	3	10	6	7
C15689	11	8	10	5	9	6	4	3	7	1	2
C07449	5	10	3	6	4	2	1	7	11	9	8
C07778	5	3	9	11	10	8	4	6	1	7	2
C01349	3	10	5	6	4	9	2	11	7	1	8
C06726	8	2	9	1	4	10	6	3	11	5	7
C09654	10	9	11	6	4	5	8	3	7	2	1
C14434	11	10	1	3	5	6	7	8	4	9	2
C09350	9	11	3	7	4	8	2	1	10	5	6
C10676	10	3	9	11	5	2	4	8	1	7	6
C09271	1	10	5	6	9	2	4	7	3	8	11
C03124	2	5	6	4	7	3	8	9	1	10	11
C11359	10	8	5	1	9	3	4	2	7	6	11
C02569	9	2	8	3	5	4	11	1	7	10	6
C08117	10	6	5	11	9	3	1	8	7	2	4
C08148	1	3	11	5	9	6	10	2	8	4	7
C07774	5	10	3	11	9	7	6	4	2	8	1
C10805	11	5	9	1	2	10	3	4	8	7	6
C09465	10	6	11	9	7	8	2	1	3	4	5
C02077	5	2	1	11	6	8	10	3	4	7	9
C04692	5	9	11	7	10	2	6	3	1	4	8
C09589	10	7	5	8	3	6	11	4	9	2	1
C15752	2	5	7	8	3	6	1	4	10	9	11
C00758	2	8	5	6	9	10	11	7	3	1	4
C10663	10	5	3	7	9	8	6	11	1	2	4
C14355	11	8	2	4	3	7	10	1	9	6	5
C11106	11	2	9	1	10	8	6	3	5	7	4
C08470	9	11	1	2	6	4	10	3	8	7	5
C07320	3	1	5	9	11	10	8	6	7	2	4
C03137	5	3	10	8	11	2	9	6	1	4	7
C13888	8	1	9	6	2	4	10	3	11	5	7

C09523	8	7	6	5	2	1	4	11	3	10	9
C09005	11	4	1	8	6	7	5	9	10	3	2
C09885	9	11	10	5	1	4	8	7	3	6	2
C03184	8	2	5	11	7	1	3	4	6	10	9
C02692	1	2	6	8	10	3	11	5	4	9	7
C14437	5	11	3	10	9	1	6	8	7	2	4
C07504	11	5	8	10	6	9	1	4	2	7	3
C13929	11	7	8	9	1	5	3	4	10	2	6
C14539	9	10	7	11	8	5	2	1	3	4	6
C08309	5	10	11	1	4	9	6	2	3	8	7
C08419	10	5	6	1	3	2	8	9	11	7	4
C06471	1	9	7	10	2	6	4	8	11	3	5
C12119	5	8	6	9	7	2	4	10	1	3	11
C10579	9	3	11	5	1	8	2	4	10	7	6
C02903	6	5	8	2	1	10	11	4	9	7	3
C06244	1	2	11	6	5	3	4	9	10	7	8
C03994	11	5	10	6	4	7	3	8	1	9	2
C11786	11	10	4	9	3	8	1	5	7	6	2
C13938	10	3	9	7	5	4	11	6	2	1	8
C10586	10	5	11	6	2	1	9	7	3	8	4
C08053	10	9	5	2	11	8	4	7	3	1	6
C06887	10	9	1	2	6	5	11	4	8	3	7
C08027	10	8	11	1	5	2	9	6	3	4	7
C14728	3	11	10	4	9	8	1	7	6	5	2
C14406	11	8	10	2	5	4	1	6	3	7	9
C03068	11	2	8	3	6	10	1	4	5	9	7
C10238	10	11	1	5	3	4	9	6	7	8	2
C09457	10	11	3	2	1	7	8	9	4	6	5
C11705	3	5	11	9	1	7	2	8	10	4	6
C11158	9	8	11	10	4	3	2	5	6	1	7
C07345	8	2	5	3	6	1	11	10	4	9	7
C07708	1	5	4	7	9	2	11	6	3	10	8
C14763	10	6	4	3	1	5	9	2	7	11	8
C10241	11	1	9	8	3	6	5	10	7	2	4
C14512	11	10	5	1	8	2	3	9	6	4	7
C14381	11	5	2	10	6	8	1	7	9	3	4
C12303	9	5	11	3	2	6	1	7	10	8	4
C06477	1	2	11	3	8	9	4	5	10	6	7
C10031	10	5	11	8	6	7	2	3	1	9	4
C08608	2	8	5	9	11	6	1	7	3	10	4
C08432	4	10	9	6	11	1	3	2	5	8	7
C10961	11	6	2	10	1	8	4	5	3	9	7
C13854	9	3	11	5	4	10	1	7	2	6	8

C10271	10	2	11	9	6	8	3	7	4	5	1
C08124	10	9	2	4	7	3	8	6	11	1	5
C16463	4	1	3	9	8	10	6	5	11	7	2
C14235	11	2	7	5	9	3	4	6	1	10	8
C07833	11	10	3	9	5	8	2	7	1	6	4
C01740	11	2	6	3	5	4	8	10	1	9	7
C09313	10	3	11	6	5	1	4	8	7	2	9
C02735	5	11	10	9	3	8	1	2	7	6	4
C14567	9	5	10	3	11	8	7	4	2	1	6
C03996	8	1	9	4	3	10	7	5	2	11	6
C12057	10	2	1	8	3	5	6	7	11	9	4
C11033	5	2	3	6	10	7	11	8	9	4	1
C15337	3	9	10	5	1	8	11	2	4	6	7
C00982	2	4	8	7	1	5	11	6	3	10	9
C06513	10	7	11	1	3	4	9	2	5	8	6
C08998	4	11	1	6	2	5	10	9	3	7	8
C06496	3	11	6	10	4	9	5	2	8	1	7
C11698	5	11	7	4	8	3	10	2	6	9	1
C12035	7	6	4	5	11	2	8	10	3	1	9
C08385	3	1	8	5	7	11	2	9	4	10	6
C06108	1	11	2	5	6	8	9	4	10	3	7
C02555	4	2	7	8	10	9	5	3	1	6	11
C05529	1	2	5	6	8	11	9	4	10	3	7
C00318	5	3	8	1	2	6	11	9	4	10	7
C07508	10	5	1	4	6	8	2	3	11	7	9
C00774	10	1	3	2	5	11	8	6	9	4	7
C15552	1	3	10	4	6	9	5	8	11	2	7
C08342	1	6	10	11	8	3	7	4	2	5	9
C11844	5	2	10	9	7	3	1	4	8	6	11
C11674	1	3	9	5	4	11	6	2	10	8	7
C07235	9	8	5	6	10	1	11	4	2	7	3
C11219	3	11	8	2	5	10	7	4	6	1	9
C08033	1	9	7	10	2	4	6	3	5	11	8
C07789	10	5	3	9	11	6	4	1	2	8	7
C08984	8	9	11	10	7	6	3	2	1	4	5
C15494	2	8	5	11	1	10	3	4	6	7	9
C14590	3	9	8	4	5	11	2	7	1	6	10
C10912	9	11	2	10	7	1	8	3	6	4	5
C09112	3	1	10	11	6	5	8	4	9	7	2
C08980	3	1	10	11	8	4	2	5	9	7	6
C09550	10	5	6	3	1	7	2	9	8	4	11
C09925	10	2	5	8	1	3	9	6	7	11	4
C08364	3	10	1	5	9	11	2	7	8	6	4

C07851	10	3	11	4	7	2	9	1	5	8	6
C10823	5	1	11	7	6	3	10	9	4	8	2
C11086	11	6	2	5	8	4	10	1	3	9	7
C03712	6	5	3	8	11	1	7	2	4	10	9
C06257	9	8	10	2	1	4	5	3	11	6	7
C12202	9	10	8	4	7	5	3	11	6	2	1
C07852	1	10	7	8	9	5	4	11	2	3	6
C07373	8	3	5	9	10	6	1	11	2	4	7
C16439	5	6	1	2	8	10	4	3	11	9	7
C14716	11	5	2	9	7	10	3	8	1	4	6
C10619	9	11	10	7	4	3	8	5	6	1	2
C01135	1	2	4	9	10	8	3	7	6	5	11
C03238	3	6	2	9	8	5	1	4	11	10	7
C11798	5	3	10	1	6	4	2	11	8	9	7
C09796	10	9	11	1	5	4	2	3	7	8	6
C14228	11	2	10	8	9	5	1	3	4	7	6
C02979	1	2	3	7	8	10	11	9	5	6	4
C08376	3	5	6	8	4	7	11	10	2	9	1
C01495	10	1	5	11	8	9	3	2	4	6	7
C01569	2	8	1	11	4	9	6	10	7	3	5
C14292	9	11	5	10	3	8	6	7	1	2	4
C10341	10	11	5	1	9	6	3	4	2	8	7
C11362	5	2	8	4	10	6	3	11	1	9	7
C10511	10	9	8	7	3	1	2	5	11	6	4
C08279	5	11	1	9	10	8	3	6	4	7	2
C07660	10	9	4	11	5	2	6	7	1	8	3
C03884	5	3	6	10	2	4	8	9	1	11	7
C10421	10	9	11	8	1	3	5	7	2	6	4
C00772	8	9	3	2	6	1	11	5	10	7	4
C08834	9	3	6	2	4	11	8	7	10	5	1
C03524	3	1	2	11	6	9	5	8	7	4	10
C07420	5	6	10	1	9	4	2	11	8	3	7
C09763	10	2	7	5	6	3	4	8	9	11	1
C02150	2	11	1	6	3	10	5	7	9	8	4
C00220	3	1	8	2	4	11	5	7	9	10	6
C09278	2	8	6	10	3	1	5	9	11	4	7
C14474	9	10	4	1	11	5	3	8	6	2	7
C04275	1	10	6	4	7	2	5	3	11	8	9
C03750	2	6	8	4	5	10	1	3	11	7	9
C14468	3	11	5	2	4	9	8	6	1	10	7
C08136	1	3	11	2	4	10	5	7	9	8	6
C03204	2	8	4	9	5	10	11	1	7	6	3
C14442	11	10	9	2	3	1	6	5	8	7	4

C14472	1	11	2	10	7	8	4	5	3	6	9
C02788	3	2	6	8	7	1	9	5	11	4	10
C14331	11	5	1	10	3	6	8	2	4	9	7
C12290	9	11	5	1	3	6	2	10	7	8	4
C02088	10	9	5	2	11	6	3	8	1	4	7
C04187	2	4	9	10	6	5	3	8	1	11	7
C09440	3	1	4	7	5	11	6	2	8	10	9
C07269	5	3	2	10	6	4	8	1	11	9	7
C09314	8	3	5	11	1	2	4	9	6	7	10
C08836	3	10	9	1	8	11	2	5	4	6	7
C08336	6	1	3	4	5	7	11	10	2	9	8
C07332	1	11	5	10	7	4	3	9	8	2	6
C11007	11	3	6	2	10	9	7	4	1	8	5
C10914	11	1	5	10	6	7	3	4	9	2	8
C05462	3	8	2	4	7	6	5	1	9	10	11
C12059	1	11	5	2	10	8	3	4	9	6	7
C08823	3	5	6	10	11	4	1	2	7	8	9
C05347	2	1	6	5	11	7	8	10	4	3	9
C11279	3	1	6	11	2	5	8	10	4	9	7
C14509	9	11	3	1	5	10	6	2	8	7	4
C12829	11	5	4	9	10	1	3	6	7	2	8
C05372	3	10	5	4	2	6	1	11	8	7	9
C05597	5	6	2	11	1	8	4	3	9	10	7
C10575	5	10	1	8	4	9	3	2	11	6	7
C10932	4	2	5	10	7	3	11	9	6	1	8
C11317	3	1	11	10	2	9	5	8	4	6	7
C08100	10	1	9	2	5	7	6	8	3	4	11
C03072	3	8	9	10	2	6	5	1	4	11	7
C15186	3	7	5	11	1	9	4	10	2	8	6
C14007	10	9	8	6	5	7	11	1	3	2	4
C09591	10	11	5	7	6	2	3	8	4	1	9
C13662	3	1	5	10	8	4	6	7	11	9	2
C05386	1	2	4	8	9	10	6	7	3	11	5
C01722	1	2	5	8	6	10	3	11	9	4	7
C06697	2	6	8	1	5	9	4	11	10	7	3
C14229	11	6	1	5	10	2	8	4	3	7	9
C11387	9	11	10	1	8	3	6	7	5	2	4
C01601	3	11	1	9	5	8	6	10	2	4	7
C10780	10	4	8	5	11	2	1	7	6	3	9
C00557	2	8	3	11	6	5	1	10	9	4	7
C08568	10	5	8	11	2	4	1	6	3	9	7
C14201	11	5	3	8	9	4	1	6	10	2	7
C08512	11	4	3	7	2	10	6	1	8	5	9

C10464	10	2	6	11	9	3	5	4	1	7	8
C13749	10	5	2	4	11	8	6	9	7	1	3
C15419	11	5	1	7	10	3	4	8	2	6	9
C14729	1	5	10	8	11	6	4	9	2	3	7
C10245	10	11	1	9	5	8	3	4	6	7	2
C03195	3	8	2	11	4	10	6	1	7	5	9
C14322	11	10	9	1	2	5	3	6	7	8	4
C10478	9	5	10	11	2	7	1	3	6	8	4
C14317	11	3	9	6	5	1	4	10	8	2	7
C08498	9	11	5	6	3	1	10	4	8	7	2
C12560	2	1	7	11	6	9	4	5	3	10	8
C09835	3	1	2	9	6	7	11	10	4	5	8
C07699	10	3	5	2	11	8	4	7	6	1	9
C10359	10	8	11	7	9	3	5	1	4	6	2
C14582	11	5	9	2	6	4	1	3	8	10	7
C13198	2	8	11	1	3	5	10	4	7	9	6
C13044	3	11	1	6	9	8	2	4	7	5	10
C10235	1	10	9	7	2	3	5	8	6	4	11
C11805	5	1	8	10	6	2	3	4	9	7	11
C07841	11	5	10	3	1	6	4	8	7	9	2
C15695	9	11	6	10	8	5	1	2	7	3	4
C15649	3	9	2	1	8	6	11	7	4	10	5
C06947	10	5	11	8	6	2	1	9	3	4	7
C04645	1	4	9	2	7	3	8	10	6	11	5
C12073	10	11	2	9	5	1	3	6	7	8	4
C06890	10	9	5	6	1	2	3	8	4	11	7
C08899	10	2	9	11	6	3	4	7	8	1	5
C14257	3	6	2	1	10	5	4	8	11	7	9
C12194	10	3	6	7	4	8	11	1	5	2	9
C09419	10	3	5	4	1	11	9	2	8	7	6
C01585	3	1	11	5	2	6	8	9	10	4	7
C09317	10	9	5	11	7	8	2	3	6	4	1
C08066	9	11	5	3	4	2	10	8	1	7	6
C06228	8	9	10	2	11	7	5	4	3	1	6
C11325	10	11	7	5	3	2	9	8	6	4	1
C04503	4	2	9	10	7	11	8	6	1	3	5
C13358	3	2	9	7	5	1	11	10	6	4	8
C01710	11	8	2	3	10	1	6	4	7	9	5
C10931	11	6	5	1	8	4	3	9	10	2	7
C01119	2	1	8	6	5	11	3	9	7	4	10
C10845	10	1	2	6	11	3	8	9	5	7	4
C08663	9	6	11	2	4	7	3	1	8	5	10
C03197	1	5	2	3	11	8	6	9	10	4	7

C09930	5	10	1	7	9	3	8	6	4	2	11
C11155	10	8	11	5	6	1	3	2	9	4	7
C13870	3	11	1	8	5	6	7	10	9	4	2
C13587	1	3	10	5	8	7	11	9	6	2	4
C07272	2	6	8	5	1	11	10	9	4	3	7
C00756	11	2	9	1	5	3	8	10	6	4	7
C10749	10	2	6	4	1	9	11	3	5	8	7
C07051	2	1	7	3	4	6	9	5	8	11	10
C13764	10	5	2	1	4	9	3	11	7	6	8
C10183	10	3	8	1	4	7	11	5	2	6	9
C15591	2	8	11	4	9	6	7	3	10	5	1
C14578	11	4	2	7	1	9	5	3	8	10	6
C15499	1	11	5	2	6	8	3	7	9	4	10
C08402	11	8	6	3	5	9	7	4	2	1	10
C03549	10	1	5	11	8	2	9	3	6	7	4
C02766	4	5	2	11	10	6	1	8	3	9	7
C01956	5	2	8	11	6	4	9	10	3	1	7
C05282	8	11	5	1	3	10	2	4	6	9	7
C11689	10	5	1	9	6	3	8	7	11	2	4
C09345	9	11	4	10	8	2	7	3	5	6	1
C07759	10	11	5	2	1	7	9	3	4	6	8
C01550	11	2	10	5	7	6	4	8	3	1	9
C06266	11	2	10	1	8	5	6	4	9	7	3
C14550	11	3	10	8	5	1	9	4	2	7	6
C04760	3	2	8	9	4	10	7	5	1	6	11
C11370	9	1	8	2	7	11	5	4	3	10	6
C08855	3	2	11	5	6	1	9	4	7	8	10
C03241	2	8	6	11	10	3	1	4	7	5	9
C13852	1	2	6	5	8	10	11	3	7	4	9
C01857	2	5	1	3	9	11	6	8	7	10	4
C08372	5	2	9	11	1	10	6	7	3	4	8
C03268	1	9	4	7	3	8	2	10	5	11	6
C14142	11	1	6	8	4	3	9	7	5	2	10
C08074	3	11	5	9	6	8	4	1	10	2	7
C10332	11	5	8	2	6	9	1	3	7	10	4
C03476	2	8	4	5	1	9	7	10	11	6	3
C10800	11	9	5	7	1	10	6	3	8	4	2
C00806	5	6	2	8	10	1	9	11	4	3	7
C14623	4	11	9	5	1	8	6	2	3	7	10
C14641	3	1	8	9	6	7	4	11	5	2	10
C10994	11	9	10	4	8	3	6	1	7	2	5
C10051	10	11	5	9	7	2	3	1	8	6	4
C07074	9	3	1	8	5	10	2	11	6	7	4

C12085	5	10	6	1	3	8	11	9	2	4	7
C08677	9	10	2	1	6	3	8	11	4	7	5
C09892	10	4	7	1	11	6	5	8	3	9	2
C02345	10	5	11	2	8	3	4	1	7	6	9
C13168	10	5	1	3	6	8	2	11	9	4	7
C07537	11	10	3	8	2	9	6	1	4	5	7
C02914	2	1	5	11	4	8	6	10	3	7	9
C12540	9	10	1	2	5	11	3	7	6	8	4
C15497	8	4	2	9	11	6	7	3	1	5	10
C07597	8	9	1	11	4	2	7	5	3	10	6
C07402	11	9	10	4	3	8	5	6	7	1	2
C03503	9	1	4	3	2	11	6	5	7	8	10
C07372	5	11	8	1	10	6	2	3	4	9	7
C01906	2	9	4	1	10	8	11	6	5	3	7
C14187	11	3	10	1	9	4	2	7	8	5	6
C11201	10	9	11	8	6	3	7	2	5	1	4
C14428	11	6	9	2	5	4	8	10	1	3	7
C14312	11	2	3	1	10	5	7	4	6	8	9
C10577	6	1	5	2	8	3	9	11	4	10	7
C01075	1	2	4	5	8	7	10	11	3	9	6
C04273	1	10	6	2	8	11	4	3	7	9	5
C13964	1	10	6	9	2	7	5	11	3	8	4
C11469	9	6	11	8	5	4	10	2	1	7	3
C08854	3	2	11	9	1	6	5	7	8	4	10
C01310	2	5	4	8	9	1	6	3	11	7	10
C07467	1	5	11	3	4	9	8	10	7	6	2
C09426	10	7	3	11	2	8	4	6	1	9	5
C04015	1	3	5	2	9	11	6	8	7	10	4
C06825	1	10	3	5	2	8	9	11	4	7	6
C07407	10	5	3	1	4	9	11	8	2	6	7
C02048	1	10	2	9	7	8	6	11	3	5	4
C14183	1	2	4	5	11	10	9	7	6	8	3
C10979	3	5	2	6	11	4	7	1	8	9	10
C07006	3	1	10	4	6	8	2	7	9	11	5
C13735	5	6	8	10	2	1	3	9	11	4	7
C01630	1	11	9	10	4	8	2	7	5	3	6
C00563	1	10	3	5	2	11	4	6	9	8	7
C10699	10	2	4	1	8	6	9	5	7	3	11
C09528	6	1	5	8	4	11	2	7	10	9	3
C06075	9	8	3	2	11	10	1	5	6	7	4
C08073	11	5	3	10	9	1	2	6	4	8	7
C03031	4	7	8	2	3	5	9	6	10	1	11
C08499	9	1	5	3	11	2	6	10	4	7	8

C14416	11	10	5	2	1	8	9	6	3	7	4
C03243	11	3	8	5	1	2	6	10	4	9	7
C11011	11	3	9	8	4	2	10	6	1	7	5
C10063	1	2	11	9	8	6	3	7	5	4	10
C03829	5	6	2	1	8	4	10	9	11	7	3
C07531	11	10	5	2	9	3	6	7	4	8	1
C08628	10	3	11	9	1	2	5	8	6	7	4
C11720	3	2	5	8	6	4	9	7	11	10	1
C03142	4	1	9	3	8	5	2	6	7	11	10
C10541	10	3	5	11	8	6	2	9	1	4	7
C08727	10	1	9	11	5	2	7	8	6	4	3
C11010	11	1	8	5	9	4	3	6	2	10	7
C10410	10	2	11	3	9	7	1	6	4	5	8
C03413	5	6	10	11	8	4	2	1	7	3	9
C11314	11	1	2	5	10	9	4	3	8	6	7
C09473	10	5	9	6	3	2	8	7	1	4	11
C12063	1	10	7	9	4	2	3	6	5	11	8
C03844	1	10	2	5	8	6	9	3	11	7	4
C08947	1	3	5	6	11	9	8	7	2	4	10
C00764	1	2	5	6	8	3	10	11	9	4	7
C02126	11	2	5	8	10	6	4	1	3	9	7
C12896	5	3	1	9	2	8	4	10	6	11	7
C09697	10	5	11	7	3	6	8	1	4	9	2
C10494	11	10	8	9	4	5	3	6	7	2	1
C09541	10	11	8	1	7	3	6	9	4	2	5
C08193	6	5	2	1	11	8	9	10	7	3	4
C03274	3	8	1	7	9	5	11	10	4	6	2
C08311	5	10	1	6	3	8	2	11	4	9	7
C06238	1	4	9	2	10	3	7	8	6	5	11
C15216	3	11	5	4	1	6	8	2	10	9	7
C09237	10	7	5	6	11	3	9	8	1	4	2
C07251	10	11	5	2	4	1	6	9	7	3	8
C05149	3	9	10	2	4	8	1	7	5	6	11
C12853	9	2	5	4	10	6	8	3	11	1	7
C09861	9	2	11	1	6	8	3	5	7	10	4
C09289	9	10	6	2	1	4	11	3	8	7	5
C09279	10	3	1	2	7	8	6	5	11	4	9
C08603	9	8	6	10	5	4	1	7	3	11	2
C10325	9	11	7	6	5	8	2	3	4	10	1
C07151	3	1	5	4	2	9	8	11	10	6	7
C10465	8	5	11	10	7	1	3	2	4	6	9
C04621	3	11	8	10	9	5	2	6	4	1	7
C10989	11	1	2	6	5	10	8	4	9	7	3

C07414	5	10	11	6	1	3	8	2	9	7	4
C14655	3	4	7	1	2	5	11	9	10	6	8
C08775	1	10	3	11	2	4	9	8	5	6	7
C11772	5	10	11	4	3	8	6	1	7	9	2
C08505	2	1	8	7	11	3	9	6	4	5	10
C00076	1	5	3	2	8	4	6	10	11	9	7
C04864	2	4	8	5	1	9	7	3	11	6	10
C13427	11	8	9	5	1	3	4	10	6	7	2
C11339	2	1	6	5	10	11	4	8	7	9	3
C12012	9	10	11	4	6	3	5	8	1	2	7
C09743	9	1	5	6	4	8	11	3	2	10	7
C01553	11	10	9	4	6	2	7	5	3	8	1
C07591	10	11	3	8	5	9	2	6	7	1	4
C11265	4	5	11	9	1	10	6	7	3	2	8
C15640	9	1	6	11	8	2	4	3	7	5	10
C14200	11	3	5	10	8	7	1	6	4	9	2
C09386	11	10	2	1	3	5	9	8	7	4	6
C07586	3	1	5	8	10	9	2	4	6	7	11
C07199	5	10	3	8	11	6	1	2	4	9	7
C09504	10	5	9	6	3	2	8	7	1	4	11
C11274	2	11	10	4	5	1	6	9	8	7	3
C03047	5	6	8	10	2	4	11	1	3	7	9
C14530	11	1	2	3	7	5	9	6	10	8	4
C09015	3	5	10	7	6	2	11	1	9	4	8
C08717	10	1	4	6	11	3	8	9	2	7	5
C14514	11	8	7	5	4	3	1	2	10	6	9
C09134	10	11	9	3	1	6	8	4	5	2	7
C11319	11	5	9	7	1	2	3	10	4	6	8
C12225	11	2	6	5	8	3	4	10	9	1	7
C04379	2	8	6	5	11	9	3	4	1	10	7
C15416	3	6	2	10	8	7	11	5	9	4	1
C00742	1	2	11	6	8	5	3	4	10	7	9
C02064	3	6	1	2	5	9	11	4	8	10	7
C14408	11	1	2	4	9	10	6	7	3	8	5
C10109	1	9	11	6	7	10	2	3	5	4	8
C11229	2	3	10	6	8	4	1	11	5	7	9
C04208	5	9	10	11	8	2	6	4	7	3	1
C11234	9	10	1	2	8	7	6	5	3	4	11
C02854	2	6	11	1	4	3	8	7	5	9	10
C15306	3	1	8	7	11	6	4	9	2	5	10
C15341	11	6	9	1	2	7	8	5	3	4	10
C12182	10	4	9	5	6	2	3	7	8	11	1
C13827	11	1	3	2	10	5	6	9	4	8	7

C00284	1	2	8	11	5	6	9	3	10	4	7
C10941	11	6	2	8	7	1	9	5	3	10	4
C03840	11	8	5	2	1	6	4	10	3	7	9
C06764	2	3	6	5	4	9	10	11	1	8	7
C11194	2	11	4	6	3	9	5	7	1	10	8
C14175	11	9	1	10	2	4	5	7	6	3	8
C14421	11	4	8	2	1	10	5	3	6	9	7
C10963	11	6	4	8	1	3	10	7	9	5	2
C14686	5	11	6	3	10	1	4	2	7	8	9
C12919	3	4	5	11	9	7	6	2	1	10	8
C02502	8	2	11	10	9	6	4	1	5	7	3
C06692	11	8	1	4	5	3	6	7	9	2	10
C11019	11	6	5	1	8	10	3	2	4	9	7
C02081	9	8	5	10	11	2	4	3	6	1	7
C16434	5	6	2	1	8	10	4	9	11	3	7
C10922	2	1	8	6	5	10	4	7	3	9	11
C06930	3	8	10	2	4	9	1	11	6	5	7
C08383	9	1	5	10	11	7	2	8	3	6	4
C06916	3	11	8	5	9	1	6	2	10	7	4
C11094	6	5	8	1	4	9	10	2	11	7	3
C10962	8	11	9	3	2	5	7	4	1	10	6
C05370	11	3	5	2	10	1	4	7	9	8	6
C04684	11	2	5	7	10	4	9	8	3	6	1
C14759	11	10	2	1	5	8	3	6	4	9	7
C09873	9	3	11	2	8	1	5	6	7	10	4
C11499	2	8	1	5	6	7	11	4	9	3	10
C12104	11	8	1	9	6	3	2	5	7	10	4
C13668	5	6	8	10	2	1	11	4	3	9	7
C15932	9	8	5	4	2	10	1	11	3	6	7
C02420	6	2	8	1	9	4	11	10	3	5	7
C02084	1	2	5	8	6	11	10	4	9	7	3
C03789	1	2	5	8	10	3	4	9	11	7	6
C13759	3	9	6	4	7	1	11	2	10	8	5
C11503	5	11	10	3	8	6	4	1	2	9	7
C10024	3	5	2	8	7	1	11	9	6	4	10
C09488	7	6	1	9	2	10	8	5	3	11	4
C10343	10	2	8	6	1	3	9	11	5	7	4
C12218	2	8	1	11	5	6	10	4	9	7	3
C08556	10	5	7	11	4	9	3	6	2	8	1
C06747	1	3	10	11	5	2	8	6	7	9	4
C08806	6	11	10	1	4	5	7	3	2	9	8
C15742	5	10	8	6	1	2	9	11	3	4	7
C10920	11	1	4	3	9	5	10	6	8	2	7

C16014	11	8	2	5	3	1	4	9	10	7	6
C03200	1	10	5	2	6	11	8	3	7	4	9
C14268	5	11	10	2	1	9	3	8	4	7	6
C10955	11	8	5	1	6	10	9	2	4	7	3
C13821	3	11	8	10	5	7	9	2	6	1	4
C08040	1	5	6	2	8	11	7	9	4	10	3
C07768	3	10	5	2	8	7	4	1	9	11	6
C10048	10	11	6	1	9	4	7	5	2	3	8
C08862	3	6	2	4	10	7	11	8	1	5	9
C14916	11	5	3	10	8	2	6	7	4	1	9
C10505	8	11	7	1	9	3	10	4	6	5	2
C13722	1	10	4	7	5	2	3	6	9	8	11
C13014	1	11	9	10	5	2	6	7	3	8	4
C09047	11	2	10	7	3	5	8	1	4	6	9
C06472	1	2	11	3	8	9	4	5	10	6	7
C13751	2	3	5	4	8	7	11	6	10	1	9
C11765	6	10	11	8	7	1	5	9	2	3	4
C08895	3	1	4	7	10	8	6	11	2	9	5
C10513	10	5	11	9	2	8	3	7	6	1	4
C10879	3	5	11	6	2	1	7	4	10	9	8
C07606	10	11	8	5	9	2	4	6	3	1	7
C00023	1	11	5	8	2	10	6	4	9	3	7
C02445	1	8	2	5	11	3	4	7	9	6	10
C00080	5	1	10	11	8	9	3	2	4	6	7
C13697	10	1	2	5	11	4	9	3	8	6	7
C10302	8	11	5	10	4	2	3	6	1	7	9
C02278	9	3	1	8	2	10	6	11	5	4	7
C10947	11	5	9	10	3	2	8	1	7	4	6
C03961	5	2	6	8	4	1	11	10	3	9	7
C02994	1	2	8	6	7	10	11	4	5	3	9
C02940	3	11	2	6	1	10	5	9	8	4	7
C03146	1	2	8	9	10	4	6	3	11	5	7
C09603	3	1	8	9	4	11	7	5	2	10	6
C14705	11	5	4	2	7	8	6	10	3	1	9
C10817	1	2	7	6	9	11	3	5	8	10	4
C10140	10	9	4	2	6	7	5	3	11	8	1
C11012	3	10	7	11	9	4	1	5	2	8	6
C04771	1	9	4	5	10	11	6	3	7	8	2
C07347	2	8	5	1	11	7	3	4	10	9	6
C14131	10	3	1	11	5	8	9	7	2	4	6
C06546	5	10	11	7	9	6	2	3	8	4	1
C14403	11	5	1	10	8	2	9	7	6	4	3
C07231	10	9	5	6	11	1	3	8	7	2	4

C03494	1	9	8	2	5	7	11	10	4	3	6
C11206	11	8	5	10	1	6	4	7	3	2	9
C10999	11	8	5	1	4	3	9	2	7	10	6
C08610	9	8	3	5	4	6	1	11	2	7	10
C06349	10	11	9	5	2	1	3	7	6	8	4
C14681	3	5	4	7	1	8	11	9	6	10	2
C01892	2	8	5	10	11	9	1	6	7	3	4
C15990	1	2	10	5	4	7	8	6	9	3	11
C13748	10	11	4	6	5	3	2	1	9	7	8
C08306	5	10	6	11	2	1	4	9	7	3	8
C00225	2	8	11	6	5	1	10	9	3	4	7
C10693	11	10	7	6	4	5	1	3	9	8	2
C09066	10	3	9	5	11	6	2	7	1	4	8
C10953	11	6	5	10	8	2	9	1	3	7	4
C07488	10	5	8	2	9	3	4	1	6	11	7
C01591	1	10	2	5	11	6	3	9	7	4	8
C03782	6	5	1	2	7	3	11	9	8	10	4
C03588	2	6	8	3	11	4	10	7	5	9	1
C08265	2	8	3	5	9	4	6	11	10	1	7
C02129	8	2	3	11	5	6	7	1	10	4	9
C13903	3	5	6	11	1	9	2	10	4	7	8
C01895	5	7	10	9	11	3	2	8	4	6	1
C02868	8	2	11	9	10	6	7	1	5	3	4
C06304	9	11	2	8	5	1	6	7	3	4	10
C12022	4	2	5	3	1	9	7	10	8	11	6
C05551	10	8	5	9	6	3	11	1	2	7	4
C11174	1	2	4	10	7	6	5	8	9	11	3
C08460	11	9	2	8	1	5	3	7	6	4	10
C01750	10	11	1	5	3	2	9	8	6	4	7
C14296	11	8	5	3	10	6	9	4	1	2	7
C03466	9	5	8	10	7	11	4	2	3	1	6
C01341	9	5	3	11	2	8	1	6	4	7	10
C07210	4	11	9	5	2	6	8	3	10	1	7
C10515	10	9	11	5	8	1	3	6	7	2	4
C07865	10	4	5	9	8	1	7	2	3	11	6
C02018	3	2	7	4	10	1	11	5	6	8	9
C15857	9	8	2	6	5	7	10	1	3	11	4
C11819	10	3	5	4	2	9	6	1	8	11	7
C07412	11	9	4	6	5	2	8	3	1	10	7
C03647	11	5	1	8	2	6	4	10	9	7	3
C10633	10	1	2	5	6	3	7	9	11	8	4
C11728	1	9	8	10	11	5	4	7	3	6	2
C08352	1	2	5	10	3	6	8	9	4	11	7

C06348	10	11	9	5	8	2	3	4	6	1	7
C14304	9	10	3	1	6	8	5	2	11	4	7
C11598	2	6	11	8	5	1	9	7	4	10	3
C16456	5	11	1	8	4	7	2	3	9	6	10
C12811	3	11	7	2	9	5	4	1	6	8	10
C02284	10	3	1	2	6	11	5	8	7	4	9
C07952	5	10	8	11	6	4	1	2	9	3	7
C04210	1	5	6	10	8	7	11	4	3	9	2
C05374	1	2	7	10	4	5	3	8	6	11	9
C09600	10	7	2	8	9	6	3	4	5	11	1
C15377	3	5	7	8	11	4	10	1	9	2	6
C02006	9	2	7	10	1	3	5	4	8	6	11
C14337	11	10	9	6	2	5	1	4	3	8	7
C01475	5	6	2	8	10	1	11	4	7	9	3
C13129	10	11	1	7	8	9	2	6	5	3	4
C10695	8	2	5	9	10	11	3	1	6	4	7
C07928	11	5	6	8	2	3	4	7	10	9	1
C11762	1	10	4	5	7	6	2	8	3	11	9
C14816	2	10	5	9	4	7	3	8	6	11	1
C10270	3	4	7	2	9	8	11	10	1	5	6
C14308	9	8	3	1	11	2	4	7	6	10	5
C01968	1	9	7	10	4	3	2	6	5	11	8
C02272	11	5	2	8	1	7	3	4	6	9	10
C13762	10	3	2	5	8	1	6	11	9	4	7
C09032	10	5	3	9	1	2	4	11	7	6	8
C08391	5	2	6	8	7	9	4	3	10	1	11
C07767	4	1	11	5	2	8	3	6	7	10	9
C14547	3	11	7	9	4	8	6	5	1	10	2
C02628	10	6	4	9	1	8	3	5	2	7	11
C07267	10	3	5	1	6	8	2	11	4	9	7
C03827	8	2	9	5	1	11	6	10	3	7	4
C06946	5	10	11	3	9	1	8	2	4	6	7
C14525	11	9	7	10	5	3	4	6	8	2	1
C06733	8	2	11	4	3	5	10	6	9	1	7
C06809	1	5	6	11	8	2	10	9	4	3	7
C14225	11	3	5	8	9	2	4	7	1	6	10
C08010	11	10	5	4	7	3	1	8	6	2	9
C14396	11	9	1	5	10	7	8	3	2	6	4
C11111	3	9	6	8	4	2	11	1	7	10	5
C03293	10	2	5	11	9	4	1	6	7	3	8
C08841	10	1	4	9	5	3	11	7	2	8	6
C07949	5	11	10	1	8	9	7	2	3	4	6
C02712	5	1	8	2	10	6	4	11	9	7	3

C09277	10	9	7	5	1	3	6	2	4	8	11
C06906	5	10	8	11	6	4	1	2	9	7	3
C12201	10	9	7	1	11	3	5	8	4	6	2
C10921	11	1	8	2	6	3	10	9	7	5	4
C07555	11	8	2	1	6	3	5	9	4	10	7
C09159	9	3	2	10	8	11	5	6	1	7	4
C01518	1	10	2	9	7	8	6	11	3	5	4
C09077	10	11	9	6	4	2	8	1	3	7	5
C01319	1	2	5	9	8	6	11	10	4	3	7
C11142	2	8	5	6	11	10	3	4	9	7	1
C04378	10	8	11	3	1	5	2	9	6	4	7
C06358	9	5	10	11	8	1	6	3	2	4	7
C08183	3	11	8	2	1	9	5	4	10	7	6
C01007	8	2	6	10	11	1	5	7	4	3	9
C02549	2	8	10	1	6	4	3	5	7	9	11
C06857	5	10	11	1	8	2	6	4	7	3	9
C02223	5	2	11	1	9	10	6	8	4	3	7
C02747	2	8	6	7	10	3	1	11	4	9	5
C09017	9	11	10	6	4	5	1	3	2	8	7
C09801	11	10	9	5	1	8	4	7	3	6	2
C01819	5	10	8	11	6	2	9	1	7	3	4
C15531	5	10	9	7	3	8	11	6	1	2	4
C15569	8	2	9	6	5	10	7	3	1	11	4
C08039	11	5	1	4	9	6	8	3	2	10	7
C11734	5	11	10	8	3	6	7	2	9	1	4
C08110	10	9	6	4	2	11	7	3	1	8	5
C11766	10	11	1	5	2	8	3	4	6	7	9
C10815	5	6	8	2	1	4	11	7	3	9	10
C03374	1	9	8	3	4	5	11	7	10	6	2
C08353	1	2	8	5	4	6	11	10	9	7	3
C14279	1	11	5	2	3	8	9	10	6	4	7
C08688	10	9	11	2	5	7	4	1	3	6	8
C16260	3	1	2	5	4	6	11	9	7	8	10
C11847	5	10	1	9	6	11	3	4	8	7	2
C06870	5	10	6	4	2	8	1	3	7	9	11
C11773	5	10	1	6	8	7	4	3	11	2	9
C12159	9	5	8	2	11	6	3	10	7	4	1
C08104	10	9	11	5	6	3	1	7	2	8	4
C02366	6	2	5	8	10	1	11	9	7	3	4
C05601	5	8	1	2	9	11	6	4	3	7	10
C10083	10	3	4	7	6	11	2	8	1	5	9
C03104	4	2	1	7	5	6	11	3	9	10	8
C07256	5	11	10	8	2	9	1	6	3	7	4

C07575	5	10	1	6	8	2	3	11	9	7	4
C15997	4	11	10	8	6	2	5	9	3	7	1
C10366	10	9	11	5	6	4	8	3	1	7	2
C03654	1	2	9	3	4	10	8	5	6	7	11
C09593	10	11	8	1	5	2	9	3	7	6	4
C06948	5	11	10	1	8	3	6	2	9	7	4
C07327	1	8	11	2	3	6	10	5	9	4	7
C01541	10	11	8	5	1	3	9	2	6	7	4
C08153	3	9	11	1	8	10	2	5	4	6	7
C00478	1	10	11	6	7	4	9	3	2	8	5
C06418	5	6	2	1	8	10	4	9	11	3	7
C10696	10	1	4	2	5	11	9	8	6	7	3
C11251	3	9	11	5	8	10	6	2	1	7	4
C14584	9	5	11	1	6	7	3	2	8	10	4
C08270	5	6	2	8	4	1	10	11	7	9	3
C06438	3	5	8	11	7	6	10	9	4	1	2
C13677	2	11	3	7	4	6	1	5	9	10	8
C02785	8	2	11	7	5	4	3	9	6	10	1
C14650	3	9	1	4	8	10	11	7	6	5	2
C11611	1	10	11	5	6	7	4	9	3	8	2
C16435	5	6	1	2	8	10	4	9	11	3	7
C02577	10	11	5	3	8	2	4	1	9	6	7
C14492	6	2	4	3	8	5	11	10	7	9	1
C07360	10	5	2	8	6	4	9	1	3	11	7
C12043	5	8	10	9	11	3	2	1	6	4	7
C10893	10	11	1	5	3	6	4	2	8	9	7
C13728	5	10	6	1	2	8	4	11	3	7	9
C09655	10	3	9	5	1	11	8	4	7	6	2
C09631	9	11	3	10	5	8	7	2	6	1	4
C08935	1	11	10	4	9	7	2	8	3	5	6
C02843	1	5	2	3	11	8	9	6	4	10	7
C15501	1	5	2	11	8	6	4	7	9	3	10
C05649	8	2	5	11	6	9	10	1	7	4	3
C12590	11	1	4	7	9	6	10	3	8	2	5
C00200	1	2	8	11	5	6	10	3	4	9	7
C08230	8	5	2	1	6	11	10	4	9	3	7
C02132	11	5	1	2	9	6	8	3	7	4	10
C14478	3	1	5	2	4	11	8	9	7	6	10
C13914	3	1	2	9	4	5	8	11	7	6	10
C09102	10	1	8	7	3	9	11	4	5	2	6
C06485	1	2	8	6	10	9	4	3	11	5	7
C07367	11	9	6	10	2	5	4	7	8	3	1
C03766	6	2	1	8	10	5	11	9	3	4	7

C13967	10	1	9	5	6	2	8	11	4	3	7
C13733	5	2	4	6	8	1	3	10	11	9	7
C13993	10	9	11	4	5	6	8	1	3	2	7
C11659	10	9	6	5	3	8	1	11	2	7	4
C11119	2	5	6	10	1	8	3	4	7	11	9
C09095	5	3	6	7	2	10	11	8	1	9	4
C02864	5	10	9	6	8	4	7	3	11	1	2
C09680	9	8	2	11	5	3	10	7	1	4	6
C02796	2	6	8	5	10	7	4	11	3	1	9
C00902	5	6	10	8	1	2	11	9	4	3	7
C09287	10	9	3	11	1	6	4	5	8	7	2
C15771	5	1	2	6	11	8	10	4	9	7	3
C08032	9	10	6	11	7	8	4	1	3	2	5
C12755	10	5	9	2	6	7	3	1	8	11	4
C03443	5	2	6	11	1	4	10	9	8	3	7
C11020	11	2	8	10	6	1	5	3	4	9	7
C13670	11	6	10	8	5	1	2	4	3	9	7
C11322	3	1	11	2	5	10	9	7	6	8	4
C08405	10	6	3	1	7	8	5	11	2	4	9
C07040	10	5	11	3	1	6	2	4	8	9	7
C03710	2	5	6	8	4	11	3	1	10	7	9
C13756	10	9	5	11	4	3	6	2	7	8	1
C07010	5	10	11	3	4	7	6	1	2	8	9
C01771	1	11	5	3	2	8	6	9	4	10	7
C03804	2	7	8	9	6	1	4	3	10	11	5
C08127	3	2	7	9	8	6	11	10	5	1	4
C07128	10	11	5	2	1	4	6	8	3	7	9
C07604	10	9	4	7	11	3	2	6	8	1	5
C12174	10	7	2	1	9	8	3	4	11	6	5
C04359	1	7	4	3	2	5	11	6	9	8	10
C12178	5	10	7	1	11	2	8	3	9	4	6
C13857	3	1	8	11	2	5	10	6	4	9	7
C04576	1	10	2	8	11	5	6	4	7	3	9
C09588	10	11	4	2	8	3	6	5	7	9	1
C10757	9	8	10	7	6	5	11	3	1	4	2
C04776	5	2	3	6	8	1	4	9	11	10	7
C15634	8	11	5	3	6	4	9	10	1	2	7
C09267	9	10	5	1	11	2	8	6	7	4	3
C00580	2	5	8	11	1	6	9	3	4	10	7
C10203	10	2	4	6	1	3	5	8	7	11	9
C07617	3	10	5	11	9	8	6	2	1	7	4
C06528	6	2	10	5	8	9	4	11	7	1	3
C14426	11	9	10	2	1	3	6	7	8	4	5

C09274	10	5	1	6	3	7	9	11	2	4	8
C10540	10	2	5	6	9	8	4	1	7	3	11
C03661	1	10	8	11	6	9	2	3	4	7	5
C11280	10	9	3	8	5	1	11	6	4	2	7
C08783	9	10	6	5	11	3	4	7	2	8	1
C13646	10	5	11	8	7	3	9	1	4	2	6
C15713	11	2	10	5	4	6	7	1	3	9	8
C13058	5	1	2	6	8	4	10	11	3	9	7
C07379	10	8	11	5	2	4	3	1	7	9	6
C00883	1	10	7	3	4	9	8	11	2	6	5
C09981	10	3	9	11	2	1	5	8	6	4	7
C10765	10	5	11	8	9	1	2	7	4	3	6
C04856	2	4	9	10	7	1	8	3	5	11	6
C02812	1	6	4	2	5	7	9	11	3	10	8
C13817	10	4	2	5	6	1	3	8	9	11	7
C10498	10	2	9	1	11	4	5	6	8	3	7
C07228	3	2	5	11	9	10	6	4	8	1	7
C14689	1	2	8	5	6	4	9	3	11	10	7
C09243	5	10	9	6	7	1	11	4	3	8	2
C13280	8	10	5	3	2	1	4	11	9	6	7
C11261	6	5	8	1	11	2	3	10	4	9	7
C10106	10	5	8	6	9	7	4	2	3	11	1
C14216	11	5	8	9	3	10	4	7	2	6	1
C00466	1	2	5	8	9	6	11	3	4	7	10
C03901	5	10	2	8	1	7	11	3	6	4	9
C10219	10	11	5	2	7	8	6	3	9	4	1
C06800	11	8	2	6	10	9	1	4	5	3	7
C08936	10	8	6	9	11	3	7	5	2	1	4
C15602	11	8	2	5	6	10	1	9	4	7	3
C10501	10	1	9	5	11	8	6	4	3	2	7
C02571	5	1	3	6	11	8	9	2	10	7	4
C08497	9	3	11	1	5	10	6	2	4	7	8
C00943	4	10	2	7	9	8	5	1	3	6	11
C06818	5	10	6	2	11	8	4	1	9	3	7
C11584	1	4	3	5	8	9	10	2	11	7	6
C08122	9	10	8	5	11	1	4	6	3	7	2
C10820	9	4	10	2	6	3	1	7	8	5	11
C06381	2	6	4	5	11	1	8	10	7	3	9
C14303	3	4	11	10	5	2	1	8	9	7	6
C13511	8	11	10	1	2	5	4	3	9	7	6
C15996	2	6	3	1	8	4	11	7	5	9	10
C11708	10	5	11	4	8	6	3	7	1	2	9
C13729	10	4	3	2	5	6	9	1	11	8	7

C10084	10	5	1	11	9	8	2	6	3	4	7
C00698	1	11	5	2	8	6	3	4	10	9	7
C03427	9	3	8	2	6	7	5	11	4	1	10
C08367	3	1	6	10	9	8	5	7	11	4	2
C11210	5	1	6	2	4	10	3	7	11	9	8
C09623	10	3	1	7	9	11	6	4	8	2	5
C10337	11	7	1	8	2	5	3	4	10	9	6
C11216	2	3	11	9	8	10	1	7	5	6	4
C08503	1	11	6	5	9	3	8	2	4	7	10
C10734	3	5	6	1	8	7	9	11	2	4	10
C09217	10	9	5	1	8	4	2	11	3	6	7
C05918	8	11	5	9	2	10	7	3	6	4	1
C11507	11	2	8	1	3	5	4	6	10	9	7
C12954	4	10	3	9	2	8	5	6	11	1	7
C12842	3	10	8	5	9	6	7	4	2	11	1
C09922	10	11	2	1	5	8	3	4	9	6	7
C07542	5	10	11	3	6	1	7	4	8	2	9
C09268	10	11	5	1	8	9	6	3	4	7	2
C08112	10	9	6	5	11	3	1	2	4	8	7
C01352	3	5	9	2	8	4	1	11	6	10	7
C13167	10	5	3	4	8	6	2	9	11	7	1
C14415	11	2	5	1	6	10	8	4	7	9	3
C12677	3	11	5	1	10	7	8	2	9	6	4
C07434	11	5	10	8	6	1	4	3	9	2	7
C07045	10	5	9	11	2	7	1	8	3	4	6
C09210	10	3	7	2	11	8	6	5	4	9	1
C08535	11	9	10	5	4	2	7	6	3	8	1
C04103	4	9	1	3	7	6	2	10	11	8	5
C09264	1	10	5	2	6	9	8	3	11	7	4
C13687	11	1	6	2	9	7	10	8	5	3	4
C11029	11	6	5	1	3	2	8	9	4	7	10
C01871	2	5	9	8	3	10	7	1	6	4	11
C07635	3	2	1	8	11	4	5	9	6	7	10
C14249	3	9	8	6	2	7	1	5	11	10	4
C08486	2	10	8	5	11	3	6	4	9	1	7
C06925	3	8	10	5	9	1	7	11	4	6	2
C16241	8	5	2	1	6	11	10	9	3	4	7
C07140	3	10	9	4	5	2	11	8	1	6	7
C06854	8	9	10	1	2	11	5	7	3	6	4
C10356	10	6	1	2	9	7	11	3	8	5	4
C08616	3	2	10	8	6	9	11	1	7	5	4
C07600	10	5	8	9	11	2	3	6	4	1	7
C09018	11	9	10	1	5	6	2	3	8	7	4

C13910	3	11	1	6	5	9	8	7	10	2	4
C07433	5	10	1	7	4	9	11	3	8	6	2
C14524	6	2	7	4	9	5	1	8	3	11	10
C10136	1	5	8	3	11	6	9	2	10	4	7
C10760	10	9	5	8	2	4	3	11	7	1	6
C14532	11	10	2	3	5	1	8	7	9	4	6
C01839	2	11	5	8	1	4	6	9	10	3	7
C06880	10	9	6	5	11	2	1	4	8	3	7
C14384	11	7	9	4	6	10	3	2	1	5	8
C14458	10	11	3	7	9	8	6	1	5	2	4
C08145	11	3	10	2	4	6	9	1	5	8	7
C02595	5	10	11	8	9	2	3	1	7	4	6
C04639	4	2	5	8	1	6	9	3	10	7	11
C14259	3	11	5	1	8	2	10	9	4	7	6
C11081	11	2	1	6	3	5	7	10	8	9	4
C14490	1	8	7	2	5	3	6	11	9	10	4
C08054	9	10	11	5	8	4	1	3	7	6	2
C11438	9	6	8	10	5	4	11	2	3	7	1
C07245	5	10	8	1	6	11	2	9	3	7	4
C09778	10	9	4	11	5	6	2	7	8	3	1
C07827	10	5	11	4	3	6	1	8	7	2	9
C11059	11	1	4	10	2	8	3	9	7	6	5
C08441	4	9	8	2	10	1	3	5	6	11	7
C04075	2	5	6	8	11	4	10	9	1	3	7
C09131	10	5	8	3	6	9	11	4	2	1	7
C04695	1	4	9	10	8	3	2	6	7	11	5
C14685	11	10	5	1	8	6	2	9	3	7	4
C10591	2	5	4	6	10	11	8	7	1	3	9
C07588	11	5	8	3	2	1	9	6	4	10	7
C01183	2	8	6	11	1	5	10	9	3	4	7
C02655	1	2	10	7	8	6	9	5	4	11	3
C12250	11	4	8	6	7	3	10	1	5	9	2
C10549	9	3	7	10	6	5	11	4	8	1	2
C15572	11	5	10	9	8	1	6	3	2	4	7
C09178	11	8	5	6	1	2	7	9	3	4	10
C11476	1	4	2	8	5	9	10	6	7	11	3
C08968	3	7	2	10	6	11	1	9	8	5	4
C07815	5	10	3	8	4	11	2	1	9	6	7
C01785	11	10	5	1	8	2	3	6	9	4	7
C01066	11	8	1	2	3	5	6	10	7	9	4
C10996	9	11	3	5	6	1	7	4	2	10	8
C10645	11	1	3	6	5	7	8	4	9	2	10
C07120	3	11	9	6	2	5	1	8	4	7	10

C11035	8	5	3	2	6	4	11	9	7	10	1
C13665	5	10	11	1	8	2	3	6	7	9	4
C10316	11	10	9	3	5	7	1	6	8	4	2
C08597	8	9	1	4	3	7	5	11	10	2	6
C01783	11	3	1	6	5	2	8	9	4	10	7
C10437	10	5	4	1	6	11	8	2	9	3	7
C07710	1	3	5	9	11	4	10	2	8	6	7
C11727	11	5	1	2	4	9	10	8	6	3	7
C14531	11	10	5	6	2	8	9	4	1	7	3
C09281	10	8	2	1	6	4	5	3	11	9	7
C06687	10	9	11	4	6	2	8	7	1	3	5
C12486	1	11	2	10	5	3	6	4	8	9	7
C08075	3	9	11	5	6	2	8	7	1	4	10
C12304	11	1	9	5	3	6	10	2	7	8	4
C10196	10	2	5	8	7	4	11	3	6	1	9
C02957	1	4	6	9	5	2	3	11	7	8	10
C07221	10	9	6	5	1	7	8	3	11	2	4
C11779	10	11	8	2	3	6	9	4	5	1	7
C10383	10	11	2	5	4	9	7	8	1	6	3
C07884	5	10	11	6	8	1	3	2	4	9	7
C07148	1	10	5	2	4	9	11	3	8	6	7
C11065	11	8	6	2	5	1	10	9	7	4	3
C10159	5	9	10	8	7	2	11	1	3	6	4
C07440	3	10	5	11	1	8	6	9	2	4	7
C07198	3	11	10	9	8	2	5	4	1	6	7
C06701	2	6	9	4	8	5	10	1	7	3	11
C14325	11	4	7	5	6	2	3	9	1	8	10
C10933	11	6	4	2	3	5	1	10	8	7	9
C10747	10	5	2	1	8	7	3	4	9	11	6
C08349	1	2	5	4	6	3	9	8	7	10	11
C12819	5	6	10	2	8	1	4	3	11	9	7
C14467	2	11	8	10	5	6	3	1	4	7	9
C03878	1	5	2	8	10	6	3	11	9	4	7
C11595	3	8	6	2	1	9	5	7	4	11	10
C15368	3	5	8	4	10	9	11	1	7	2	6
C00038	1	5	8	2	4	6	9	3	10	11	7
C11391	9	11	5	10	8	4	1	3	6	2	7
C08459	1	4	2	6	3	5	11	10	9	7	8
C09972	11	10	5	7	2	3	9	1	8	6	4
C14208	3	1	11	6	5	8	9	4	7	10	2
C07658	10	9	8	11	5	2	4	7	3	1	6
C07518	5	2	1	10	11	6	4	8	9	3	7
C08874	1	10	7	3	4	5	6	2	9	8	11

C12886	3	8	10	7	6	11	4	5	1	9	2
C09361	2	10	8	11	7	6	4	3	5	1	9
C06323	10	8	11	2	5	9	6	3	4	1	7
C09900	9	11	8	2	3	1	5	6	10	4	7
C01067	1	2	10	4	8	5	3	7	9	6	11
C03734	6	2	9	10	8	4	1	5	11	3	7
C06532	8	10	5	2	9	1	3	6	4	7	11
C10368	11	8	1	5	2	10	6	3	4	9	7
C04218	2	9	4	10	7	11	6	1	5	3	8
C13206	10	9	6	11	5	1	8	7	3	2	4
C11381	10	5	9	8	6	3	4	2	11	7	1
C04871	5	3	11	9	1	6	2	4	10	7	8
C10576	6	5	10	11	3	9	4	1	7	2	8
C15474	5	10	11	9	8	6	2	1	3	7	4
C10556	2	8	10	6	11	5	3	9	4	1	7
C07295	9	5	10	11	3	6	2	1	4	7	8
C08420	10	4	5	11	6	3	9	2	8	1	7
C13526	1	11	3	10	6	2	5	9	8	7	4
C11686	11	4	6	8	7	9	3	2	5	1	10
C11218	5	6	10	8	1	2	3	7	9	4	11
C10967	5	10	2	7	4	9	8	3	1	11	6
C12798	10	5	2	4	11	7	6	1	3	9	8
C15858	9	8	10	5	2	3	6	11	1	4	7
C09627	2	8	9	6	3	10	5	11	1	4	7
C07829	11	5	4	10	9	8	2	6	3	1	7
C05079	10	9	5	4	2	11	6	8	3	7	1
C07556	5	10	2	11	6	3	8	7	1	9	4
C07107	5	10	11	9	8	4	1	3	2	6	7
C06008	5	8	6	2	1	10	11	4	9	3	7
C15756	1	7	9	5	2	6	10	11	8	3	4
C06439	3	2	6	7	1	11	4	5	10	9	8
C11361	3	1	11	10	5	2	8	6	4	9	7
C05126	5	10	11	1	7	4	6	8	2	3	9
C09382	10	2	9	6	8	11	4	3	1	7	5
C08567	10	5	8	11	1	4	2	3	9	7	6
C07943	5	11	10	1	8	2	4	3	9	7	6
C09305	8	2	5	6	9	3	11	1	10	4	7
C08072	3	11	5	9	6	2	10	1	8	7	4
C08839	3	5	10	9	11	8	6	4	7	1	2
C14557	11	7	6	4	5	8	1	9	2	3	10
C09625	9	11	3	2	5	10	8	1	7	6	4
C08811	9	3	2	10	7	5	8	4	11	1	6
C08365	3	1	2	11	6	7	9	8	5	4	10

C14688	11	1	8	4	9	10	3	2	5	7	6
C14546	3	5	10	1	11	9	7	8	2	6	4
C05334	10	5	8	11	3	2	9	7	4	1	6
C07539	11	1	2	3	5	10	8	6	9	4	7
C10812	11	10	5	9	1	8	4	2	6	3	7
C04205	3	5	8	6	9	1	10	4	2	11	7
C04036	6	2	3	8	7	4	11	10	5	1	9
C03634	10	3	1	5	4	2	6	8	11	7	9
C10870	10	2	4	11	5	8	3	7	9	6	1
C03188	5	3	10	1	9	2	11	6	8	4	7
C04587	6	2	8	9	10	4	3	1	11	7	5
C15549	10	1	5	9	8	11	3	6	2	4	7
C04715	9	1	4	3	7	2	5	10	11	8	6
C15270	8	4	11	9	5	10	6	3	1	2	7
C08388	9	2	11	3	5	4	8	1	7	6	10
C13051	8	2	1	10	4	9	6	7	11	3	5
C11527	11	5	8	2	9	1	10	6	7	4	3
C10066	9	3	8	4	7	1	5	11	2	6	10
C11165	3	9	11	5	1	6	2	8	4	10	7
C09951	10	11	9	4	5	6	3	2	1	7	8
C09834	3	5	4	11	6	7	10	9	8	2	1
C07551	5	10	2	11	1	4	6	3	7	8	9
C09729	9	5	11	3	7	8	10	4	1	6	2
C00747	8	2	11	1	5	6	10	3	9	4	7
C11240	11	4	10	6	9	2	7	3	8	5	1
C11906	8	6	3	2	5	10	4	11	9	1	7
C11687	11	1	5	2	4	10	8	6	9	7	3
C03505	2	8	3	1	6	10	7	11	9	4	5
C07117	3	5	10	11	4	6	9	2	1	7	8
C01386	11	1	5	6	3	10	8	2	7	9	4
C09633	9	10	2	8	7	1	3	6	4	5	11
C07062	3	10	5	11	1	9	2	4	8	7	6
C08517	10	2	8	1	7	5	11	3	9	4	6
C01546	2	5	8	11	10	1	9	3	4	6	7
C02896	5	6	2	8	10	1	9	11	4	3	7
C09198	1	3	2	9	10	11	5	6	7	8	4
C07016	3	5	9	6	11	2	10	7	4	1	8
C10986	11	3	9	1	10	7	6	5	4	8	2
C08837	9	1	5	11	8	3	4	6	7	2	10
C08331	6	5	2	4	10	1	11	8	3	7	9
C15688	9	1	4	10	11	5	6	3	2	7	8
C14275	11	5	2	10	4	8	1	9	6	3	7
C01408	11	5	8	1	10	2	6	7	9	3	4

C14411	11	8	6	4	5	2	1	3	10	9	7
C10188	10	11	6	4	3	1	8	7	5	2	9
C08236	1	2	5	10	7	8	3	11	6	9	4
C03725	8	7	6	2	1	3	9	4	10	5	11
C14290	10	5	4	2	9	1	11	6	3	8	7
C10636	10	11	7	3	6	4	9	2	5	8	1
C08800	9	7	2	1	6	5	8	3	11	10	4
C08648	10	3	5	1	11	2	7	9	6	8	4
C06972	10	5	11	2	8	3	6	4	1	9	7
C01995	1	11	2	6	5	3	9	8	10	4	7
C07513	5	10	3	4	2	1	9	11	7	8	6
C12228	10	3	6	4	7	8	9	5	11	2	1
C11074	5	11	4	2	7	8	9	3	10	1	6
C10229	11	1	4	9	7	10	3	8	5	2	6
C06909	4	6	9	10	5	8	3	11	7	1	2
C08458	10	9	2	7	4	11	8	3	1	6	5
C01728	1	2	10	8	6	5	11	4	9	7	3
C11597	3	6	1	9	5	2	8	11	10	7	4
C10717	1	11	4	6	3	8	5	2	9	7	10
C10628	2	10	4	1	9	3	6	8	7	11	5
C07661	9	10	11	5	8	4	1	6	3	2	7
C07519	11	2	10	5	6	1	4	8	7	3	9
C08264	5	6	2	4	8	10	11	7	1	3	9
C02306	11	2	4	5	6	1	7	8	10	9	3
C01523	3	1	6	5	7	8	11	4	2	9	10
C13806	6	5	10	8	1	2	7	4	3	9	11
C14261	3	11	8	5	9	1	7	6	10	2	4
C10524	10	1	3	2	5	9	6	7	11	4	8
C07042	10	11	8	5	1	4	7	3	6	2	9
C09384	10	1	6	11	5	4	2	3	8	9	7
C15740	5	10	8	9	1	11	3	7	6	2	4
C07127	3	5	9	2	7	1	8	11	10	4	6
C06347	10	5	9	11	3	8	6	2	4	1	7
C02899	8	5	10	2	1	6	4	11	7	3	9
C10451	9	11	10	2	5	4	6	7	8	3	1
C08983	1	10	3	8	5	2	7	4	6	9	11
C07971	11	10	9	1	2	5	3	7	4	8	6
C00849	11	10	1	5	9	2	3	8	6	7	4
C02956	1	3	4	8	5	7	11	10	6	2	9
C13860	3	1	7	10	8	4	11	9	6	2	5
C09916	11	2	9	7	8	6	10	3	4	1	5
C09808	11	10	8	5	7	2	6	9	3	1	4
C08123	9	10	1	7	5	6	3	11	2	4	8

C09049	11	10	5	1	8	4	7	3	2	6	9
C09206	10	6	7	4	11	2	3	1	5	8	9
C07236	4	9	10	6	11	2	3	5	8	7	1
C14345	11	10	4	9	1	7	3	2	8	5	6
C14738	3	8	6	5	7	11	4	9	2	1	10
C03039	9	3	4	6	11	1	2	5	7	10	8
C01420	5	6	2	8	10	1	9	4	11	7	3
C10032	10	11	5	4	1	8	7	3	9	6	2
C14460	11	10	5	7	8	3	4	6	9	1	2
C09615	10	5	3	9	1	4	2	11	8	7	6
C01875	9	11	5	1	4	6	2	3	10	8	7
C12049	9	1	11	4	2	10	5	7	3	8	6
C07400	11	2	10	6	8	5	3	1	4	7	9
C01770	1	5	2	11	10	3	9	8	4	6	7
C07844	10	1	11	2	3	6	5	4	7	8	9
C08394	9	8	5	10	1	11	3	7	2	6	4
C02821	1	2	11	5	3	8	6	9	4	10	7
C09224	10	11	5	9	8	6	3	4	7	1	2
C13786	10	3	1	2	6	4	8	7	11	9	5
C06955	10	5	2	3	11	9	8	6	1	4	7
C00551	1	2	9	4	10	3	11	7	8	6	5
C13110	10	11	7	5	6	3	1	2	4	9	8
C14189	10	11	3	8	2	4	1	5	6	9	7
C03743	11	2	6	8	4	5	1	9	3	7	10
C08511	9	6	4	10	11	3	2	5	1	7	8
C16436	5	6	2	1	8	10	4	9	11	3	7
C03448	8	2	11	9	5	10	4	1	3	6	7
C15432	3	10	9	6	7	5	11	4	8	2	1
C02726	4	2	9	5	10	1	3	7	8	6	11
C09342	10	11	1	4	9	3	8	7	6	5	2
C09226	10	11	5	3	7	2	1	6	4	8	9
C00728	4	9	6	7	11	5	1	10	2	3	8
C00765	1	11	5	10	8	2	9	6	7	3	4
C05231	5	2	1	3	11	9	6	8	4	7	10
C02717	10	5	8	3	11	4	2	9	6	7	1
C14281	8	10	11	9	7	1	5	3	6	4	2
C13342	11	5	1	9	6	7	2	3	8	4	10
C11323	11	1	2	3	4	5	10	9	7	6	8
C10935	9	11	5	4	2	10	1	6	3	8	7
C10395	5	11	1	8	9	6	10	3	2	7	4
C11841	2	5	9	8	3	7	6	4	11	10	1
C11277	4	11	6	9	10	1	5	3	8	7	2
C10467	10	4	6	9	11	8	3	2	7	5	1

C15726	5	9	1	2	6	3	4	8	7	10	11
C08299	5	10	11	4	1	2	3	8	6	9	7
C14258	11	3	1	9	10	5	6	2	8	7	4
C10708	11	4	1	6	7	8	5	10	3	2	9
C08753	5	4	1	11	2	7	6	8	3	9	10
C14464	11	5	3	2	8	4	9	10	1	6	7
C10555	6	5	8	10	1	2	4	9	11	3	7
C12054	10	9	1	11	2	5	3	7	4	6	8
C07224	10	5	4	3	2	8	11	1	6	9	7
C06342	2	8	5	10	11	9	7	3	4	1	6
C09075	3	4	9	8	5	11	7	1	10	6	2
C03514	10	1	9	6	8	3	2	5	11	4	7
C14133	11	3	10	1	9	6	8	2	5	7	4
C07279	6	3	1	9	2	10	11	5	4	7	8
C08922	10	7	4	1	9	5	3	6	11	8	2
C15441	3	1	8	2	7	9	5	4	6	10	11
C07552	11	10	3	9	1	5	2	7	8	4	6
C07049	5	10	11	3	2	9	4	8	6	1	7
C09773	1	9	10	3	2	5	4	6	11	8	7
C07416	5	11	8	4	6	7	2	10	3	1	9
C02314	3	5	10	4	2	1	6	9	8	11	7
C15522	5	8	10	2	9	6	4	7	3	11	1
C07813	3	10	5	2	1	8	11	4	6	9	7
C14205	11	3	10	6	5	8	4	2	7	1	9
C11225	10	1	3	9	2	11	8	5	4	6	7
C04114	5	2	8	1	3	11	9	6	10	4	7
C14270	11	5	8	2	6	1	4	7	9	10	3
C09746	10	9	6	8	3	1	11	4	2	5	7
C15228	3	2	8	9	7	11	4	1	10	6	5
C09637	9	10	6	2	8	1	11	3	5	4	7
C16485	11	8	5	10	9	6	7	4	2	1	3
C04264	6	8	5	1	2	10	9	3	11	7	4
C06914	9	10	8	6	5	1	3	11	2	4	7
C07571	5	10	11	3	9	6	2	7	1	4	8
C12166	10	3	11	6	8	5	2	1	9	4	7
C03584	6	9	2	4	8	5	1	7	10	11	3
C15751	5	9	4	8	7	2	10	3	11	1	6
C00629	1	2	8	6	9	5	4	10	11	7	3
C13937	3	7	9	4	8	11	1	6	5	10	2
C10983	11	9	10	2	3	1	7	5	8	4	6
C07834	11	1	3	5	10	6	7	2	9	8	4
C03754	5	2	9	10	4	1	8	6	11	3	7
C08617	1	10	3	5	9	2	4	6	8	11	7

C10149	5	6	1	10	8	4	2	11	9	3	7
C08268	5	6	10	2	8	4	1	7	9	3	11
C13844	9	2	8	10	5	7	3	11	1	4	6
C08103	10	9	11	5	6	3	1	8	7	2	4
C12662	9	6	8	10	11	1	2	5	7	3	4
C12753	10	3	11	9	2	5	1	4	6	8	7
C00210	8	2	5	6	3	11	9	1	4	7	10
C06359	9	10	5	11	6	2	8	1	7	3	4
C07750	10	1	11	9	7	2	6	4	8	5	3
C13837	5	4	2	6	8	7	10	3	9	11	1
C08571	9	11	5	10	1	2	6	3	7	4	8
C03987	5	7	4	2	3	6	10	9	11	8	1
C01868	2	8	9	5	11	6	7	3	10	4	1
C01158	1	10	4	9	11	3	7	6	5	8	2
C07240	3	1	9	8	11	10	5	4	2	7	6
C14199	11	5	4	6	8	7	9	3	10	1	2
C16217	3	5	1	9	6	7	11	4	10	8	2
C02704	2	10	1	5	4	8	6	11	3	9	7
C07356	10	11	5	8	1	3	9	2	6	4	7
C13812	3	7	8	2	4	10	11	5	6	1	9
C11795	11	10	5	1	3	7	2	8	9	4	6
C00892	2	6	8	5	4	1	9	10	11	3	7
C14420	11	10	9	2	1	3	8	5	6	4	7
C07632	3	2	8	6	5	1	4	10	7	11	9
C07502	11	5	1	6	10	8	2	4	9	3	7
C04360	1	10	11	7	8	2	5	3	6	9	4
C11760	10	9	7	4	8	1	3	11	5	2	6
C07569	5	4	1	11	6	9	2	10	3	8	7
C02509	2	8	7	11	5	1	3	10	6	9	4
C09955	11	5	10	3	2	8	4	9	1	7	6
C11618	10	5	9	11	8	4	6	3	2	7	1
C11037	11	6	1	5	8	3	9	4	10	2	7
C07339	3	10	5	9	1	8	4	11	6	2	7
C00908	10	9	3	6	1	11	2	5	8	4	7
C01765	9	1	11	8	2	10	7	6	5	4	3
C06879	10	5	9	6	1	11	3	8	4	2	7
C07497	11	10	5	3	1	9	2	4	6	7	8
C09026	10	5	1	11	6	7	3	4	8	9	2
C08946	10	3	9	4	6	1	5	7	11	2	8
C06746	2	8	5	9	7	6	1	10	11	3	4
C10569	10	11	6	4	2	7	3	9	8	1	5
C13758	3	10	5	1	4	2	8	9	6	11	7
C11754	10	9	5	6	8	1	2	11	7	4	3

C02216	4	3	1	8	10	5	9	6	11	2	7
C12962	6	4	5	3	2	9	1	11	10	7	8
C08282	3	7	9	8	11	10	1	2	6	5	4
C07846	5	10	2	11	1	4	9	6	7	8	3
C03338	2	8	11	1	7	9	6	5	10	4	3
C11221	6	5	3	1	8	4	10	7	11	9	2
C14422	11	5	6	3	2	10	1	9	4	8	7
C05860	4	2	1	6	3	7	8	10	5	9	11
C10656	3	10	11	2	1	5	8	9	6	7	4
C02277	3	11	1	8	9	10	2	5	7	6	4
C11062	1	11	5	3	6	8	2	9	7	10	4
C05464	3	6	8	11	10	7	5	4	1	9	2
C16038	11	10	8	5	4	9	2	3	1	7	6
C10614	10	5	9	11	1	6	4	2	7	3	8
C11590	5	9	10	8	1	2	6	3	7	4	11
C14617	2	5	4	6	1	11	3	9	10	7	8
C10064	3	10	8	7	11	5	2	4	6	9	1
C06834	9	3	2	5	1	8	4	11	10	6	7
C07429	8	4	11	6	7	10	1	3	5	9	2
C09000	3	10	4	1	6	5	9	11	2	7	8
C14330	11	5	2	4	1	8	7	3	6	9	10
C00928	1	5	2	8	6	11	3	9	10	4	7
C03591	11	1	6	5	10	9	4	8	3	2	7
C10678	9	5	11	10	3	6	8	4	1	2	7
C08646	10	7	6	8	4	3	9	1	11	5	2
C10523	10	4	1	7	11	6	8	3	5	9	2
C08652	10	5	9	8	11	3	1	6	7	4	2
C07553	5	10	2	11	6	1	4	3	8	7	9
C06469	1	3	10	7	5	6	2	4	9	8	11
C10391	10	11	8	5	2	6	7	3	1	4	9
C12295	9	10	2	7	1	11	4	3	8	5	6
C10456	10	5	9	2	6	1	7	8	4	3	11
C04403	1	7	4	9	2	8	10	3	6	11	5
C14077	8	11	2	6	10	1	4	5	3	7	9
C11745	5	8	2	3	6	1	9	7	10	11	4
C07677	10	5	4	1	3	2	9	8	11	6	7
C16488	1	5	2	4	7	6	10	3	8	11	9
C07607	3	10	1	5	2	8	7	9	4	11	6
C08594	9	8	10	6	5	11	3	1	2	4	7
C07669	10	11	4	5	3	9	1	2	8	6	7
C09492	9	10	1	3	4	8	11	2	6	7	5
C10182	10	1	5	3	11	2	9	7	6	8	4
C14313	10	7	8	1	9	3	11	4	2	5	6

C07582	1	8	5	2	4	10	11	9	7	3	6
C03313	8	5	6	10	11	1	2	9	4	3	7
C14132	11	3	10	5	7	2	4	8	1	6	9
C09760	3	4	2	1	11	8	9	10	6	5	7
C09943	11	1	4	6	8	9	10	3	2	7	5
C10234	10	8	5	1	2	3	6	4	7	9	11
C10088	10	8	1	4	7	2	5	3	9	6	11
C07326	1	2	6	8	10	7	11	5	3	9	4
C10957	5	10	2	6	8	1	9	11	7	3	4
C09693	9	3	2	5	1	7	8	11	6	4	10
C03783	1	6	5	2	3	10	8	9	7	4	11
C06872	3	10	7	4	1	5	11	6	8	2	9
C06736	2	8	3	1	4	6	11	10	5	7	9
C14586	3	2	1	11	5	4	7	6	8	10	9
C02681	3	8	7	6	9	11	4	2	5	10	1
C06390	3	8	4	5	2	7	10	11	6	1	9
C12050	9	5	11	6	3	7	1	10	4	2	8
C03348	1	9	7	10	6	2	4	8	11	3	5
C02828	6	9	1	7	2	11	3	5	8	4	10
C03139	5	6	2	8	4	11	3	10	9	7	1
C11797	11	10	5	3	9	4	1	2	8	6	7
C05203	10	5	9	8	2	3	11	4	1	7	6
C09423	11	10	8	9	6	4	3	5	1	2	7
C07668	5	10	4	11	1	9	3	2	6	8	7
C04000	2	6	8	9	1	7	5	3	10	4	11
C10430	11	9	10	5	3	4	7	1	2	6	8
C12023	11	10	9	2	7	8	4	1	6	5	3
C15623	8	5	4	11	9	1	2	3	10	7	6
C07570	5	10	11	3	9	1	4	7	8	6	2
C05198	5	8	1	2	4	6	9	10	11	3	7
C09220	11	1	3	9	7	2	5	8	4	10	6
C07470	5	11	10	9	8	1	4	6	7	3	2
C06940	8	5	11	9	1	6	2	10	4	3	7
C07252	5	10	6	11	1	2	8	3	7	9	4
C13050	8	4	2	1	10	9	6	7	5	11	3
C00927	6	10	5	7	2	8	1	11	3	4	9
C11307	1	11	3	5	2	6	4	10	9	7	8
C00809	9	8	2	11	5	6	1	3	10	4	7
C01564	11	10	6	2	5	4	8	9	1	7	3
C09841	9	10	1	4	8	3	11	5	7	6	2
C08637	3	10	9	1	11	5	6	2	8	4	7
C00503	1	2	3	5	8	6	9	10	11	7	4
C10091	10	6	4	2	1	8	11	3	9	5	7

C12065	9	10	5	6	2	3	1	8	4	7	11
C12163	10	2	11	6	8	3	7	1	4	5	9
C06871	5	3	10	1	7	11	6	9	4	2	8
C09639	9	3	2	1	11	10	6	5	8	7	4
C11256	6	9	2	4	8	7	3	5	11	10	1
C07411	1	2	6	5	3	10	8	9	11	4	7
C04452	1	3	2	10	6	8	11	9	7	5	4
C10142	10	11	1	4	8	9	7	6	3	5	2
C11223	8	11	9	2	6	7	1	4	3	5	10
C07819	10	5	3	2	11	8	4	7	9	6	1
C11164	1	2	6	8	5	10	11	9	4	3	7
C13038	3	1	2	11	5	6	8	9	10	4	7
C09557	6	5	8	2	10	7	9	1	4	11	3
C09805	10	5	11	8	1	9	7	3	2	6	4
C07550	10	2	5	4	6	3	11	1	7	8	9
C02590	7	4	2	8	9	1	3	10	5	6	11
C07310	3	1	2	8	5	11	6	10	4	9	7
C02304	2	5	6	8	11	1	4	10	3	9	7
C11497	8	2	11	3	5	1	10	9	7	6	4
C08130	1	3	2	5	10	4	11	6	9	7	8
C06152	1	8	3	2	5	4	10	9	6	11	7
C13016	9	10	11	6	2	5	1	4	3	8	7
C01225	1	3	2	10	8	7	5	4	9	6	11
C09854	3	5	7	6	9	11	2	4	8	1	10
C14178	4	9	2	5	10	8	1	3	6	7	11
C09802	9	10	3	5	1	2	6	11	7	8	4
C08718	10	5	11	8	9	2	3	6	4	7	1
C10742	5	11	10	6	3	8	9	7	4	1	2
C10885	10	3	6	8	4	1	9	7	11	5	2
C12024	9	11	5	6	2	8	7	3	4	10	1
C07428	5	1	11	10	3	9	2	8	6	7	4
C11771	5	2	9	6	7	3	10	1	8	11	4
C14427	11	2	1	10	9	3	7	4	8	5	6
C09145	9	1	10	2	8	5	7	6	3	4	11
C07142	10	2	11	5	4	1	6	8	7	3	9
C12656	3	10	4	6	1	11	5	8	9	2	7
C10394	1	11	4	7	9	2	5	3	10	6	8
C09214	10	5	6	8	11	9	2	1	3	7	4
C14446	2	7	11	8	6	9	3	10	4	5	1
C07711	3	9	5	10	1	11	6	4	7	2	8
C07126	8	2	11	1	5	9	7	3	10	4	6
C14696	9	2	1	3	11	5	8	10	6	7	4
C02097	1	9	2	10	4	8	6	7	5	3	11

C14334	11	10	2	9	5	4	6	7	3	1	8
C15992	3	9	11	8	5	4	10	6	2	1	7
C10548	3	10	2	5	11	4	7	8	1	6	9
C04597	8	2	7	11	1	6	10	3	4	5	9
C08551	10	9	1	4	8	5	3	2	11	6	7
C10938	11	5	1	2	10	3	6	7	8	9	4
C10262	9	7	6	11	4	3	10	5	1	2	8
C02709	11	2	9	6	1	3	5	4	7	10	8
C02078	10	9	6	5	3	2	1	8	11	4	7
C14255	3	1	2	10	4	11	6	5	9	8	7
C10277	10	2	5	6	9	4	3	8	7	1	11
C11236	10	9	5	11	6	2	7	3	8	1	4
C10404	1	11	3	10	6	2	8	5	7	9	4
C14171	10	7	9	2	6	5	8	3	1	11	4
C01403	11	9	5	8	3	2	10	1	7	4	6
C00288	1	5	2	8	6	11	3	4	9	10	7
C03525	1	11	8	10	4	6	3	5	9	2	7
C14263	8	2	10	11	7	9	1	3	6	4	5
C11706	3	9	5	11	4	7	8	1	6	10	2
C09218	10	9	11	5	8	3	6	2	4	7	1
C08593	9	8	11	1	5	2	7	3	10	6	4
C14760	8	9	10	3	5	11	6	2	7	4	1
C10472	11	9	3	10	8	5	6	2	7	1	4
C09282	9	10	2	8	11	7	3	5	4	6	1
C03225	5	1	2	6	3	7	8	9	11	10	4
C07458	8	11	5	2	9	10	4	6	1	7	3
C14370	11	5	4	6	1	8	3	2	9	7	10
C03679	5	2	11	6	4	8	10	9	7	3	1
C07461	1	3	5	2	11	4	9	10	7	6	8
C02945	3	8	9	10	2	6	5	7	1	11	4
C11321	1	2	3	8	6	4	9	5	10	11	7
C08055	11	4	5	8	2	10	3	9	1	7	6
C06350	10	5	11	1	3	6	9	7	8	4	2
C04131	5	2	6	4	8	10	11	1	3	9	7
C08798	10	2	11	5	6	9	7	3	8	1	4
C02592	3	6	4	8	5	2	9	7	10	1	11
C04655	3	8	2	1	11	5	6	7	4	9	10
C11703	5	10	3	4	2	1	9	11	6	8	7
C10315	10	2	11	8	6	1	3	5	7	9	4
C09752	10	1	4	5	11	9	3	2	7	8	6
C14324	11	10	1	9	5	2	3	6	7	4	8
C04433	9	7	2	1	3	5	11	4	10	6	8
C09529	6	5	1	8	10	11	3	2	4	9	7

C10751	10	5	6	9	8	4	11	3	2	1	7
C01782	11	5	8	10	6	9	3	2	7	4	1
C11796	10	11	5	2	1	9	3	4	7	6	8
C11787	10	11	5	7	1	6	3	8	2	9	4
C10525	10	6	9	5	4	2	7	3	1	8	11
C10504	10	2	5	9	11	1	8	6	3	4	7
C10598	10	5	9	6	2	8	3	11	4	7	1
C10017	10	3	1	7	4	2	11	8	5	6	9
C07602	3	5	10	6	8	2	9	1	4	11	7
C06977	1	8	5	3	11	2	4	7	9	10	6
C06230	2	9	4	8	7	10	11	5	3	6	1
C08381	3	9	8	5	10	7	2	6	11	1	4
C01692	11	5	10	8	3	6	1	2	9	4	7
C08622	3	2	7	1	5	8	6	10	11	4	9
C06526	10	1	2	9	8	5	11	6	4	3	7
C14309	11	3	1	8	6	7	2	9	4	5	10
C10997	1	5	10	6	8	11	2	4	9	3	7
C12047	10	9	4	2	8	1	3	7	6	5	11
C09954	10	11	1	2	5	4	8	3	6	9	7
C02033	10	7	6	11	5	3	8	2	1	9	4
C03690	11	3	2	8	5	7	9	1	6	4	10
C08632	10	1	5	7	3	9	8	2	4	6	11
C07078	10	9	5	2	11	8	1	7	6	3	4
C13524	10	11	5	8	9	2	4	3	1	6	7
C00799	10	3	1	11	5	2	8	9	4	6	7
C09460	10	5	9	4	6	7	11	3	8	2	1
C14226	11	3	5	10	7	6	8	4	2	1	9
C03837	2	1	8	9	11	6	10	3	7	5	4
C10638	3	5	7	1	4	11	6	10	8	2	9
C13884	1	2	5	4	11	10	3	8	6	9	7
C02079	3	1	10	2	4	5	11	9	6	8	7
C07671	3	10	5	9	11	7	1	2	6	8	4
C10086	2	8	6	10	1	3	9	7	11	4	5
C10958	3	4	2	5	11	7	9	1	10	6	8
C15482	9	7	8	10	5	2	11	4	6	3	1
C11269	5	10	9	1	4	3	6	11	2	7	8
C14710	1	9	2	11	5	3	10	6	8	7	4
C08030	9	10	8	6	5	1	11	2	4	3	7
C11619	10	9	6	5	3	1	8	4	7	11	2
C08602	9	8	2	3	7	1	11	4	5	6	10
C03017	5	1	9	3	6	10	8	2	11	4	7
C09001	10	11	5	1	8	3	4	9	6	2	7
C14202	11	8	5	6	10	7	3	4	2	9	1

C08240	1	2	10	8	6	5	11	4	9	7	3
C08169	3	9	10	5	11	1	2	6	7	8	4
C01445	4	11	9	5	6	2	10	7	1	8	3
C10720	10	11	6	5	1	9	3	7	2	8	4
C05113	8	11	1	5	2	6	9	10	3	4	7
C10716	10	5	2	11	8	1	6	9	3	4	7
C10077	10	1	8	11	3	2	9	6	4	5	7
C10841	10	8	11	2	4	3	6	7	5	9	1
C08748	11	9	10	5	2	8	7	1	3	6	4
C09022	10	5	2	8	11	9	1	3	7	6	4
C01618	2	3	8	10	7	1	4	11	5	9	6
C11695	10	3	6	8	2	5	9	1	11	7	4
C10201	3	8	4	7	5	10	2	11	9	6	1
C13777	10	2	1	5	4	8	9	3	7	11	6
C03724	8	2	5	9	10	1	3	11	6	7	4
C07874	11	1	9	6	3	2	5	8	10	4	7
C09461	10	9	7	5	3	2	6	1	4	11	8
C14213	6	10	9	2	4	3	5	7	1	8	11
C06935	5	10	3	9	11	8	2	1	4	6	7
C14545	3	1	8	6	10	7	11	5	9	2	4
C08416	5	10	6	7	2	8	1	3	11	9	4
C12007	1	5	8	10	7	9	3	2	6	11	4
C12289	9	11	5	6	8	7	4	3	10	2	1
C13179	10	9	11	4	5	8	6	1	2	3	7
C15095	10	1	11	2	3	9	5	7	8	6	4
C09747	2	3	8	9	10	5	1	11	7	4	6
C08261	8	11	5	3	4	1	9	10	6	2	7
C06465	1	2	10	5	6	8	3	4	11	9	7
C06664	10	6	7	2	5	4	3	9	8	1	11
C14210	3	5	9	6	10	8	7	11	4	1	2
C13311	6	10	4	2	1	11	9	3	7	8	5
C06391	3	8	9	4	6	10	1	11	5	2	7
C04641	1	4	2	9	3	7	8	5	11	6	10
C09908	9	11	5	10	8	1	2	4	3	6	7
C09084	10	1	9	2	11	5	8	4	6	3	7
C04223	2	11	8	5	3	10	9	1	7	6	4
C05146	4	11	5	8	6	3	10	2	1	7	9
C15635	11	1	9	8	5	3	4	2	6	7	10
C11075	11	10	2	9	5	7	6	3	4	1	8
C08109	9	6	10	5	4	2	11	1	8	3	7
C03189	1	2	3	8	5	6	9	10	4	11	7
C08944	6	10	2	5	8	4	3	1	11	7	9
C04104	5	2	6	8	1	10	11	4	9	7	3

C12285	1	10	5	8	11	2	7	3	9	4	6
C13680	9	10	8	5	6	2	7	11	4	3	1
C09676	9	5	10	6	2	11	4	1	7	3	8
C14387	11	9	10	2	7	1	8	3	5	4	6
C10191	10	5	8	7	4	3	6	2	11	9	1
C05888	1	7	4	10	9	2	8	5	11	3	6
C14394	11	2	8	5	6	4	10	3	9	7	1
C13673	2	7	1	6	9	3	10	5	11	4	8
C08192	4	5	11	10	8	1	3	2	6	9	7
C06686	10	9	8	3	11	1	6	5	4	2	7
C02427	5	2	6	4	10	8	7	11	1	3	9
C00895	4	9	6	7	11	5	1	10	2	3	8
C09181	9	10	1	11	4	3	5	2	7	6	8
C09308	10	1	2	8	6	5	7	3	9	4	11
C13024	9	6	2	8	3	7	1	4	10	11	5
C14694	5	11	6	2	10	3	8	9	7	4	1
C02293	2	8	1	11	4	6	5	10	9	3	7
C14733	1	2	8	5	11	4	6	9	3	10	7
C07323	3	5	9	10	11	7	2	1	8	4	6
C13675	6	8	5	2	1	11	10	3	7	9	4
C14289	11	6	2	5	4	1	10	3	8	9	7
C00939	4	8	9	10	1	5	11	6	2	3	7
C15619	8	11	5	9	10	4	6	3	2	7	1
C09101	3	1	10	7	11	4	5	6	2	8	9
C07499	11	10	7	9	6	8	3	2	4	1	5
C13547	3	4	8	1	2	9	11	5	7	6	10
C02283	3	2	10	5	8	1	6	4	9	11	7
C09028	10	1	5	4	8	6	9	11	2	7	3
C12277	2	1	6	8	4	11	5	9	10	7	3
C07792	3	5	10	4	9	11	2	8	7	1	6
C11118	1	8	11	9	3	10	2	4	5	6	7
C12072	10	11	3	5	6	9	2	1	4	8	7
C10079	10	3	8	5	11	2	9	7	1	6	4
C06045	5	10	11	8	6	1	2	3	9	4	7
C08307	5	8	10	11	7	1	3	6	9	2	4
C06850	10	5	4	3	2	8	9	11	1	6	7
C07453	5	10	3	11	1	4	9	2	8	7	6
C03795	4	9	2	10	11	5	6	7	3	1	8
C14709	11	8	2	10	1	4	3	7	5	6	9
C14218	11	4	6	3	5	7	8	10	1	9	2
C06742	2	8	5	6	9	1	7	11	10	3	4
C12444	9	8	4	5	11	3	2	10	6	7	1
C09720	9	10	8	4	5	1	3	2	11	6	7

C09748	10	6	5	9	2	8	7	4	1	3	11
C04227	5	10	6	3	1	8	2	7	4	9	11
C07798	10	5	3	2	11	4	9	7	8	1	6
C10099	10	2	4	7	9	5	8	3	11	6	1
C12567	1	2	11	8	9	7	4	5	6	10	3
C07524	10	11	5	3	1	2	6	9	4	8	7
C15615	10	4	6	11	5	8	3	9	1	2	7
C08700	9	10	8	11	1	2	3	7	6	5	4
C02911	1	5	2	8	6	11	3	9	10	4	7
C12101	11	5	6	9	8	7	2	1	10	3	4
C04447	2	4	8	6	5	10	3	1	7	11	9
C04521	1	11	2	8	5	6	7	3	4	10	9
C01837	2	8	1	11	6	3	5	10	4	9	7
C03958	5	11	10	1	2	3	6	4	8	9	7
C10821	9	11	1	4	10	3	5	8	7	2	6
C11239	10	3	5	8	2	6	11	1	9	4	7
C04711	2	6	7	1	8	9	4	3	10	11	5
C03982	5	10	9	1	8	2	11	4	6	3	7
C09642	5	10	1	11	6	3	9	8	2	4	7
C03571	5	6	10	8	2	7	11	1	3	4	9
C05418	3	9	11	8	5	7	6	10	2	1	4
C11342	10	9	1	11	4	5	3	2	8	6	7
C09602	3	1	10	2	5	7	11	9	8	6	4
C08340	5	10	11	8	3	1	2	7	4	9	6
C12292	9	1	5	3	11	6	7	2	10	8	4
C04552	5	9	7	10	2	8	6	3	11	4	1
C07574	11	5	10	9	4	2	6	8	3	1	7
C11177	3	4	2	1	5	11	9	10	8	7	6
C12227	10	5	3	2	1	6	4	8	11	9	7
C00832	10	9	5	6	11	2	1	3	4	8	7
C13753	4	3	2	8	7	11	10	9	5	6	1
C14222	11	3	1	10	5	9	4	7	8	6	2
C12164	10	3	4	5	1	2	7	11	9	6	8
C15213	3	11	7	2	6	8	10	1	5	9	4
C15478	1	10	7	3	8	5	4	9	11	2	6
C12115	5	11	8	1	2	3	6	9	10	4	7
C14566	11	6	4	5	2	1	9	7	10	3	8
C04445	5	2	10	1	11	8	7	9	4	3	6
C14690	2	5	3	6	1	11	4	8	10	9	7
C04537	1	4	7	10	9	6	11	3	8	5	2
C13693	11	5	1	4	3	6	10	9	8	2	7
C14520	11	2	1	3	10	6	8	9	5	4	7
C16487	1	2	11	6	8	5	3	4	10	7	9

C10514	10	5	9	3	2	4	6	1	7	11	8
C10364	10	9	6	5	2	11	7	1	3	8	4
C10872	10	11	5	1	3	8	2	6	7	9	4
C10679	11	3	6	8	9	10	1	2	4	7	5
C15720	10	5	1	6	9	7	8	4	3	11	2
C10748	10	3	1	11	2	7	5	9	6	4	8
C00525	5	6	2	8	10	1	9	11	4	3	7
C08642	10	5	2	7	3	8	6	9	4	1	11
C11301	3	4	2	7	8	11	5	6	9	10	1
C07640	11	10	9	8	4	5	6	7	2	1	3
C14667	3	10	4	2	9	6	11	5	1	8	7
C12051	4	8	1	5	3	9	2	7	6	10	11
C02703	5	2	6	8	4	1	3	11	10	9	7
C11179	11	10	5	6	2	4	8	1	3	9	7
C15676	1	9	2	6	5	7	4	3	8	11	10
C14605	8	11	3	1	6	2	4	9	10	7	5
C09768	1	9	10	2	8	3	6	5	11	4	7
C05204	10	9	11	7	3	1	5	8	6	2	4
C07423	10	1	5	6	11	3	4	2	8	9	7
C06823	10	5	9	3	1	4	2	8	11	6	7
C04586	2	6	8	10	1	5	7	3	9	11	4
C06895	10	9	6	5	7	2	1	11	3	4	8
C04755	1	8	2	7	5	10	4	9	3	11	6
C02895	1	2	4	8	5	9	3	7	6	10	11
C15548	1	10	3	6	11	2	9	4	5	8	7
C07598	1	5	8	2	6	11	3	9	7	4	10
C07573	8	2	1	9	3	11	6	10	7	4	5
C14503	3	9	11	5	7	2	1	4	10	8	6
C05111	3	6	2	8	9	10	4	7	11	1	5
C14593	3	11	5	4	8	6	7	1	9	2	10
C09540	1	10	3	4	7	6	9	8	11	5	2
C03251	1	2	9	4	10	3	8	6	5	7	11
C01662	1	2	10	9	4	5	8	11	6	3	7
C08129	1	3	2	5	11	8	6	4	9	10	7
C04416	8	3	2	4	5	9	11	7	1	10	6
C08605	8	9	4	1	10	6	3	5	11	2	7
C07443	11	5	10	1	3	8	9	2	6	4	7
C05371	2	10	11	8	5	7	1	3	6	9	4
C09732	10	9	5	1	8	2	7	4	3	11	6
C02924	6	1	5	2	8	11	9	7	4	3	10
C08588	8	9	10	1	5	4	11	6	3	2	7
C06690	3	10	8	5	11	9	7	6	1	4	2
C11052	11	3	8	10	1	2	6	9	4	5	7

C01930	5	6	8	10	11	2	1	3	4	9	7
C10736	5	10	9	8	4	3	2	11	6	1	7
C14186	11	2	4	1	5	8	3	9	6	7	10
C11839	5	2	1	10	8	11	4	3	7	9	6
C08002	10	6	9	4	11	2	1	7	3	8	5
C10972	11	5	6	4	2	8	10	3	9	7	1
C09750	9	3	2	10	8	1	5	11	7	6	4
C15516	2	3	8	6	9	5	11	10	4	7	1
C01796	1	2	8	5	3	10	6	7	9	11	4
C07904	5	9	8	1	10	4	3	11	2	7	6
C06897	10	9	5	6	4	2	8	3	7	11	1
C02454	11	5	1	6	8	9	3	7	2	10	4
C09265	10	11	5	6	3	7	9	2	1	4	8
C06489	10	11	3	5	2	6	9	1	8	7	4
C08424	10	5	11	7	3	1	4	9	8	6	2
C09205	9	3	5	6	2	8	4	10	1	11	7
C14708	11	4	5	6	1	10	3	8	7	2	9
C10798	11	5	4	2	8	6	9	3	7	1	10
C12712	11	10	1	2	3	6	5	4	7	8	9
C04017	1	6	5	3	11	2	9	10	7	8	4
C12102	11	5	1	10	9	4	8	2	6	3	7
C03733	1	4	9	3	8	6	10	5	7	11	2
C09793	10	5	1	9	6	11	8	2	7	4	3
C00349	6	8	2	5	1	11	9	3	4	10	7
C11840	8	5	2	11	9	1	6	3	10	7	4
C09149	10	3	1	5	4	8	2	11	7	6	9
C11738	5	11	10	3	8	1	2	4	6	9	7
C11806	10	1	9	4	5	2	11	7	8	3	6
C09204	11	4	3	9	8	1	10	2	6	5	7
C07578	11	10	4	2	1	6	5	8	3	7	9
C00238	1	5	2	6	8	4	10	3	11	9	7
C11159	1	2	11	8	5	6	10	9	4	3	7
C07116	5	10	8	4	6	11	9	7	2	3	1
C01552	2	10	5	3	11	4	8	1	6	7	9
C15570	10	11	5	6	4	8	3	9	1	2	7
C09976	1	3	9	10	8	11	7	5	4	6	2
C08051	11	5	9	1	8	4	7	6	10	3	2
C10970	5	9	10	8	4	2	11	1	3	7	6
C10070	9	3	8	1	10	4	11	7	6	2	5
C15435	1	9	10	2	7	5	4	6	8	11	3
C11589	3	6	7	10	1	11	5	9	2	4	8
C11266	4	3	9	10	2	5	6	11	8	1	7
C10519	10	7	8	1	6	3	11	5	2	4	9

C08099	10	9	6	5	4	8	11	7	1	3	2
C08313	5	2	8	11	6	10	1	3	9	7	4
C14207	3	1	10	6	11	8	5	7	9	2	4
C10293	11	3	7	1	10	2	8	9	5	4	6
C11048	5	10	2	8	3	11	9	4	1	6	7
C09913	9	11	3	10	5	6	8	1	2	4	7
C09950	11	2	8	6	9	10	7	3	4	1	5
C01925	10	1	9	7	11	3	8	2	6	5	4
C08302	5	10	1	7	11	3	8	4	2	9	6
C12288	9	10	11	3	1	6	7	4	8	2	5
C15485	1	5	2	8	6	11	4	7	10	3	9
C12168	10	5	1	2	6	11	9	3	4	7	8
C04420	1	10	6	7	9	2	8	3	11	4	5
C06939	10	1	5	6	2	4	8	11	9	7	3
C11160	11	10	2	8	5	4	6	1	9	3	7
C15469	11	8	10	5	1	9	3	2	6	4	7
C08697	9	10	11	5	4	1	7	3	6	8	2
C15589	10	11	5	3	2	1	9	6	8	7	4
C00824	8	2	5	1	6	11	9	4	10	3	7
C06123	3	6	2	8	7	1	9	5	11	4	10
C09911	9	3	7	2	5	6	11	10	4	8	1
C07014	9	3	5	1	8	11	10	2	4	7	6
C03228	11	6	8	5	7	3	1	4	2	9	10
C10682	10	2	8	3	11	5	6	4	1	9	7
C14633	11	6	8	5	7	10	3	2	1	4	9
C05353	1	2	4	5	11	10	8	6	3	7	9
C13037	3	9	6	2	1	7	8	5	4	11	10
C06414	8	2	4	11	7	1	3	9	10	5	6
C12491	5	10	6	2	8	11	9	1	3	7	4
C11126	9	11	2	10	8	4	5	6	3	7	1
C06888	9	10	8	2	3	7	6	11	5	4	1
C06889	9	10	5	6	11	7	2	4	1	8	3
C01421	1	4	9	3	10	7	6	2	8	11	5
C08918	3	9	10	6	1	2	8	4	7	5	11
C07312	4	11	9	1	6	5	2	3	7	8	10
C07187	5	11	10	4	9	2	3	7	1	8	6
C04054	2	8	10	3	7	9	4	6	1	11	5
C07899	5	1	10	7	2	9	6	11	3	4	8
C11790	10	11	5	6	1	3	7	4	8	9	2
C13833	3	5	2	4	7	8	1	11	10	9	6
C02200	1	10	9	7	4	3	8	2	11	5	6
C08324	10	3	8	5	1	4	11	6	9	7	2
C14764	5	10	9	6	11	7	4	8	3	1	2

C14594	3	1	2	9	5	11	4	10	6	8	7
C14011	1	6	5	7	4	8	9	10	11	3	2
C09482	4	10	1	9	7	11	8	5	3	6	2
C09798	9	10	3	5	8	6	4	2	7	11	1
C10826	3	9	10	5	7	2	6	11	8	1	4
C10029	10	11	7	2	6	4	3	1	9	8	5
C07417	4	6	2	5	10	9	3	1	11	7	8
C03030	4	6	8	10	1	5	2	11	7	3	9
C09554	3	2	1	10	9	6	8	11	5	4	7
C00182	1	2	5	3	9	6	4	8	11	10	7
C00659	5	1	8	6	2	11	9	3	10	4	7
C02564	2	8	6	4	3	5	10	7	9	1	11
C14328	11	2	3	8	10	7	1	9	6	5	4
C02522	1	2	5	10	3	6	8	9	4	11	7
C12717	10	4	8	9	5	7	3	11	1	2	6
C03098	10	9	11	5	6	7	3	8	4	1	2
C11263	9	1	3	5	11	4	8	10	6	7	2
C05364	11	8	2	6	7	10	3	1	5	9	4
C06810	4	6	11	9	5	2	1	10	8	3	7
C08615	3	10	9	11	1	5	2	4	8	7	6
C10258	9	8	3	6	4	10	1	5	11	7	2
C14438	11	1	3	10	2	4	8	9	5	7	6
C01924	5	6	10	2	8	11	1	7	4	9	3
C02572	5	8	10	3	1	6	11	2	7	4	9
C13700	3	11	5	8	1	10	9	6	7	2	4
C09123	10	7	5	1	9	2	6	3	11	8	4
C06980	5	10	1	11	2	4	9	3	8	6	7
C02080	2	5	11	4	6	1	7	10	9	3	8
C11152	2	5	1	11	6	3	10	4	8	9	7
C16444	11	6	1	8	5	3	9	4	2	7	10
C14333	9	11	1	8	2	5	7	3	6	4	10
C02356	5	8	2	6	1	10	11	9	4	7	3
C06921	11	5	3	10	6	4	1	9	7	8	2
C01023	10	9	11	6	5	3	7	1	4	8	2
C03848	1	4	9	3	10	2	5	8	6	11	7
C04733	1	11	2	6	8	10	4	3	9	7	5
C10894	5	2	11	7	9	3	10	6	4	8	1
C01688	10	1	9	7	11	3	8	2	5	6	4
C11212	10	6	1	5	7	2	3	4	11	8	9
C11380	8	5	10	2	11	6	3	1	9	4	7
C09375	5	10	2	7	4	8	11	3	1	9	6
C09195	5	10	11	9	4	7	3	6	2	1	8
C01905	5	6	2	1	10	8	4	9	11	7	3

C07637	10	4	1	2	6	11	5	3	7	8	9
C04322	5	6	8	2	1	10	4	3	11	9	7
C10874	10	9	8	2	6	11	3	1	5	4	7
C06222	2	1	7	8	3	10	5	9	11	4	6
C08613	9	1	8	2	5	4	10	11	3	7	6
C11710	10	5	11	3	1	2	6	7	8	9	4
C07350	2	6	5	9	3	1	4	11	8	7	10
C13840	3	2	9	4	7	11	5	10	6	8	1
C09435	9	10	4	2	8	1	7	3	5	11	6
C04333	8	2	5	7	10	3	6	11	1	9	4
C10367	11	9	8	6	10	5	4	3	2	7	1
C14752	3	10	11	9	2	8	6	7	5	1	4
C07450	5	10	11	2	1	4	6	3	8	7	9
C11623	3	1	11	4	10	2	7	8	5	6	9
C11185	3	1	6	8	9	10	5	4	2	11	7
C06970	5	9	3	10	2	6	1	11	4	7	8
C09973	11	5	10	1	9	2	7	3	8	6	4
C07196	5	10	4	8	11	3	1	9	6	2	7
C02453	2	7	4	3	8	11	5	6	1	9	10
C11175	6	3	5	2	9	10	4	7	1	8	11
C06902	1	11	5	10	2	8	4	9	6	3	7
C02193	2	4	8	1	10	5	3	9	7	11	6
C07560	11	10	5	4	7	1	2	3	8	9	6
C08756	10	7	11	2	3	4	5	8	6	1	9
C00835	8	5	2	6	10	4	1	7	11	9	3
C15495	2	5	4	8	11	6	10	1	7	9	3
C09761	10	6	1	5	8	11	9	4	7	3	2
C09547	10	11	3	2	9	6	8	4	5	1	7
C11100	3	9	4	10	7	2	5	11	6	8	1
C09910	1	9	6	4	11	2	3	10	5	7	8
C06534	10	11	8	5	1	9	3	7	4	2	6
C08676	10	9	7	1	6	8	11	3	4	2	5
C09674	8	7	2	3	6	10	11	9	5	4	1
C12155	3	10	8	9	2	7	5	11	6	4	1
C10446	10	5	3	8	9	11	2	1	6	4	7
C12189	1	10	11	5	7	2	9	6	4	3	8
C09230	5	9	3	8	10	7	2	4	1	6	11
C15628	3	9	10	1	5	7	8	6	2	11	4
C00891	2	8	6	7	1	3	5	4	11	10	9
C06500	1	2	5	4	6	9	3	11	7	8	10
C04093	9	2	8	3	5	4	11	1	7	6	10
C04316	11	3	5	1	2	6	9	8	4	7	10
C09635	9	1	2	3	11	7	5	4	8	10	6

C09833	10	9	3	11	5	2	1	7	4	6	8
C10480	9	10	11	3	5	8	2	1	7	4	6
C01125	2	6	8	10	11	3	4	7	1	5	9
C10836	10	11	5	8	9	6	4	3	7	2	1
C07044	4	10	11	9	8	5	6	2	1	7	3
C06817	11	5	3	9	10	6	8	1	7	4	2
C06669	2	5	10	4	6	8	9	3	11	7	1
C09171	11	3	9	2	6	4	1	7	10	8	5
C04516	1	10	5	11	9	3	8	7	4	2	6
C02338	1	2	5	10	3	6	8	9	4	11	7
C11268	10	9	5	6	8	11	2	1	3	4	7
C01711	1	8	2	10	6	3	11	5	4	7	9
C14513	11	10	9	5	1	2	6	3	7	8	4
C08630	3	9	10	2	1	6	7	11	5	8	4
C15471	5	2	9	6	8	11	1	4	7	10	3
C08219	5	2	6	10	11	1	4	8	9	3	7
C08315	3	10	1	5	9	11	7	6	8	2	4
C11184	4	10	9	6	5	1	8	3	11	2	7
C14260	11	6	5	3	4	8	2	9	10	1	7
C08431	4	11	9	2	5	10	3	8	1	6	7
C01478	1	2	6	8	5	4	11	9	10	7	3
C08159	3	11	5	9	10	8	7	1	6	4	2
C00175	1	8	5	2	6	11	4	9	10	3	7
C06804	1	11	5	10	3	2	8	6	9	4	7
C01484	10	11	5	3	2	9	6	1	8	4	7
C13182	5	11	4	10	3	8	7	6	9	1	2
C11750	5	9	10	4	3	2	8	11	1	7	6
C09521	9	11	7	4	2	6	1	3	8	5	10
C01821	10	5	9	2	8	11	1	4	7	3	6
C02677	6	5	9	4	10	7	11	3	1	2	8
C04022	2	5	8	11	3	4	6	1	10	7	9
C03930	3	2	10	5	8	1	6	4	9	11	7
C10794	10	5	8	2	6	1	3	9	11	4	7
C03256	11	5	1	8	10	6	3	4	9	7	2
C13676	6	8	2	10	1	5	11	3	7	9	4
C03495	4	2	9	8	10	5	1	7	11	3	6
C08171	11	9	5	6	3	8	7	2	1	4	10
C02543	1	8	2	3	10	9	4	5	11	6	7
C14680	3	5	8	9	6	1	11	2	4	10	7
C08146	10	9	5	11	6	1	4	8	2	3	7
C07868	10	5	11	3	9	8	2	4	7	1	6
C15475	5	3	10	11	8	9	2	1	7	4	6
C13109	10	5	6	8	3	11	1	2	4	7	9

C12628	1	7	6	11	3	10	2	9	8	4	5
C09339	10	8	1	11	9	7	2	4	3	6	5
C04514	11	8	2	5	1	4	3	9	6	10	7
C14521	11	5	9	7	10	3	4	8	6	1	2
C10532	10	5	8	6	9	7	3	4	2	11	1
C12232	10	4	1	7	9	8	11	3	6	2	5
C00420	1	2	9	4	10	3	11	7	8	6	5
C05482	3	2	4	9	1	11	5	7	6	8	10
C10311	11	2	9	10	7	3	5	8	1	6	4
C08525	10	3	1	8	2	7	6	11	9	4	5
C00727	11	5	2	1	8	4	9	6	10	7	3
C14362	11	2	1	5	4	3	8	9	10	6	7
C07615	5	6	10	4	9	11	3	7	1	2	8
C10995	11	5	10	9	4	2	1	7	6	3	8
C09041	5	10	1	9	11	4	2	8	6	3	7
C01029	6	5	10	2	9	1	7	8	11	3	4
C02943	2	6	8	1	7	3	10	9	11	4	5
C11320	5	1	6	2	10	8	4	11	9	3	7
C00396	4	8	6	1	9	11	7	10	2	5	3
C07325	10	5	11	9	8	2	3	7	6	1	4
C09787	10	9	11	1	5	2	8	4	3	7	6
C04611	9	4	5	7	2	1	10	3	6	11	8
C14356	11	5	6	2	10	3	7	9	4	8	1
C07001	10	5	1	2	4	9	11	7	6	3	8
C02099	10	5	8	11	6	1	3	9	2	4	7
C02207	8	2	1	6	4	9	11	5	7	3	10
C14529	11	3	4	7	1	6	9	2	5	10	8
C06965	10	5	2	9	1	11	4	3	7	6	8
C07790	5	10	1	2	4	11	7	6	3	8	9
C10788	10	11	5	8	9	3	6	1	2	4	7
C09251	10	9	11	3	4	5	8	6	2	1	7
C07069	11	4	10	1	8	6	3	2	5	7	9
C01009	1	7	9	10	4	3	8	6	2	11	5
C09156	5	10	2	6	4	8	11	3	7	1	9
C07629	3	9	10	4	8	1	11	2	5	6	7
C10982	11	9	10	2	1	3	5	8	7	4	6
C15109	10	5	11	3	7	9	6	1	2	4	8
C07666	11	10	8	9	2	5	6	7	1	4	3
C04610	1	2	5	3	6	8	7	10	9	4	11
C07065	4	6	2	9	8	11	5	1	10	3	7
C10554	3	5	1	6	7	8	10	9	11	2	4
C12255	1	11	8	4	10	3	2	5	7	9	6
C10312	11	1	3	10	5	8	9	4	6	2	7

C09599	10	7	4	2	8	3	5	6	1	9	11
C03621	11	4	2	5	1	9	3	6	10	7	8
C05104	2	3	8	6	9	11	5	1	7	4	10
C13101	3	11	6	2	8	10	9	4	5	1	7
C10033	10	3	9	8	7	5	11	6	2	1	4
C07384	5	6	10	3	7	8	9	2	4	1	11
C05028	3	9	11	2	6	7	1	10	5	8	4
C07220	10	2	5	1	4	11	6	9	7	3	8
C06316	1	8	2	6	11	7	3	5	10	9	4
C14209	3	11	10	5	4	6	1	9	7	2	8
C01397	3	1	10	11	8	6	5	9	2	4	7
C06351	10	5	11	1	3	6	4	8	2	9	7
C04071	4	2	9	10	8	6	3	11	1	7	5
C16454	5	11	6	2	7	8	3	10	9	4	1
C09263	3	10	8	11	5	2	9	6	1	4	7
C07580	10	11	8	3	7	9	2	5	1	4	6
C14508	11	9	6	2	10	8	1	7	3	4	5
C11199	10	9	5	11	6	3	1	8	7	2	4
C09643	10	5	11	6	7	3	1	2	9	8	4
C13138	8	10	9	11	2	6	3	1	5	7	4
C09269	10	11	2	3	1	6	8	5	4	9	7
C08007	10	11	2	8	5	3	4	7	1	6	9
C11510	5	11	9	10	6	3	8	4	7	2	1
C08064	10	3	1	5	8	11	7	6	9	2	4
C11064	11	10	5	1	2	4	3	6	9	8	7
C07403	10	11	5	8	9	2	4	1	6	3	7
C09741	10	1	5	2	9	6	4	3	8	11	7
C06158	1	8	2	4	6	3	11	7	9	5	10
C09176	10	2	1	8	3	7	5	11	9	4	6
C04584	8	5	11	2	10	6	3	9	1	7	4
C09738	9	11	10	6	2	8	3	1	5	4	7
C07415	5	10	11	3	8	4	7	1	2	6	9
C03212	8	2	1	6	5	4	10	7	9	11	3
C04606	2	6	9	8	1	7	11	5	3	4	10
C09366	11	8	5	1	2	4	10	3	7	9	6
C07876	2	1	8	4	5	10	9	6	11	7	3
C07657	10	9	5	6	1	4	7	2	3	8	11
C04209	5	6	8	2	3	10	1	9	4	7	11
C14234	10	3	11	8	2	7	5	1	4	6	9
C09153	3	10	5	7	8	9	2	4	11	6	1
C10848	10	5	1	6	4	7	3	8	11	2	9
C08280	5	6	11	9	8	1	10	3	7	2	4
C02678	3	11	6	5	1	2	9	4	7	8	10

C11586	11	1	8	3	5	9	4	7	10	6	2
C02794	5	8	6	2	10	1	11	7	9	4	3
C07837	1	11	2	8	3	6	9	5	10	7	4
C08245	1	8	2	11	6	7	5	4	10	3	9
C09185	8	1	2	11	5	10	3	6	7	4	9
C06832	5	9	10	11	8	3	2	1	7	4	6
C12179	10	7	11	6	8	1	4	3	9	2	5
C15335	3	6	11	5	4	2	7	1	10	8	9
C02801	2	6	8	7	1	9	10	4	3	5	11
C04590	9	3	2	10	4	8	5	1	11	7	6
C10752	10	6	5	1	8	9	3	4	11	2	7
C14081	3	5	7	10	1	2	11	4	6	9	8
C08468	5	10	6	2	4	8	3	7	1	11	9
C06155	1	2	10	3	8	7	11	9	4	5	6
C14500	3	5	8	4	9	11	1	2	6	7	10
C11776	5	10	11	8	2	3	9	1	4	7	6
C10185	10	1	8	2	5	6	9	3	7	4	11
C10155	10	1	6	7	4	9	11	2	8	3	5
C02904	2	9	8	6	1	3	4	10	11	5	7
C02533	2	11	8	5	3	10	4	7	6	1	9
C11169	9	10	11	8	2	5	3	1	6	7	4
C16185	3	9	5	1	8	10	11	4	7	6	2
C11741	5	10	7	6	3	4	1	8	11	9	2
C10094	2	8	6	11	5	1	4	10	9	7	3
C09920	3	5	1	8	9	2	6	7	4	11	10
C08087	6	8	11	5	3	4	10	9	7	1	2
C11113	4	5	2	9	8	3	1	6	7	10	11
C11147	2	6	1	11	8	10	9	7	5	4	3
C15536	6	5	2	1	3	9	8	11	4	10	7
C10930	11	6	8	4	2	7	9	3	5	1	10
C03107	1	2	8	10	9	6	7	11	4	3	5
C07839	11	3	4	9	1	8	6	7	5	2	10
C13719	10	9	1	5	6	4	3	11	2	8	7
C10212	11	2	5	1	3	7	10	4	8	9	6
C13103	9	1	5	3	6	10	7	11	8	2	4
C08174	3	2	7	9	6	1	10	11	5	8	4
C02722	2	8	7	5	10	1	11	6	3	4	9
C07263	2	6	8	7	11	4	5	1	10	9	3
C10168	10	11	4	9	3	2	6	8	1	5	7
C13127	10	3	9	11	1	7	6	8	2	5	4
C10526	10	5	9	11	2	3	1	7	6	8	4
C02638	6	5	3	2	8	7	9	1	11	10	4
C07064	1	2	5	11	3	6	4	8	9	7	10

C01348	4	10	6	9	8	3	5	2	7	11	1
C11275	11	1	2	5	3	6	4	8	9	7	10
C12158	8	2	5	1	4	7	3	9	6	10	11
C01295	2	6	1	8	4	5	3	11	9	10	7
C10819	9	5	3	8	4	2	10	7	11	1	6
C06683	10	6	9	5	2	8	1	4	11	3	7
C13780	1	2	10	5	11	6	7	3	4	9	8
C01555	9	10	5	6	1	3	11	7	2	8	4
C14339	11	5	8	3	10	7	1	9	4	2	6
C02711	1	5	2	8	10	6	3	11	9	4	7
C08296	1	5	6	2	8	10	4	3	11	9	7
C05613	1	5	6	8	2	10	11	3	4	7	9
C13672	1	2	5	6	9	7	3	4	10	11	8
C02154	1	2	5	8	6	3	9	11	10	4	7
C08778	10	2	3	6	5	8	7	11	4	9	1
C03042	2	8	3	5	1	6	10	4	9	7	11
C06419	5	6	2	1	8	10	4	9	3	7	11
C02390	11	8	6	2	5	1	9	7	10	3	4
C10603	6	10	5	11	8	3	7	9	1	2	4
C16421	5	1	2	10	9	8	3	4	7	6	11
C10354	11	5	4	8	10	6	9	2	3	7	1
C10334	11	10	2	9	1	8	3	6	7	5	4
C14706	11	4	1	5	9	8	10	2	7	6	3
C10273	1	10	6	7	11	5	3	9	2	8	4
C08450	11	10	9	2	1	8	4	6	3	5	7
C08425	10	5	11	2	3	7	4	1	8	6	9
C09567	10	11	2	9	5	8	7	1	3	4	6
C03462	2	10	4	9	6	5	8	1	3	11	7
C14595	11	3	4	9	8	5	6	10	2	1	7
C13765	10	9	1	5	2	4	8	11	6	3	7
C06325	5	8	10	1	2	11	6	9	4	3	7
C02015	8	3	5	1	9	11	2	6	4	10	7
C02781	1	2	10	5	6	8	9	4	3	11	7
C02809	11	9	10	2	5	1	8	3	4	6	7
C12117	1	11	5	9	7	6	8	3	10	2	4
C08880	5	10	1	3	4	9	6	11	7	2	8
C05930	2	6	5	9	10	4	3	1	7	11	8
C14010	10	9	11	6	5	2	1	8	3	7	4
C12862	11	1	9	8	5	3	7	2	4	10	6
C06373	1	2	3	5	8	9	6	4	10	11	7
C07489	11	1	8	5	3	9	10	4	6	7	2
C07105	3	5	1	8	10	4	11	9	7	2	6
C10843	10	3	1	5	9	2	8	11	7	6	4

C04047	1	5	3	11	8	6	2	9	10	7	4
C11526	1	2	4	10	7	6	5	8	9	3	11
C09334	10	8	9	11	6	3	1	7	4	5	2
C14134	3	11	10	1	9	5	7	4	2	8	6
C03474	11	1	4	10	3	8	5	9	2	6	7
C10517	1	9	4	2	3	8	7	6	11	10	5
C08643	1	10	6	4	8	7	9	3	11	2	5
C02778	11	1	4	8	5	6	2	9	3	10	7
C10490	11	9	10	5	4	2	8	6	3	1	7
C03169	4	1	11	2	8	7	9	10	6	5	3
C13688	5	1	8	10	6	9	2	3	11	7	4
C09203	10	5	1	2	6	3	4	8	11	7	9
C08345	6	1	10	5	11	8	4	9	3	7	2
C15479	1	10	7	3	6	4	5	2	9	11	8
C14565	11	9	8	3	2	10	4	5	1	6	7
C02155	5	6	10	8	7	2	1	11	4	3	9
C01844	3	9	5	8	1	2	10	11	6	7	4
C08305	11	1	3	6	4	10	7	5	8	2	9
C07355	3	8	2	4	7	5	11	9	1	10	6
C08294	5	2	9	10	8	11	3	1	7	4	6
C10659	3	10	9	7	5	6	1	2	4	8	11
C10163	11	2	5	1	6	4	10	7	9	3	8
C04217	9	8	2	6	3	5	11	4	1	10	7
C02115	5	8	2	6	7	1	3	4	11	10	9
C00150	2	1	5	11	8	6	4	9	10	7	3
C04625	11	1	9	8	6	10	5	3	2	7	4
C04303	2	1	11	8	5	4	6	10	7	9	3
C10327	11	10	2	6	8	3	1	4	5	9	7
C00820	5	1	6	2	8	10	9	4	3	11	7
C08838	9	3	4	6	10	2	1	5	8	11	7
C05241	4	5	2	11	10	6	1	8	3	7	9
C10608	11	8	9	5	10	6	3	4	2	7	1
C10706	10	9	11	5	4	6	1	3	7	8	2
C08163	3	11	9	5	2	7	1	10	4	8	6
C07445	5	10	11	3	4	8	9	1	7	6	2
C02866	1	3	8	6	10	5	9	4	11	2	7
C06522	5	6	10	8	2	11	1	3	4	7	9
C10764	10	5	2	8	7	9	4	3	11	1	6
C11308	1	11	5	3	2	6	8	7	9	10	4
C05377	8	1	2	11	6	5	3	10	9	4	7
C08493	5	11	10	6	8	2	1	9	4	3	7
C11186	2	5	4	6	11	3	9	8	1	7	10
C04197	1	10	9	4	3	5	8	2	11	6	7

C10117	3	8	11	5	4	2	6	7	1	10	9
C07023	10	5	11	9	8	2	1	4	3	6	7
C06852	5	10	3	6	7	2	4	1	11	8	9
C01389	9	8	3	4	2	10	5	1	6	11	7
C11502	8	2	11	5	6	7	9	10	3	4	1
C09868	9	2	8	6	11	5	3	10	7	4	1
C10784	1	5	11	6	4	2	7	8	10	9	3
C08197	10	5	11	2	3	8	6	4	7	1	9
C08201	1	3	2	10	5	6	9	8	7	11	4
C07773	10	11	8	1	2	9	4	6	7	3	5
C13505	6	10	1	5	8	2	9	11	3	7	4
C15603	11	8	1	2	5	10	6	9	4	3	7
C07505	11	10	6	5	1	8	2	7	4	9	3
C01849	9	8	2	1	3	11	10	4	6	7	5
C11153	9	10	11	4	8	1	3	6	5	7	2
C12347	4	2	9	7	6	3	1	5	11	8	10
C08766	1	3	10	4	2	11	5	9	6	7	8
C10987	4	2	7	11	9	8	5	1	6	10	3
C11345	5	2	1	4	8	3	10	9	7	6	11
C10128	10	8	11	5	1	6	4	7	3	9	2
C10781	10	2	11	5	3	6	4	9	1	7	8
C07457	3	10	2	6	5	9	8	4	7	11	1
C08217	5	8	3	6	9	2	11	1	4	10	7
C12446	9	7	5	11	6	8	3	2	4	10	1
C04105	11	8	2	5	1	10	9	4	7	3	6
C10321	10	6	9	11	4	5	1	3	7	8	2
C09120	10	9	1	7	6	8	3	11	5	2	4
C10347	10	5	7	1	3	2	6	8	9	11	4
C03711	5	9	11	2	8	10	4	3	7	1	6
C09259	10	6	5	8	2	1	11	9	3	4	7
C07438	5	10	1	11	9	4	8	6	3	7	2
C02019	11	2	1	8	5	10	9	3	6	4	7
C01572	10	11	8	2	5	1	4	9	3	7	6
C14491	3	6	7	4	2	11	1	5	10	8	9
C14687	1	2	5	11	3	6	9	8	7	10	4
C09840	9	11	10	5	3	6	1	8	2	4	7
C10319	10	9	11	1	6	4	3	8	2	5	7
C07324	10	1	11	9	3	2	5	4	6	8	7
C03527	2	11	8	1	9	6	5	4	10	3	7
C12637	10	2	4	1	11	3	6	8	9	7	5
C02791	11	3	8	9	1	5	6	10	2	7	4
C08057	6	5	8	1	2	9	7	11	4	10	3
C00317	1	9	10	4	2	3	5	7	8	11	6

C12796	9	10	3	5	11	8	4	1	2	7	6
C07437	11	4	9	1	10	3	6	7	5	8	2
C02948	11	3	8	5	6	10	9	2	4	1	7
C07554	10	2	5	9	1	8	7	4	3	6	11
C09207	6	10	5	4	3	1	8	9	7	11	2
C10704	11	9	10	8	6	7	3	1	5	4	2
C07581	8	5	2	6	10	4	11	1	7	9	3
C08176	3	9	6	8	4	11	2	10	5	1	7
C01590	2	10	8	6	5	9	1	4	11	3	7
C03537	6	3	5	9	11	8	2	1	4	10	7
C10301	10	9	1	6	3	11	8	2	7	4	5
C03032	10	1	5	2	4	3	11	9	6	8	7
C10981	11	3	1	10	2	7	6	8	9	4	5
C15562	5	11	2	10	6	8	1	4	3	9	7
C03685	9	6	2	1	7	4	3	10	8	5	11
C07530	5	11	10	9	6	4	7	1	3	2	8
C12167	10	11	2	5	3	7	1	8	9	4	6
C10263	10	5	6	7	4	1	3	9	2	11	8
C01853	1	11	2	5	4	6	8	9	10	3	7
C13326	1	11	5	4	8	2	10	9	6	3	7
C06855	10	5	11	8	2	1	3	4	7	9	6
C06722	2	8	11	1	5	6	7	4	3	9	10
C00959	3	1	4	5	8	6	10	7	11	2	9
C10590	1	9	10	4	3	2	8	5	6	7	11
C11079	11	10	9	2	5	4	8	6	3	1	7
C15488	11	1	9	3	2	8	6	5	4	10	7
C10396	10	1	6	11	9	4	8	3	5	7	2
C07817	3	5	2	4	1	6	10	9	11	7	8
C10937	11	10	5	2	7	6	4	1	8	3	9
C08764	10	6	11	8	4	3	1	7	5	9	2
C13757	6	11	2	9	1	3	8	10	7	5	4
C14221	10	5	6	9	3	4	8	7	11	1	2
C06121	3	7	6	8	4	5	11	10	2	9	1
C13927	6	5	8	2	10	1	9	11	4	3	7
C10770	11	6	10	4	8	7	5	3	1	9	2
C07525	8	5	10	6	11	1	3	2	9	4	7
C07404	5	10	9	11	2	4	3	1	6	8	7
C14606	3	4	2	6	7	10	11	1	8	9	5
C14227	11	3	4	7	2	10	6	1	8	5	9
C07381	10	1	8	6	11	9	4	3	2	5	7
C12311	1	11	5	9	2	6	4	10	3	8	7
C09524	2	10	9	8	6	5	4	3	1	11	7
C03919	4	8	6	2	1	11	5	9	10	3	7

C00808	9	8	2	11	5	6	1	3	10	4	7
C07533	5	10	1	4	2	6	8	11	3	9	7
C13077	3	5	9	8	4	6	2	11	7	1	10
C09359	9	6	1	4	7	11	5	8	3	10	2
C11182	11	5	1	4	10	6	8	2	3	9	7
C07413	3	4	1	2	7	11	5	8	10	6	9
C14432	11	5	9	8	1	10	6	3	2	7	4
C06944	3	5	11	4	2	6	10	1	8	7	9
C12231	10	6	4	1	9	2	8	3	7	5	11
C12942	1	2	5	8	11	10	6	3	4	9	7
C16294	10	8	4	6	1	5	2	7	9	11	3
C09387	10	5	2	9	7	6	3	4	1	8	11
C09737	8	2	3	9	10	4	11	7	1	5	6
C02498	11	9	3	2	8	1	6	5	7	4	10
C11769	5	4	10	9	6	1	11	3	2	7	8
C00738	1	2	10	5	6	8	3	4	11	9	7
C08065	9	11	5	3	7	4	2	1	8	6	10
C09628	9	2	4	6	5	3	8	10	11	7	1
C10204	10	7	3	9	4	6	11	8	1	5	2
C00451	1	2	5	8	6	11	3	4	9	10	7
C14150	11	2	5	3	6	4	8	1	10	9	7
C08233	9	3	2	10	5	11	4	7	8	6	1
C05003	1	2	5	6	8	3	10	11	9	4	7
C10842	10	5	3	11	9	2	8	1	4	7	6
C14476	10	9	3	11	8	5	2	4	1	7	6
C10890	3	5	4	6	7	10	11	8	2	9	1
C11248	9	10	11	1	7	3	2	6	4	5	8
C09257	10	1	9	2	3	7	4	11	8	5	6
C15658	9	10	6	5	11	1	8	2	7	3	4
C15233	1	5	2	8	6	11	10	4	9	3	7
C11181	1	5	2	4	6	11	10	9	8	3	7
C15609	2	8	6	11	3	1	4	9	7	5	10
C09788	9	10	5	11	8	2	6	3	7	4	1
C08858	10	3	9	4	8	6	2	1	7	11	5
C02632	5	1	2	11	9	6	8	10	4	3	7
C10918	11	5	10	2	7	9	8	6	4	3	1
C14441	1	11	3	8	6	2	5	10	4	9	7
C14354	11	8	9	2	1	3	10	7	4	6	5
C07205	3	2	9	4	11	8	6	10	5	7	1
C10115	10	1	6	11	2	7	3	9	4	5	8
C11622	8	2	10	6	4	1	9	5	3	11	7
C08988	10	9	5	11	3	1	8	2	6	4	7
C10276	10	5	9	1	2	8	7	11	6	3	4

C11629	3	8	2	9	5	6	7	4	11	1	10
C11688	10	5	11	3	8	4	1	9	7	6	2
C07609	10	5	9	6	2	8	11	3	4	1	7
C01452	1	2	6	8	5	9	10	11	4	3	7
C04548	5	10	3	11	9	8	4	7	1	6	2
C09232	2	6	7	8	11	9	3	5	10	1	4
C13310	1	3	10	4	8	11	2	9	6	7	5
C10314	11	10	5	9	3	4	7	1	2	6	8
C01937	11	4	8	2	5	1	3	9	6	10	7
C08745	10	5	11	2	4	3	6	8	7	1	9
C12233	10	6	9	1	2	3	4	11	8	7	5
C10832	10	8	11	5	6	9	1	3	2	4	7
C10093	10	3	2	6	9	5	11	7	4	8	1
C10317	1	3	4	2	7	10	9	6	8	11	5
C02578	1	10	11	5	7	6	8	2	4	9	3
C10792	11	5	8	1	10	6	4	3	9	2	7
C01621	1	10	6	2	3	8	11	9	4	7	5
C15629	5	3	9	8	6	11	2	1	4	10	7
C10284	10	2	5	3	1	7	6	9	11	8	4
C03049	1	9	4	3	6	11	5	10	2	8	7
C07340	1	3	5	2	7	8	10	4	6	11	9
C11110	11	9	1	10	3	8	2	5	7	4	6
C09196	10	9	4	2	11	6	7	3	5	8	1
C10309	8	2	9	4	10	7	1	11	3	6	5
C01448	2	8	6	11	9	5	4	7	10	3	1
C11837	5	6	11	3	1	9	2	10	4	8	7
C01950	8	11	3	2	6	5	10	9	1	4	7
C15618	5	2	6	8	4	3	7	10	1	9	11
C14138	11	2	10	9	5	1	4	3	6	8	7
C08113	10	9	6	5	11	7	3	2	4	8	1
C07765	11	5	4	3	8	7	10	6	1	9	2
C02975	2	6	8	11	5	3	10	7	1	4	9
C08819	9	3	6	8	2	10	4	11	5	1	7
C08091	11	4	2	10	9	3	1	5	6	8	7
C02822	2	4	7	8	3	5	1	9	6	11	10
C10040	10	1	3	8	4	9	11	2	6	7	5
C04067	2	6	8	10	4	1	3	9	11	7	5
C13769	10	11	3	8	9	5	2	7	4	1	6
C06467	1	2	10	5	6	8	3	4	11	9	7
C07463	5	9	3	10	11	4	2	6	7	1	8
C14972	3	8	4	9	11	7	10	6	2	1	5
C06885	10	9	6	5	7	8	4	3	11	1	2
C08166	3	11	9	10	6	5	2	7	4	1	8

C14349	11	5	8	4	9	6	3	1	7	2	10
C08908	10	1	11	4	2	7	8	3	5	6	9
C04524	2	11	8	1	3	7	9	5	4	6	10
C08278	5	1	11	3	8	2	9	6	4	7	10
C14230	11	5	3	9	1	6	8	2	4	10	7
C11723	11	3	10	5	9	1	4	8	6	2	7
C11488	2	6	1	11	10	3	9	7	4	8	5
C10796	9	10	4	8	2	3	1	7	6	11	5
C07124	3	5	8	7	11	9	10	1	4	2	6
C11090	11	3	10	1	2	9	5	6	4	8	7
C06688	9	10	11	8	6	3	4	1	5	7	2
C10809	11	10	5	2	8	6	1	4	9	3	7
C09231	9	11	6	10	4	3	7	2	8	1	5
C04456	1	2	9	4	10	11	6	3	8	5	7
C06523	10	5	6	2	8	9	3	11	7	1	4
C09037	10	5	8	7	2	3	6	11	9	1	4
C03870	10	1	2	9	8	3	4	5	11	6	7
C10034	10	8	5	2	11	1	6	4	3	7	9
C04380	4	1	2	9	11	5	10	3	8	7	6
C07736	3	9	2	5	1	11	6	7	8	4	10
C11594	8	4	5	2	3	1	11	7	9	6	10
C00597	1	2	5	8	6	4	3	11	9	10	7
C00825	10	3	6	9	8	2	11	1	5	4	7
C06695	11	5	2	4	3	6	8	7	9	10	1
C01816	11	9	2	10	3	6	1	7	5	8	4
C05201	5	10	9	11	1	7	2	8	3	6	4
C14060	10	9	3	7	4	11	6	1	5	2	8
C10195	10	5	11	8	4	2	9	6	7	3	1
C07818	10	11	3	8	9	1	2	7	5	4	6
C04290	9	10	5	2	8	6	3	4	7	11	1
C10428	9	5	10	11	4	1	8	2	7	3	6
C03015	6	2	5	8	4	11	1	9	7	3	10
C14197	5	6	4	9	1	3	7	10	11	8	2
C00326	1	3	2	7	4	8	10	5	11	9	6
C04867	2	10	8	6	7	11	9	3	1	5	4
C10606	10	9	3	6	11	4	8	7	2	5	1
C03709	2	10	5	9	4	1	6	3	11	7	8
C10413	10	9	6	5	2	11	8	4	7	1	3
C07318	10	9	11	5	8	6	3	2	7	1	4
C01691	5	11	10	9	1	3	8	2	4	6	7
C13391	3	2	1	7	8	5	11	10	9	6	4
C07875	11	10	7	6	9	2	8	3	5	4	1
C07547	10	5	6	2	8	1	11	3	4	9	7

C07063	5	10	3	2	1	9	8	4	11	6	7
C12568	1	11	2	5	3	6	4	10	9	7	8
C03422	3	7	8	9	10	2	5	11	6	4	1
C15758	9	6	5	8	4	3	1	11	2	7	10
C01926	3	1	10	2	5	8	6	4	9	11	7
C14161	9	1	2	5	8	11	6	4	10	3	7
C10294	10	11	2	5	1	6	3	7	9	8	4
C07309	10	5	1	4	3	9	2	8	6	11	7
C03799	4	1	9	3	10	2	6	5	11	8	7
C01390	9	1	2	8	7	3	10	11	4	5	6
C09076	3	10	5	2	1	4	6	8	9	11	7
C02122	5	11	1	3	9	2	8	6	4	7	10
C10141	1	10	11	2	4	6	9	7	5	3	8
C08411	10	5	3	4	9	8	11	6	7	1	2
C08804	6	10	9	4	8	3	2	11	7	5	1
C10025	10	3	5	11	2	8	9	1	4	6	7
C15449	2	11	9	8	10	3	6	1	5	4	7
C08398	11	4	1	2	8	3	10	5	6	9	7
C11631	8	2	4	11	7	9	5	3	1	10	6
C07642	11	10	9	4	2	5	1	8	3	7	6
C09065	3	1	9	4	2	11	6	7	5	10	8
C14506	9	3	11	5	6	2	1	8	4	10	7
C07494	11	6	10	2	5	1	3	8	9	7	4
C07559	5	10	3	8	1	7	2	11	4	9	6
C03045	2	8	3	5	1	6	10	11	4	7	9
C10487	10	11	8	7	4	6	3	5	1	9	2
C04199	1	9	4	3	2	8	7	11	5	6	10
C11154	11	8	4	2	5	9	3	10	1	6	7
C08659	1	5	9	10	11	6	8	4	2	3	7
C05819	1	2	11	5	8	3	6	9	4	7	10
C09497	9	8	11	5	6	4	2	3	10	1	7
C12098	5	11	8	1	3	2	7	10	9	4	6
C10852	10	5	4	9	8	7	3	2	11	1	6
C00977	2	5	10	1	4	6	8	9	3	11	7
C14357	11	10	5	1	2	4	9	8	6	3	7
C10671	3	7	9	4	10	11	6	1	5	8	2
C11121	3	9	11	8	2	6	5	10	7	1	4
C03779	11	2	8	7	4	3	9	1	5	10	6
C14169	10	9	11	2	8	3	1	6	5	7	4
C02779	1	8	2	10	7	9	6	3	5	4	11
C04427	1	2	9	8	10	7	4	5	3	6	11
C14239	11	2	3	6	4	9	7	1	10	8	5
C06413	11	5	8	2	10	6	4	9	3	1	7

C10753	5	7	10	8	3	9	2	1	6	11	4
C08244	10	1	6	7	2	8	3	5	4	11	9
C10597	10	9	4	11	1	5	3	2	6	7	8
C11235	11	1	3	5	2	8	4	6	10	9	7
C07805	10	5	4	2	9	8	11	7	1	3	6
C10789	10	8	5	7	9	2	1	3	6	4	11
C15584	11	1	8	5	10	2	3	6	4	9	7
C01359	5	11	8	2	1	10	7	4	3	6	9
C03081	1	3	11	2	9	8	5	4	10	7	6
C11233	11	2	4	10	9	5	3	1	8	6	7
C13008	10	11	5	1	8	2	9	3	4	6	7
C14698	11	8	3	5	4	2	10	1	7	6	9
C09877	9	8	2	11	3	1	7	5	10	4	6
C09537	9	8	5	7	6	4	3	1	11	2	10
C09142	10	9	5	3	4	6	8	2	7	11	1
C03668	1	5	2	6	8	3	11	9	10	4	7
C08382	5	2	7	4	6	10	3	9	1	11	8
C16245	1	2	4	7	5	10	8	11	9	3	6
C14383	3	4	8	2	6	5	7	11	9	10	1
C07881	10	11	5	9	1	2	6	7	3	4	8
C15568	2	8	7	9	5	4	3	11	10	1	6
C15736	10	1	4	8	7	3	6	9	5	11	2
C10454	9	11	3	5	10	8	4	7	2	1	6
C01684	1	2	5	10	3	6	8	9	4	11	7
C07650	4	11	9	8	10	2	6	5	3	7	1
C06685	3	10	1	5	9	6	4	2	11	8	7
C07807	5	10	2	8	6	4	1	9	3	11	7
C05147	5	1	8	2	10	11	6	4	3	7	9
C01487	1	2	10	5	6	8	3	4	11	9	7
C12229	10	3	6	9	4	1	11	2	7	5	8
C12600	1	11	3	5	10	6	9	2	8	4	7
C12099	11	5	8	9	4	7	1	10	3	2	6
C10847	10	11	1	2	8	7	6	5	4	9	3
C14321	11	9	5	8	3	2	6	10	4	1	7
C03210	5	1	6	11	2	8	9	7	3	4	10
C07202	10	5	11	1	3	8	2	4	7	9	6
C01002	1	2	4	9	10	8	3	7	6	5	11
C03001	5	8	6	2	10	11	7	3	4	1	9
C10688	5	10	9	2	4	8	7	3	6	1	11
C12893	1	2	11	4	6	9	3	7	5	10	8
C14449	11	2	4	1	5	6	10	8	9	3	7
C10074	9	10	7	5	4	11	2	3	1	8	6
C14410	5	10	4	2	9	11	3	6	7	8	1

C11704	3	10	9	5	11	1	2	8	4	7	6
C08142	1	2	4	11	10	8	5	3	6	9	7
C02997	5	6	3	8	2	1	11	9	10	4	7
C03354	5	1	11	6	10	2	4	8	3	9	7
C14184	11	3	6	1	10	4	7	2	8	5	9
C03290	5	10	8	6	3	2	11	9	4	7	1
C09158	10	1	7	4	5	11	3	2	8	6	9
C06115	1	4	2	8	9	10	5	6	7	11	3
C09777	9	10	6	11	7	3	1	5	2	4	8
C08461	10	11	6	4	9	7	3	5	1	8	2
C07814	10	1	3	5	4	2	8	7	11	6	9
C10811	10	8	6	11	9	7	2	3	1	5	4
C13772	1	10	2	5	4	6	3	8	11	7	9
C08257	1	10	2	5	8	6	9	3	11	7	4
C01188	5	8	1	3	2	11	10	6	4	9	7
C06420	5	6	8	10	2	1	11	4	9	3	7
C07619	3	2	4	1	5	8	10	6	11	9	7
C11799	4	5	6	1	10	3	8	11	9	7	2
C09630	9	3	8	5	2	4	11	6	7	10	1
C07259	4	3	10	5	2	1	6	8	7	9	11
C11237	3	10	9	6	1	5	2	8	4	11	7
C15476	5	10	2	3	1	4	11	8	6	9	7
C09513	4	3	6	8	10	11	2	1	7	5	9
C11114	11	10	5	1	2	4	7	3	9	8	6
C11163	2	8	4	5	1	7	3	6	10	11	9
C02539	3	11	10	8	1	9	5	7	6	2	4
C08403	10	5	6	7	8	4	2	3	11	1	9
C09883	2	11	10	7	8	4	5	3	6	9	1
C03753	11	5	8	9	10	7	2	3	4	6	1
C14693	11	8	10	5	3	1	9	6	2	4	7
C07422	10	1	5	6	11	3	4	2	8	9	7
C08492	9	11	1	5	3	6	7	2	10	4	8
C13376	6	3	2	9	11	10	4	7	8	5	1
C10026	1	9	4	2	3	10	5	6	8	11	7
C07566	5	10	11	1	3	6	4	2	8	9	7
C08866	11	6	10	3	1	4	8	2	9	7	5
C15561	2	8	1	11	7	6	4	9	5	3	10
C05086	9	11	4	5	1	8	3	6	7	10	2
C09307	1	10	11	3	7	6	4	8	5	2	9
C06840	10	9	6	11	2	5	3	8	4	7	1
C08840	3	10	1	11	5	9	2	6	8	7	4
C07656	10	9	1	5	6	2	11	4	7	3	8
C11161	4	10	9	2	1	5	11	3	8	6	7

C11748	9	10	6	8	1	7	2	3	5	4	11
C09776	10	11	8	5	6	2	9	3	4	1	7
C06544	5	10	6	8	1	4	2	11	9	3	7
C08052	10	9	5	6	11	8	3	4	1	2	7
C02221	11	6	5	1	8	2	7	4	3	9	10
C11485	11	6	1	2	7	4	9	3	10	8	5
C01874	5	11	9	2	10	4	7	8	6	1	3
C06067	9	5	10	2	8	6	7	1	11	3	4
C10102	10	11	2	5	4	1	3	9	6	7	8
C07406	10	11	9	5	2	7	4	1	3	8	6
C11032	11	6	2	9	10	1	3	5	7	4	8
C01357	1	8	2	5	9	11	10	3	6	7	4
C12169	10	1	6	7	2	3	4	11	9	8	5
C07996	10	6	9	1	8	7	3	2	5	11	4
C10952	11	2	6	4	9	3	5	10	7	8	1
C11338	9	11	10	5	8	2	1	6	7	3	4
C08888	1	10	5	8	2	7	3	4	6	11	9
C04840	2	8	5	4	1	11	6	3	7	9	10
C15817	8	2	6	5	11	1	4	9	3	7	10
C04665	2	10	8	1	9	5	7	3	6	4	11
C16453	11	10	9	8	2	6	4	5	1	3	7
C07636	10	9	5	4	1	11	3	7	2	8	6
C03417	3	5	2	10	8	6	4	1	9	11	7
C07491	11	10	2	5	9	1	3	8	6	4	7
C13429	1	5	10	4	6	3	7	8	2	9	11
C10605	5	1	4	3	2	11	6	10	8	7	9
C05458	2	6	3	8	5	9	1	7	11	4	10
C10268	5	9	8	10	6	11	2	1	4	7	3
C07675	3	9	5	8	4	1	2	6	10	7	11
C07421	10	11	1	4	2	3	8	5	6	9	7
C14505	11	1	5	8	9	2	6	10	4	3	7
C06805	1	5	11	2	6	10	3	8	4	9	7
C08813	3	9	7	1	10	6	5	11	4	2	8
C02710	5	10	3	6	1	8	9	2	11	4	7
C08101	10	9	5	11	6	3	8	2	4	1	7
C02026	4	11	9	5	10	8	2	6	3	1	7
C10230	10	11	5	3	6	7	4	2	9	1	8
C13709	4	10	5	2	6	8	9	1	7	11	3
C09779	9	10	1	7	4	6	3	8	11	5	2
C02010	10	2	4	6	5	8	7	9	11	3	1
C01133	2	8	1	5	11	10	6	7	3	4	9
C10840	5	2	6	7	10	9	1	3	8	4	11
C08638	9	3	8	11	1	10	4	2	6	7	5

C07005	3	1	10	8	7	11	5	6	4	2	9
C06786	6	7	2	8	5	4	3	10	11	1	9
C03682	6	8	5	1	10	2	4	3	7	9	11
C10037	10	1	4	5	9	11	2	3	8	6	7
C15588	1	5	2	8	4	3	11	6	10	9	7
C14327	11	10	9	7	2	1	3	4	8	6	5
C10904	10	5	4	9	3	11	8	1	2	6	7
C07898	5	10	2	6	11	1	3	9	8	4	7
C01941	6	10	5	7	2	8	1	11	4	3	9
C14137	10	1	4	9	3	11	7	2	8	5	6
C03887	2	1	4	5	11	8	6	3	10	9	7
C10039	10	5	3	2	7	11	1	4	9	6	8
C08271	5	1	11	6	3	10	8	9	4	2	7
C08162	3	11	5	9	8	1	10	7	4	6	2
C07695	5	6	2	10	9	1	3	4	8	11	7
C02803	11	8	3	5	4	1	2	7	9	10	6
C09638	9	3	1	10	6	8	7	11	2	5	4
C10376	11	4	7	10	3	9	8	2	1	5	6
C09965	10	11	4	5	3	6	8	9	2	1	7
C14273	1	3	11	9	5	4	8	6	10	7	2
C09484	3	2	10	11	6	4	9	7	8	5	1
C10657	1	2	8	6	5	3	4	9	10	11	7
C12187	10	11	2	6	1	3	4	7	9	8	5
C13784	5	3	2	10	6	11	8	7	1	9	4
C10072	10	9	11	8	5	3	4	7	1	6	2
C07956	10	4	9	1	8	11	3	2	7	5	6
C01589	1	5	2	8	3	6	11	10	4	9	7
C11310	5	6	11	10	2	1	8	9	3	4	7
C14256	3	11	8	6	5	2	7	4	1	10	9
C07424	10	5	4	3	11	2	8	9	6	1	7
C02020	2	11	1	8	5	3	6	10	9	7	4
C13711	6	1	5	10	9	7	3	8	2	11	4
C02535	6	5	2	11	10	8	4	7	1	3	9
C09894	11	9	10	2	3	1	5	4	8	7	6
C12040	5	10	4	9	8	1	7	2	3	6	11
C03510	5	6	2	1	8	4	10	9	11	7	3
C05919	8	11	5	9	2	7	3	10	4	6	1
C09376	2	1	9	6	7	4	8	11	5	10	3
C07543	5	10	3	1	8	9	11	2	6	7	4
C07405	5	6	10	2	8	11	3	4	1	9	7
C03343	11	8	3	2	6	7	4	10	1	9	5
C14176	11	10	9	6	3	1	8	5	2	7	4
C14576	11	2	9	4	3	10	8	5	7	6	1

C09771	1	2	5	10	6	9	8	3	11	4	7
C15910	9	1	10	3	8	6	4	5	2	11	7
C14607	3	2	10	8	9	4	1	11	6	7	5
C04229	5	2	10	8	4	3	6	1	9	7	11
C10622	3	10	5	6	4	8	9	2	7	11	1
C11224	10	4	5	9	8	2	3	7	6	1	11
C11601	11	8	9	10	1	2	3	7	5	4	6
C01720	1	2	5	4	8	10	3	9	7	11	6
C02230	4	9	5	6	10	11	1	2	8	3	7
C00837	10	1	9	2	4	7	6	5	3	8	11
C13154	3	6	10	1	8	9	4	7	11	5	2
C00465	4	2	10	9	5	11	8	1	7	6	3
C13976	5	11	10	1	9	7	8	3	2	4	6
C03108	1	3	4	2	7	9	8	6	10	5	11
C13859	3	1	8	2	11	10	5	4	9	6	7
C16258	4	2	7	3	8	5	6	9	11	10	1
C03522	11	8	5	4	9	7	2	1	3	6	10
C05457	2	3	8	6	9	1	4	5	11	7	10
C00746	3	10	9	5	6	4	1	8	11	7	2
C13746	4	10	9	1	2	8	5	6	11	7	3
C08297	6	5	2	8	1	3	10	9	7	11	4
C12042	5	6	8	9	11	10	7	2	3	4	1
C10401	10	5	11	3	2	1	8	4	6	9	7
C10530	10	2	1	5	4	9	3	7	11	8	6
C09797	11	8	5	10	2	4	3	1	7	6	9
C09558	10	11	2	5	9	8	7	6	3	1	4
C12508	5	10	4	11	6	8	3	1	7	2	9
C01822	10	9	1	2	6	5	3	4	8	7	11
C02204	1	2	8	5	4	6	11	10	9	7	3
C14489	3	9	5	2	8	11	6	7	1	4	10
C10297	11	10	1	5	8	3	9	6	7	4	2
C04167	2	6	11	8	9	3	5	7	4	1	10
C08285	2	8	6	1	4	10	9	11	3	7	5
C08255	1	2	8	7	5	6	10	11	3	4	9
C03065	5	6	4	2	8	1	11	9	7	10	3
C08469	11	1	10	4	5	3	9	6	8	2	7
C15673	1	2	8	6	9	5	4	10	11	7	3
C13973	10	9	1	11	6	5	7	8	3	2	4
C07222	10	3	11	2	5	8	4	6	7	1	9
C09918	5	11	9	3	6	1	10	7	8	4	2
C08842	1	7	5	6	10	3	11	2	8	9	4
C10988	11	3	10	9	5	2	6	8	4	1	7
C07576	5	10	1	11	3	8	7	4	2	9	6

C06788	6	7	2	5	8	11	10	9	4	3	1
C14552	3	5	10	1	9	11	4	8	6	2	7
C09784	10	11	8	6	2	4	3	5	9	7	1
C07664	9	10	5	8	11	6	1	2	3	7	4
C09650	11	9	6	8	1	3	10	5	2	7	4
C09783	1	10	5	9	4	8	6	11	3	7	2
C06734	8	2	9	6	7	10	11	3	4	5	1
C11003	11	2	9	4	7	10	8	6	3	5	1
C11781	10	11	1	2	8	5	7	3	6	4	9
C02162	10	11	2	3	9	8	1	4	7	6	5
C10880	11	3	10	5	8	9	2	6	1	7	4
C14430	11	10	9	2	7	4	3	5	6	8	1
C03465	5	8	6	2	1	10	11	4	9	3	7
C06910	5	4	10	3	9	11	2	8	6	7	1
C08626	10	3	9	11	2	5	8	6	1	4	7
C09255	5	11	2	3	9	6	1	4	8	7	10
C10331	11	10	5	8	9	3	1	6	2	4	7
C13048	10	11	9	8	1	5	3	4	6	7	2
C07589	3	11	10	5	1	9	2	6	7	8	4
C10562	10	5	8	3	2	1	11	9	6	4	7
C11129	5	10	8	11	2	6	4	7	3	1	9
C02862	3	9	5	1	8	2	7	11	6	10	4
C07378	10	5	1	11	8	4	7	6	3	9	2
C03486	4	3	5	2	9	1	7	6	11	8	10
C11014	11	5	4	9	1	3	2	8	7	10	6
C07180	10	5	11	3	6	8	9	7	4	1	2
C10822	3	1	9	4	10	6	2	7	5	11	8
C10340	11	8	2	1	5	4	10	3	7	6	9
C11621	10	3	11	8	5	2	4	1	9	7	6
C08531	11	10	2	1	6	3	9	5	4	8	7
C03199	3	2	8	5	9	6	4	7	11	1	10
C03500	11	9	1	5	3	6	10	7	2	8	4
C14053	10	5	7	4	11	8	3	1	9	6	2
C13645	1	2	11	8	5	6	4	3	10	9	7
C08155	3	1	8	4	11	7	6	9	5	2	10
C10565	10	6	1	9	5	2	7	3	11	8	4
C06353	1	10	2	5	8	6	9	3	11	4	7
C02224	5	2	3	6	11	1	4	10	7	8	9
C14713	11	8	5	10	6	2	1	9	3	4	7
C15559	2	4	7	3	8	5	11	1	9	10	6
C07849	10	5	4	6	2	11	8	3	9	1	7
C09164	5	10	8	2	1	4	3	6	7	9	11
C10470	10	5	11	8	3	1	2	4	9	6	7

C13699	1	11	2	6	5	10	8	3	4	9	7
C03365	1	10	2	5	8	6	9	3	11	7	4
C05076	8	5	1	9	11	2	10	3	7	6	4
C15679	9	1	3	4	5	2	6	11	8	10	7
C10019	10	3	8	2	11	9	4	6	1	7	5
C07620	11	3	10	2	1	4	6	5	8	7	9
C12217	2	8	1	11	5	6	10	4	9	7	3
C08654	10	8	11	5	6	3	9	2	4	7	1
C10573	4	11	1	6	8	9	3	5	7	10	2
C13863	1	8	2	5	10	4	3	7	9	6	11
C15195	3	10	1	4	9	8	5	2	11	6	7
C04201	7	2	4	8	6	9	10	11	1	3	5
C09294	5	6	8	1	2	4	11	9	3	10	7
C07608	10	5	9	2	8	6	11	1	4	7	3
C04101	1	10	7	8	2	11	4	5	3	6	9
C06694	10	9	5	4	3	6	1	7	8	2	11
C03663	8	11	10	2	3	5	7	4	1	9	6
C11042	11	8	5	1	6	2	3	4	9	7	10
C02669	1	2	6	8	10	3	11	4	5	9	7
C02705	1	3	2	7	4	8	10	5	11	9	6
C05324	8	5	1	10	9	11	6	7	4	2	3
C01670	10	5	11	6	4	1	3	2	9	7	8
C15819	1	11	2	9	7	6	4	3	5	10	8
C15234	11	5	8	4	2	6	7	9	1	3	10
C13796	6	5	8	1	10	2	3	4	11	9	7
C09744	8	10	9	1	3	4	2	11	5	7	6
C10287	10	7	4	9	2	11	3	8	6	1	5
C07056	5	10	3	4	2	1	9	11	6	8	7
C03822	1	8	9	2	11	5	6	10	4	3	7
C01744	5	10	11	8	2	9	1	3	6	7	4
C13112	11	4	9	10	5	6	1	3	8	2	7
C08672	9	7	1	4	2	3	10	6	8	5	11
C06421	1	2	3	5	8	9	6	4	10	11	7
C02640	5	10	2	9	6	1	8	3	7	4	11
C04630	6	8	1	5	2	10	11	3	9	7	4
C08151	3	1	11	9	7	2	6	10	8	5	4
C07912	5	10	8	4	3	2	9	1	11	7	6
C15311	6	5	8	9	4	1	3	11	7	10	2
C14418	11	5	2	1	8	7	6	3	4	10	9
C09430	10	1	8	7	2	9	5	11	4	3	6
C01736	4	5	9	11	10	1	2	8	7	6	3
C15364	3	2	10	5	11	8	6	7	1	9	4
C14240	11	3	7	8	6	2	1	9	4	10	5

C13548	8	11	10	7	6	2	1	3	4	9	5
C04686	3	5	11	4	7	2	10	1	8	6	9
C01657	11	5	8	4	10	9	6	7	3	2	1
C08211	3	8	2	7	9	4	1	5	11	6	10
C09489	9	10	3	8	1	4	2	11	6	5	7
C05574	11	10	9	1	8	3	4	5	2	7	6
C01375	5	8	1	3	11	9	2	10	7	4	6
C14564	11	7	6	4	1	10	5	3	2	9	8
C13851	11	5	2	1	10	8	3	4	6	7	9
C08797	10	9	1	11	5	6	3	2	8	7	4
C03947	3	8	2	1	5	6	11	10	9	4	7
C11822	2	5	6	8	3	4	1	10	11	7	9
C00102	1	8	9	2	11	5	6	10	4	3	7
C12978	3	9	8	10	5	6	2	7	11	1	4
C07317	11	1	3	10	5	4	9	8	2	6	7
C01444	2	1	5	4	11	6	8	3	7	9	10
C14079	10	5	11	8	2	6	1	3	9	4	7
C07466	3	2	4	8	1	6	5	7	11	10	9
C10949	11	2	6	4	5	1	10	9	3	8	7
C06539	10	5	11	4	2	9	6	1	3	8	7
C11602	9	10	11	1	8	3	7	4	6	2	5
C02496	1	2	5	6	8	11	10	4	3	9	7
C09144	10	5	6	8	7	9	3	11	2	4	1
C07913	5	8	6	11	1	4	3	10	7	9	2
C02744	2	4	7	8	3	5	10	11	6	1	9
C10363	10	9	4	7	2	3	1	6	8	11	5
C04256	1	2	4	9	10	7	3	5	6	11	8
C13907	2	5	1	9	8	6	4	3	11	7	10
C10062	1	10	6	5	7	8	2	11	4	3	9
C01405	3	1	10	5	8	2	11	9	6	4	7
C14609	3	4	8	9	7	6	11	1	10	5	2
C09025	10	5	8	1	2	4	11	3	6	7	9
C09344	9	7	11	2	10	3	6	5	1	4	8
C08704	9	5	6	3	1	11	10	4	8	7	2
C06877	9	10	6	5	11	2	8	4	3	7	1
C14486	3	5	9	6	2	1	11	4	10	8	7
C07464	5	10	3	1	11	6	2	8	4	9	7
C12110	8	5	6	1	2	10	4	9	11	7	3
C09832	10	3	11	7	2	8	9	1	5	6	4
C11318	10	11	5	1	2	8	4	3	6	9	7
C11264	10	3	9	4	1	6	2	11	7	8	5
C07909	9	5	11	10	6	1	8	2	4	3	7
C08334	6	1	10	5	8	9	2	11	7	3	4

C14244	11	8	5	7	1	2	10	6	3	4	9
C04203	11	2	8	6	5	10	1	9	4	3	7
C02147	8	5	6	2	1	11	10	3	9	4	7
C10534	10	11	2	3	7	5	9	1	4	6	8
C04589	7	4	2	8	10	9	1	3	11	5	6
C07655	4	10	11	8	3	6	7	2	5	1	9
C06263	11	2	8	5	6	1	4	7	3	10	9
C12630	10	5	1	9	8	3	7	6	2	11	4
C02261	5	8	2	6	1	10	11	9	4	7	3
C13192	2	5	4	1	6	10	3	7	8	11	9
C13194	2	5	11	1	4	7	6	8	10	3	9
C04144	2	5	9	6	7	10	1	8	4	11	3
C13826	11	2	5	9	6	1	10	7	3	4	8
C10274	10	11	9	5	6	2	3	7	8	4	1
C03357	2	6	1	8	5	9	7	3	4	11	10
C07143	10	5	7	11	1	4	3	8	9	6	2
C00548	11	5	2	9	4	8	3	10	6	1	7
C10221	10	11	1	5	3	4	9	7	6	8	2
C08451	2	5	10	3	6	11	1	9	4	8	7
C08149	3	9	11	1	8	10	5	2	6	7	4
C03493	5	1	6	8	9	11	10	2	3	4	7
C13691	5	10	2	4	1	9	11	3	6	7	8
C08992	10	11	6	7	8	3	5	9	1	2	4
C10574	3	5	10	4	1	2	7	8	11	9	6
C11653	1	9	3	10	7	2	6	5	11	4	8
C10371	10	11	2	6	3	9	7	5	1	4	8
C01963	1	3	8	6	2	4	11	10	5	7	9
C07043	9	8	11	10	5	7	3	1	2	4	6
C10561	10	5	3	11	8	4	7	9	2	1	6
C04544	2	4	6	9	8	1	10	7	5	3	11
C14481	3	11	7	4	10	8	5	6	2	1	9
C02379	6	5	2	3	1	9	8	11	4	7	10
C02993	10	6	9	4	5	2	8	11	3	1	7
C09926	10	11	6	1	2	5	4	3	9	7	8
C09902	9	8	2	11	3	10	1	5	7	4	6
C07261	11	5	10	1	2	3	9	4	8	7	6
C14470	11	8	1	3	9	10	5	2	6	4	7
C09249	10	5	6	9	8	3	4	11	2	7	1
C12768	10	5	7	1	4	2	11	3	9	8	6
C08114	9	10	11	6	2	5	7	4	3	8	1
C06928	3	10	5	4	6	8	11	7	9	2	1
C09866	1	10	6	9	4	8	7	3	11	5	2
C09283	10	11	4	8	2	3	6	7	9	1	5

C09365	10	8	5	11	6	9	3	4	1	2	7
C03832	6	8	5	1	2	10	11	4	3	9	7
C09399	10	11	2	7	4	8	3	5	9	6	1
C11360	10	11	5	3	9	8	2	6	4	1	7
C07348	2	8	9	7	5	10	3	6	4	11	1
C09692	10	1	8	11	9	3	6	4	2	5	7
C06519	10	5	9	6	4	1	8	11	3	7	2
C14136	3	11	10	8	1	4	9	6	7	2	5
C14726	11	3	8	7	5	1	9	2	10	4	6
C15537	3	9	5	11	2	6	8	1	10	4	7
C10600	5	6	10	8	1	2	3	9	11	7	4
C07487	11	8	10	5	3	2	1	6	9	7	4
C11820	5	10	8	4	7	9	2	3	6	11	1
C14311	11	5	10	8	2	4	7	3	1	6	9
C07515	2	5	6	10	8	11	1	3	4	9	7
C00845	2	8	4	9	3	10	5	1	6	7	11
C04234	2	1	8	7	3	5	10	4	11	6	9
C10750	10	8	5	6	7	9	3	4	11	1	2
C08661	9	5	7	4	2	1	3	10	8	6	11
C05342	4	5	9	8	11	7	3	6	1	10	2
C06821	1	2	3	5	10	4	6	11	8	9	7
C10057	10	9	11	5	6	4	3	8	7	1	2
C01382	1	5	8	2	6	11	7	10	3	9	4
C08670	9	10	2	1	4	6	8	3	11	5	7
C12569	1	2	11	5	6	10	8	3	4	7	9
C11171	3	1	8	2	5	6	7	4	9	11	10
C11273	10	3	9	11	5	1	6	8	4	2	7
C10725	10	11	4	6	8	1	3	7	5	9	2
C01865	1	2	8	6	5	3	4	10	9	11	7
C05118	5	8	1	3	9	2	6	4	11	10	7
C09187	3	7	8	9	1	5	2	6	11	4	10
C07106	5	10	1	11	2	6	9	7	4	8	3
C08965	3	4	5	2	9	11	8	6	10	7	1
C14553	3	10	9	4	11	5	7	6	2	8	1
C08238	1	10	7	6	3	9	2	4	5	11	8
C02052	1	2	10	3	11	8	9	7	5	4	6
C08332	5	11	8	10	9	1	2	6	4	7	3
C10974	2	8	10	5	6	4	1	11	7	3	9
C12294	9	11	5	10	2	8	1	3	7	4	6
C09931	1	4	5	6	2	11	10	3	8	9	7
C11190	10	9	8	6	11	1	2	5	4	3	7
C11040	11	6	1	10	2	8	3	5	4	9	7
C00898	1	2	5	8	11	4	3	10	6	9	7

C03126	1	5	2	3	10	4	9	8	11	6	7
C10398	11	3	10	8	5	2	1	6	7	4	9
C07313	1	3	5	2	8	10	9	6	11	4	7
C06894	10	9	6	5	8	4	1	7	3	11	2
C06737	2	8	3	10	5	1	11	9	7	6	4
C02657	6	5	2	11	1	9	3	8	4	10	7
C11230	9	11	4	10	8	1	2	5	6	3	7
C09563	9	10	5	8	11	1	6	3	7	2	4
C09383	10	5	9	4	1	3	2	6	7	8	11
